# Effectiveness of a community health worker-led low-sodium salt intervention to reduce blood pressure in rural Bangladesh: protocol for a cluster randomized controlled trial

**DOI:** 10.1186/s13063-023-07518-3

**Published:** 2023-07-27

**Authors:** Andrew Y. Chang, Mushfiqur Rahman, Animesh Talukder, Humyra Shah, Malay Kanti Mridha, Mehedi Hasan, Malabika Sarker, Pascal Geldsetzer

**Affiliations:** 1 Department of Epidemiology and Population Health, Stanford University School of Medicine, Stanford, USA; 2 Center for Innovation in Global Health, Stanford University, Stanford, USA; 3 Stanford Cardiovascular Institute, Stanford University, Stanford, USA; 4 BRAC James P Grant School of Public Health, BRAC University, Dhaka, Bangladesh; 5 Heidelberg Institute of Global Health, Heidelberg University, Heidelberg, Germany; 6 Division of Primary Care and Population Health, Department of Medicine, Stanford University School of Medicine, Stanford, USA; 7 The Chan Zuckerberg Biohub, San Francisco, USA

**Keywords:** Blood pressure, Hypertension, Sodium, Low-sodium salt substitute, Community health worker, Global health, South Asia, Bangladesh

## Abstract

**Background:**

High blood pressure is a major public health problem in low- and middle-income countries. Low-sodium salt substitute (LSSS) is a promising population-level blood pressure-lowering intervention requiring minimal behavioral change. The optimal method of delivering LSSS to individuals, however, is currently unknown. Community health workers (CHWs) have successfully been used to implement health interventions in Bangladesh and may provide a venue for the dissemination of LSSS.

**Methods:**

We aim to conduct a cluster-randomized controlled trial involving 309 households in rural Bangladesh previously identified and characterized by the BRAC James P Grant School of Public Health, BRAC University (BRAC JPGSPH). These households will be randomly assigned to three arms: (1) control, i.e., no intervention; (2) information only, i.e., community health workers will provide basic information on high blood pressure, the health consequences of excessive salt consumption, and feedback to the participant on the likely quantity of salt s/he consumes (estimated using a questionnaire); (3) free LSSS arm: the same information as in arm 2 will be provided, but participants will receive 6 months of free low-sodium salt along with education on the benefits of LSSS. One male and one female adult (age ≥ 18 years) in each household will be invited to participate, the exclusion criteria being households with members known to have high serum potassium levels, are taking medications known to elevate potassium levels (e.g., ACE inhibitors, ARBs, potassium-sparing diuretics), are already taking potassium supplements, or those who have known kidney disease or abnormal serum creatinine at baseline. The primary endpoint will be blood pressure at 6 months post-intervention.

**Discussion:**

Recent large clinical trials of LSSS in China and India have shown not only blood pressure improvements, but also stroke, major cardiac event, and all-cause mortality reductions. Nevertheless, how to best translate this intervention to population-level effectiveness remains unclear. Our study would test whether a community health worker-based program could be effectively used to disseminate LSSS and achieve measurable blood pressure benefits.

**Trial registration:**

ClinicalTrials.gov NCT05425030. Registered on June 21, 2022.

**Supplementary Information:**

The online version contains supplementary material available at 10.1186/s13063-023-07518-3.

## Introduction

### Background and rationale 

High blood pressure, which is already more common in low- and middle-income countries (LMICs) than in high-income countries, is responsible for a significant burden of cardiovascular diseases (myocardial infarctions (MIs, i.e., heart attacks), strokes), and renal failure in these settings [1, 2]. Over the past several decades, cardiovascular disease has become the leading cause of mortality in LMICs, and this problem is only expected to increase in LMICs such as Bangladesh due to rapidly shifting demographic trends including population aging, dietary changes, and sedentary lifestyles [3–5]. Regrettably, LMIC public health systems are not prepared to prevent and control the epidemic of cardiovascular diseases. Furthermore, those living in poor countries usually lack the financial means to treat the devastating sequelae of cardiovascular risk factors [6, 7].

Blood pressure reduction represents an attractive target for intervention, as it is associated with heart attacks and stroke in a log-linear fashion down to a systolic and diastolic BP of 115/75 mm Hg [8]. This implies that reductions in blood pressure in the general population, rather than just among those above the clinical threshold for hypertension, would lead to important health benefits. Despite the availability of proven, effective medications, reducing blood pressure in LMICs is proving difficult, however, for a number of reasons. These include problems with early detection of elevated blood pressure, ensuring that antihypertensive medications are readily available and affordable to patients, and convincing patients to take (and frequently pay for) medications long-term for an asymptomatic disease [9]. Furthermore, nonpharmacologic interventions for lowering blood pressure such as increasing people’s physical activity and improving their dietary habits to reduce sodium intake (the primary nutritional driver of hypertension) have generally proven to be difficult even in high-resource settings. This has been due to several causes including a persistent and prevalent “obesogenic” environment and overemphasis on medical procedures and drug therapy (over lifestyle and behavioral interventions). Moreover, the mental fatigue of sustaining healthy habits and social acceptance of maladaptive behaviors lead to poor durability of lifestyle changes after such interventions are introduced [10, 11].

Given these issues, low-sodium salt, a compound in which 25 to 75% of the sodium chloride (NaCl) is replaced by potassium chloride (KCl) and/or magnesium sulfate (MgSO_4_), is an attractive intervention for blood pressure reduction, as it avoids several of the previously-mentioned issues of lifestyle change challenges:

First, little behavior change is needed: As long as the non-sodium portion of the salt does not exceed one-third of the product, the taste of the low-sodium salt product is indistinguishable from regular salt, and in some studies in LMICs, blinded subjects preferred the low-sodium salt [12–14]. It can be used in the same manner as regular salt in cooking and seasoning foods by usual cultural practices in such settings [13].

Furthermore, little additional cost is imposed upon the consumer: KCl and MgSO_4_ are cheap, readily-available minerals/nutritional supplements (with their own health benefits) with similar costs as regular salt.

Finally, no special technologies or advanced medical oversight for intervention delivery are needed: No physician supervision, special preparation equipment, or monitoring systems are necessary for the use of low-sodium salt by the target audience [15].

Overall sodium intake is also often higher in LMICs than high-income countries—for example, the average daily salt intake in German adults was estimated to be 8.4–10 g/day [16]. In adults in Bangladesh, it may be as high as 17 g/day [17]. A large proportion of salt intake in LMICs tends to be from home-prepared meals rather than ready-made processed foods: Compared to high-income countries, a greater proportion of food consumed in LMIC settings (including Bangladesh) is prepared within the home, particularly in rural areas, thus increasing the potential benefits of the intervention.

Recent large clinical trials of LSSS in China and India have shown not only blood pressure improvements, but also stroke, major cardiac event, and all-cause mortality reductions in randomly-assigned users [18, 19]. Despite these impressive findings, there is a gap in the literature, as such RCTs have relied upon trial staff to provide LSSS free of charge to participants as part of standardized experimental protocols. Such personnel are not health workers who would otherwise be available to existing LMIC health systems. As such, how to best translate the LSSS intervention to population-level effectiveness in such settings remains unclear. Innovative delivery mechanisms such as educational campaigns and directed provision by local health workers have been effectively used in LMICs to disseminate other behavioral interventions in primary care [20–22]. Additionally, community health workers are increasingly proving to be a powerful resource for the delivery of care for both noncommunicable diseases and primary care in LMICs [20, 22, 23]. They are cost-effective, respected in their home communities, and fill a much-needed healthcare personnel gap in rural/remote regions of countries where nurse and physician staffing cannot be reliably guaranteed [24–27]. As such, we hypothesized that CHWs may represent a promising means for spreading knowledge and acceptance of LSSS in LMIC settings, bridging the gap in knowledge between idealized RCT results and a delivery mechanism for this population-level intervention.

In Bangladesh, the setting of this proposed study, there exists a robust CHW network which has successfully delivered many public health interventions in a variety of regional and local contexts [23, 25–27]. Therefore, findings from our RCT would be immediately actionable through extant CHW programs within both the study community as well as the country at large.

### Objectives 

1. To determine the effect on blood pressure of a community health worker-based education program providing information about ways to reduce one’s salt intake and the health benefits of doing so.
2. To determine the effect on blood pressure of a community health worker-based program providing low-sodium salt directly to households in addition to information about ways to reduce one’s salt intake and the health benefits of doing so.

### Trial design

The design of this RCT is a cluster-randomized, parallel-group superiority study. The unit of randomization will be a standard household, which is defined by the WHO STEPS Noncommunicable Disease Risk Factor Survey as “a dwelling in which persons, either related or unrelated, live together and take food from the same kitchen.” Participants will be randomized into one of three study arms as further detailed below:

## Methods: participants, interventions, and outcomes

### Study setting

Our study setting is in Rampur Union, Parbatipur, a rural/semi-rural sub-district in Dinajpur District (Rangpur Division), in northern Bangladesh. Within Parbatipur, BRAC has assessed approximately 8000 households for noncommunicable disease risk factors as part of a larger Wellcome-trust funded multinational biobank (involving those living in Bangladesh, India, Pakistan, and Sri Lanka) to better understand the patterns and determinants of cardiovascular health in South Asian people in a cross-sectional analysis [28, 29]. To avoid spillover, every fifth household will be approached during the enrollment phase to minimize neighborhood effects. Specifically, the areas of Uttor Basupara, Basupara, and Mondolpara, which are adjacent to one another with a similar socio-economic makeup within Rampur Union, will be approached for enrollment.

### Eligibility criteria

One male and one female adult (age ≥ 18 years) in each study household will be invited to participate. The exclusion criteria are subjects known to have high serum potassium levels, are taking medications known to potentially induce hyperkalemia (potassium-sparing diuretics, Angiotensin-converting enzyme (ACE) inhibitors, Angiotensin II receptor blockers, digoxin), are already taking potassium supplements, or those who have known kidney disease at baseline. Additionally, all adults residing in the selected households initially screened for intervention will undergo serum creatinine testing, with those with estimated glomerular filtrate rate (eGFR) values < 60 ml/min/body surface area excluded from the intervention but kept in the intervention group by intention-to-treat principles to avoid breaking of randomization. Although minors will not be involved in the study, to avoid spillover injury to children in the intervention households, all members of intervention households under the age of 18 will also undergo urine dipstick testing. If proteinuria (as +  + protein on urinalysis) is detected on two separate tests spaced three days apart, the household will be excluded from receiving the LSSS intervention (but followed in the intervention arm to avoid breaking randomization).

Lastly, potential participants (but not households) will be excluded if they have (1) a terminal illness, (2) are substantially mentally disabled (and thus unable to give consent), or (3) are bedridden.

### Who will take informed consent?

Fieldworkers will provide all potential participants in the study with a consent form to read. The document will contain multiple diagrams and illustrations to explain the key concepts of the project. For illiterate participants, the fieldworkers will read the consent form in the native spoken language of the participants, as necessary. The consent form will outline the scope, purpose, and procedures of the study as well as the risks and benefits of study participation. The fieldworkers will answer any questions before asking for consent. If the potential participant meets the inclusion criteria (and the other members of the household do not meet the exclusion criteria) and wishes to participate, she or he will be asked to sign or thumbprint the consent form; the fieldworker will then countersign. If the participant is illiterate, a witness will sign in addition to the participant’s fingerprint. One signed consent form remains with the participant; a second signed consent form is retained by the study team. All study subjects will be informed that they may withdraw consent to participate in the trial at any point during the study.

### Additional consent provisions for collection and use of participant data and biological specimens

Participants will be informed that data collected from the trial may be used in ancillary studies or secondary analyses. As noted above, blood and urine will be obtained during the screening process to exclude those with preexisting renal dysfunction.

## Interventions

### Explanation for the choice of comparators

Our three study arms were chosen to determine (1) If a community health worker-administered intervention can be effectively used to disseminate LSSS, and (2) If such a program can lead to measurable blood pressure reduction. Thus, the control arm involves no intervention, while one intervention arm involves a community health worker-led education/counseling program and the other intervention arm involves community health worker-led education/counseling as well as free provision of LSSS.

### Intervention description

1. Basic Information/Education Intervention Arm: Community health workers will provide basic information and education on (i) the health consequences of high blood pressure and excessive salt consumption, (ii) feedback to the participant on the likely quantity of salt s/he consumes (estimated using a questionnaire), and (iii) advice on reducing salt consumption (Supplementary Material 1). This arm will allow for isolating the treatment effect of CHW-delivered participant education/information about hypertension and salt consumption alone.
2. Information plus home delivery of free low-sodium salt intervention arm: in addition to the information intervention from arm 2, each household will be given a parcel (of approximately 1–2 kg) of low-sodium salt (Supplementary Material 2) on a monthly basis for free during community health worker household visits. This arm will serve to isolate the treatment effect of low-sodium salt itself.

### Criteria for discontinuing or modifying allocated interventions

The intervention arm involving free provision of LSSS product will be discontinued if a participant develops high blood potassium levels (hyperkalemia) or ends up meeting the exclusion criteria (i.e., develops kidney disease or is prescribed potassium supplements or potassium-elevating medications by their healthcare provider) at any point during the study. Any and all patients may discontinue participation in the trial at any time of their own volition.

### Strategies to improve adherence to interventions

As the present trial is testing the effectiveness of the delivery mechanism for LSSS, no specific strategies to improve adherence to the product will be undertaken for the purposes of encouraging adherence. All participants randomized to the free LSSS arm, however, will be asked not to share the product with other households to avoid cross-contamination. To monitor for compliance, intervention arm community health workers will measure any unused LSSS at each monthly visit.

### Relevant concomitant care permitted or prohibited during the trial

All participants will be encouraged to continue usual health care during the span of the trial. No changes should be made in their routine healthcare screening or therapy—rather, any participants found to have developed exclusion criteria medical conditions during the study will be removed from the free LSSS intervention arm.

### Provisions for post-trial care

The existing literature suggests that low-sodium salt substitutes provide beneficial effects at best and a neutral (or null) effect at worst. The product is already commercially available for purchase over the counter in other nearby countries and is used in many nations as a nutritional supplement without the requirement of physician or healthcare provider supervision.

### Outcomes

The primary outcome variable of our study is systolic blood pressure (SBP) as a continuous variable.

Secondary outcome variables will include:

1. Diastolic blood pressure (DBP)
2. Hypertension (defined as a systolic blood pressure ≥ 140 mm Hg or diastolic blood pressure ≥ 90 mm Hg or reporting to be taking antihypertensive medication)
3. Systolic blood pressure among adults who had hypertension at the time of the baseline survey
4. Diastolic blood pressure among adults who had hypertension at the time of the baseline survey
5. Hypertension control (defined as a systolic blood pressure < 140 mm Hg and a diastolic blood pressure < 90 mm Hg) among adults who had hypertension at the time of the baseline survey
6. Self-reported sodium intake (as estimated using typical daily dietary recall of both added salt and processed food/high salt food products)
7. Self-reported low-sodium salt intake (as estimated using typical daily, 1-week, and 1-month recall)

### Participant timeline

The time schedule of enrolment, interventions, assessments, and visits for participants is provided via the schematic diagram below. There are no washout or crossover periods in this study.

**Table:** 

	**Study period**								
	**Enrolment**	**Allocation**		**Post-allocation (in months)**					**Close-out**
**Timepoint**	*** − t***_***1***_	**0**	***t***_***1***_	***t***_***2***_	***t***_***3***_	***t***_***4***_	***t***_***5***_	***t***_***6***_	***t***_***7***_
**Enrolment:**									
**Eligibility screen**	60 days								
**Informed consent**	5 days								
***Baseline survey***	15 days								
**Allocation**		5 days							
**Interventions:**									
***Control group***									
***Education/information arm***			X	X	X	X	X	X	
***Free LSSS arm***			X	X	X	X	X	X	
**Assessments:**									
***Baseline survey***	X	X							
***Endline survey***									X
***LSSS interim use measurement***			X	X	X	X	X	X	X

## Sample size

Assuming a Type I error rate of 0.05 and a desired power of 0.80 for our study, we hypothesize an intervention effect of 2.0 mm of mercury (mm Hg) of systolic blood pressure reduction with standard deviation of 20.0 mm Hg. These values were extrapolated from prior RCTs of low-sodium salt interventions in LMICs, whose effect sizes have varied from 1.1 to 10 mm Hg (with variance around the order of 20–30 mm Hg) with most studies finding effect sizes of over 3.0 mm Hg in blood pressure reduction [30–37]. Uptake of the intervention in these studies ranged from 74.3 to 94.1%.

Assuming an analysis of simple difference of outcome, a conservative intra-cluster coefficient (ICC) of 0.25 (and an expected cluster membership of five adults per cluster), we estimated an average of 33 clusters would be necessary per study arm to detect an effect size of our proposed magnitude. This would suggest that a total enrollment of 660 study subjects would be needed for our study.

Below, a table (generated from the National Institutes of Health Sample Size Calculator for GRTs [group- or cluster-randomized trials]) noting the absolute detectable difference (in millimeters of mercury) for a range of cluster sizes by cluster numbers is presented:
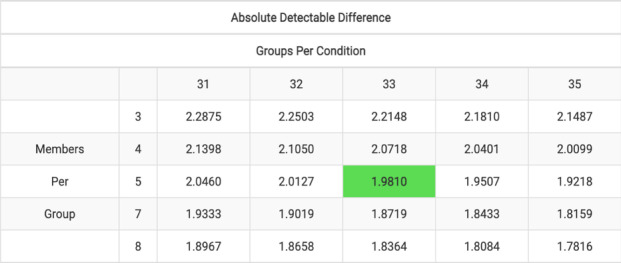


Of note, we will be obtaining blood pressure measurements at baseline. Thus, having the correlation between baseline and endline BP will provide additional power over this very conservative estimate.

### Recruitment

Given concerns for lower uptake among study participants randomized to the education only arm, we decided to build a conservative estimate approximated from the lowest value of uptake in the literature of free salt substitute of 75% [31, 38]. This would support the selection of a final sample size of 206 participants per study arm, leading to a total study recruitment requirement of 618 subjects. This is well within the 2,181 subjects currently registered within the BRAC cohort described above.

We shall recruit study households in the following stages: (a) the eligible households in the sampling frame will be listed against the inclusion criteria, i.e., households without any diagnosed case of chronic kidney disease (CKD), nephrotic syndrome, or hyperkalemia, no history of current intake of potassium supplement or potassium-elevating medications, and households consisting of at least two adults (aged ≥ 18 years) willing to participate in the trial; (b) from the eligible households, we shall choose a systematic random sample (with an interval of five houses) to create buffers among study households to reduce the chances of spill-over; (c) the systematic random sample of 309 households will be further inflated by the current CKD prevalence of 17.3% in Bangladesh; the resulting 362 households will then undergo the randomization process [39]. We will plan to exclude the households with CKD or nephrotic syndrome cases; to ensure this, we will perform serum creatinine tests among the adults and urine dipstick tests among those under 18 years of age with the exclusion criteria previously enumerated.

The aforementioned recruitment areas of Uttor Basupara, Basupara, and Mondolpara are adjacent to one another and are further divided into six regions. As homes within these regions are not evenly clustered in an area (and do not adhere to a geographic grid with convenient central points of interest), we will designate one end of the area as the starting point. The field workers will use a random number generator to select the first household, then move on to enrolling homes sequentially, maintaining a gap of five households between enrolled homes. This process will be continued until the end of an area is reached.

## Assignment of interventions: allocation

### Sequence generation

The allocation sequence will be generated using computer-generated random numbers. No stratification will be performed. To reduce the predictability of a random sequence, blocking will be performed via a separate document that is unavailable to the fieldworkers who enroll participants and/or assign interventions. The randomization allocation process will be performed by a separate team independent of the fieldworkers.

### Concealment mechanism

The allocation sequence will be implemented via a central telephone with the allocation sequence known only to the study team member responsible for the call in order to conceal the sequence until interventions are assigned.

### Implementation

The allocation sequence will be generated prior to study commencement by a study team member who will not be involved in the enrollment and assignment of participants to specific intervention arms. Fieldworkers trained in research methods and good clinical practice will enroll participants during a home visit. Afterwards, they will inform participants of the intervention arm they have been randomized to based on the assignment made by a non-fieldworker study team member (who will not be involved in the enrollment process).

## Assignment of interventions: blinding

### Who will be blinded

Blinding will be performed for the data analyst but not the intervention community health worker nor the participant (as it will not be possible to do so, given the nature of the intervention). Blinding will be achieved by number coding the intervention groups, with codes not shared with the analyst until the conclusion of data analysis.

### Procedure for unblinding if needed

Unblinding will be only permissible in the case that a participant suffers an adverse event and unblinding is necessary to provide them with appropriate care. These situations should be rare, as the community health workers providing the intervention are already not blinded and the participants themselves are not blinded (as the intervention does not allow for blinding of the participant or intervention delivery personnel).

## Data collection and management

### Plans for assessment and collection of outcomes

In the first stage of the primary intervention, fieldworkers will conduct a baseline survey of participant age, sex, educational/employment status, dwelling characteristics, household ownership of durable assets, and medical history (particularly noting pre-existing diagnoses of hypertension and cardiovascular disease). This is available as Supplementary Material 3. Typical daily dietary recall will also be collected to estimate daily baseline sodium intake. One-week and one-month dietary recall will also be collected to confirm the reliability of the 24-h recall (though emphasis will be placed on shorter recall timelines). Additionally, anthropometric measurements (i.e., body weight and height) of the participants will be recorded. Blood pressure will be measured by trained fieldworkers using Omron Series 3 automatic portable blood pressure cuffs (Omron Healthcare, Kyoto, Japan) while trial subjects are in the seated position in the left arm following 15 min of rest. Three measurements will be taken at least 5 min apart to ensure accurate capture, with the mean of the second and third measurements used in the final data analysis. Units of blood pressure will be recorded in millimeters of mercury.

Finally, in the end stage of the study, fieldworkers will conduct an endline survey of all participants to assess daily endline sodium intake (by daily dietary recall) and one-week dietary recall. Blood pressure will be measured again by the procedure described above.

### Plans to promote participant retention and complete follow-up

Given the nature of the study, aside from routine contact from the community health workers in the two intervention groups, no specific plans will be made to promote participant retention. Fieldworkers will perform a house visit to complete the follow-up to ensure minimum loss to follow-up. All primary and secondary outcome data will be collected for participants who discontinue or deviate from intervention protocols, given the analysis will be performed as intention-to-treat.

### Data management

#### Data quality assurance

Data quality will be ensured through multiple procedures of review and cross-checking.

#### Data quality assurance standards

Data collection performance will be checked every day by the supervisors while pretesting. Data collectors with low performance will be given an initial warning and will have to recollect the data and pre-test again. If a second incident occurs, the data collector will be terminated, and a different data collector will be assigned and trained to collect data.

#### Data security and management

Data security will be assured through different steps. At first, data will be directly sent to the server immediately following collection. In addition, as a contingency, all data collected in the server will be backed up to a drive. Daily backups will be kept on drive at the end of each day of data collection and be kept separate from the devices during collection. Furthermore, data collectors will send all collected data at the end of each day of data collection via server. Security details of the server will not be communicated to anyone outside the research team.

#### Roles and responsibilities of data collection supervisors

Data collection supervisors are responsible for checking data discrepancies on a daily basis and taking necessary actions. All filled questionnaires will be cross-checked by the data collection supervisors at the end of the day at field for internal validation. This will involve collecting data at the same time as the data collector and checking for discrepancies holding the supervisor’s data as standard. 20% of the data collected on any given day will undergo the agreement test. Data collectors will not be informed beforehand which household the test will be performed at. In case of any missing or inconsistent data, the respective households will be revisited by the field coordinator. Confidentiality will be maintained throughout the interview and analysis process, and de-identified data will be stored under password protection.

Data collected from the study will be housed physically at BRAC James P Grant School of Public Health (BRAC JPGSPH) in locked cabinets accessible only to the field research manager and the primary investigator. All study questions will be asked and responses collected using the SurveyCTO platform on handheld Android devices. Blood test reports for screening participants for renal disease will be obtained as physical printed report from the local clinical laboratory and manually entered into SurveyCTO. The physical reports will be destroyed at the end of the project duration. Aside from these and the signed consent forms, no other data will be captured on paper/physical media. At the conclusion of primary data collection, all data will be entered into an electronic SurveyCTO database with secure institutional Google Drive backup. The database will be password-protected, with the password known only to the two site primary investigators and the research manager. The BRAC JPGSPH site primary investigator and research manager will provide an electronic, de-identified, cleaned analysis dataset to the statistical team at the conclusion of the study. The SurveyCTO database will be kept active following the conclusion of the study for addressing long-term safety concerns of study participants.

### Confidentiality

No sensitive questions will be asked to the participants. The privacy and well-being of the participants will be prioritized if there are any unforeseen risks. Moreover, the interviews for enrollment and data collection will be conducted in a private setting to provide respondents with their rights to necessary privacy and breathing space.

Data collected from the study will be housed physically at BRAC JPGSPH in locked cabinets accessible only to the field research manager and the primary investigator. At the conclusion of primary data collection, all data will be entered into an electronic SurveyCTO database. The database will be password-protected, with the password known only to the two site primary investigators and the research manager. Aside from signed consent forms, no other data will be captured on paper/physical media. The BRAC JPGSPH site primary investigator and research manager will provide an electronic, de-identified, cleaned analysis dataset to the statistical team at the conclusion of the study. The SurveyCTO database will be kept active following the conclusion of the study for addressing the long-term safety concerns of study participants.

### Plans for collection, laboratory evaluation, and storage of biological specimens for genetic or molecular analysis in this trial/future use

Trained specialists will be recruited to collect blood specimens from adults residing in households of the salt-recipient participants. The samples will be stored by the hospital for the period of testing and destroyed once the reports are generated. Serum creatinine tests will be conducted on the collected blood samples.

Urine samples will be collected from children (under 18) residing in households of salt-recipient participants. Trained field workers will perform spot tests, and the samples will be destroyed once the results are recorded. These two tests will be conducted only once before the initiation of the intervention.

## Statistical methods

### Statistical methods for primary and secondary outcomes

Our primary outcome, systolic blood pressure (outcome 1), will be treated as a continuous variable. It will be analyzed using linear regression to estimate the magnitude of the treatment effect of each study arm intervention. The secondary outcomes of diastolic blood pressure (outcome 2), as well as SBP and DBP among hypertensive individuals (3 and 4), will similarly be treated as continuous variables, to be summarized using linear regression. The secondary outcomes of hypertension (2) and hypertension control (5) will be assessed as a binary variable, summarized as an odds ratio (OR) using logistic regression. Lastly, self-reported sodium intake (6) and low-sodium salt intake (7) will be treated as continuous variables and analyzed using linear regression.

In the primary analysis, we will regress the outcome onto a categorical variable for the study arm (control, or intervention arms 2 and 3). We will calculate three beta coefficients with corresponding p-values to estimate the magnitude of the treatment effect of each study arm (with units in millimeters of mercury). Namely, these three study/comparisons are (i) control vs. CHW education/information only; (ii) control vs. CHW education/information + direct LSSS provision; and (iii) CHW education/information only vs. CHW education/information + direct LSSS provision. The model will include baseline BP as a covariate for adjustment. Should there be variations in other baseline characteristics between groups, we will further adjust our model for residual potential confounding sources such as age, gender, and clinical comorbidities. Secondary analyses of continuous variables will be treated similarly. In the secondary analyses of binary variables, we will similarly regress the outcome over the categorical variable for the study arm and strata to yield odds ratios estimating likelihood of the outcome.

Prespecified subgroup analyses will include sex, age over 65 years, participants hypertensive at baseline, and individuals normotensive at baseline. We will test for significance in these subgroup analyses by the use of an interaction term between the study arm and the subgroup variable.

Standard errors will be adjusted for clustering using the robust sandwich estimator. *P*-values < 0.05 will be considered statistically significant for the purpose of hypothesis testing for the primary analysis. *P*-values < 0.05 will be considered statistically significant for secondary analyses of binary outcome variables. All primary and secondary analyses will be conducted “as randomized” (i.e., following the intention-to-treat principle).

### Interim analyses

We do not plan to take any interim measurements between enrolment and the endline survey. As such, no interim analyses or stopping guidelines are planned, as there is no endpoint to base a stopping rule upon. Furthermore, as our intervention is a dietary supplement available in the open market that does not require a physician’s prescription, we had no basis to suspect that there would be safety outcomes to obtain in interim analyses. This decision was supported by the fact that larger randomized clinical trials elsewhere in the LMIC setting testing a similar LSSS product failed to detect harm from the intervention [18, 19]. This is in the setting of our exclusion criteria being even more restrictive than these prior robust RCTs.

### Methods for additional analyses (e.g., subgroup analyses)

Subgroup analyses planned include assessing blood pressure improvement and hypertension control in participants who were identified as hypertensive at baseline during the initial survey. The same statistical tests noted above will be performed for this subgroup. As stratification will not be employed at randomization for these participants, all findings in this group will be considered hypothesis-generating and non-causal.

### Methods in analysis to handle protocol non-adherence and any statistical methods to handle missing data

Analyses will be performed on an intention-to-treat basis. If missing data is < 5%, complete case analysis will be utilized. For missingness greater than that, multiple imputation will be utilized by chained equations.

### Plans to give access to the full protocol, participant-level data, and statistical code

Deidentified datasets analyzed during the current study and statistical code are available from the corresponding author on reasonable request, as is the full protocol. Public access will not be granted to the full participant-level dataset, but the full protocol and statistical code will be available to the public upon request to the investigators. Following the publication of the primary analysis, a de-identified version of the dataset, along with the statistical code will be publicly available via the Stanford Digital Repository.

## Oversight and monitoring

### Composition of the coordinating center and trial steering committee

The coordinating center will be based at the BRAC James P Grant School of Public Health, BRAC University (BRAC JPGSPH). As previously noted above, BRAC JPGSPH has already worked with the target population community, with many of the potential study participants previously enrolled into research studies by the institution as part of a larger Wellcome-trust funded multinational biobank to better understand the patterns and determinants of cardiovascular health in South Asian people in a cross-sectional analysis. BRAC JPGSPH also has substantial experience working with and evaluating interventions using community health workers. The BRAC JPGSPH team involves multiple investigators with extensive experience in epidemiology, noncommunicable disease research, community health research, and clinical trials in this setting. As such, the BRAC JPSGSPH coordinating center team’s role will be as equal collaborators on the study design and primary team for the on-the-ground subject recruitment, personnel management, and trial protocol enactment. This coordinating center will also be the principal team for liaising with and disseminating the results of the study to local and national stakeholders.

These members will supervise the day-to-day operations of the present study, including:

1. Drafting, revision, and defense of study protocols and Bangladesh-level human subjects research ethical approval protocols;
2. Recruitment, training, equipment, and evaluation of study fieldworkers;
3. Recruitment, training, equipment, and evaluation of study intervention community health workers;
4. Identification, testing, and purchase of intervention LSSS product;
5. Recruitment of study subjects;
6. Randomization of study subjects (including generation and implementation of the randomization allocation sequence);
7. Primary data collection, security, and integrity confirmation. This process will involve data de-identification and management as well.

BRAC JPGSPH study team members will also be involved in the entire data analysis and publication/results dissemination phase and will be primarily responsible for the dissemination of results within Bangladesh and with national stakeholders (e.g., the Ministry of Health).

The Stanford University School of Medicine team also has substantial experience in epidemiology, chronic disease research, and clinical trials in the global health arena. The role of this center will be to provide logistical guidance on protocol design and primary statistical analysis of the resultant data. The members of the team will be responsible for:

1. Drafting, revision, and defense of study protocols and obtaining human subjects research ethical approval protocols at Stanford;
2. Assistance with drafting and revising documentation used for the recruitment, training, equipment, and evaluation of study fieldworkers;
3. Assistance with drafting and revising documentation used for recruitment, training, equipment, and evaluation of study intervention community health workers;
4. Performing primary data analysis on the de-identified final dataset;
5. Drafting of abstracts and publications resulting from the findings of the primary data analysis;
6. The Stanford-based team will be responsible for dissemination of results within Stanford and local stakeholders (e.g., the King Center on Global Development).

Meetings within each center will happen on a regular basis (weekly to monthly) with ad hoc meetings as unexpected issues may arise. Teams from both institutions will meet in full at the start and close of the trial, as well as following primary data analysis. Otherwise, individuals from each team will meet as necessary virtually throughout the duration of the study, from inception/planning through publication/dissemination of results. No specific steering committee will be appointed, to allow for rapid vertical transmission of information as needed and for all members of the study team to voice concerns or opportunities for improvement of the trial freely at any time.

### Composition of the data monitoring committee, its role and reporting structure

The study will not include a data monitoring committee (DMC) separate from the study team because outcome data will only be captured at the baseline and endline of the study. The study team members responsible for monitoring these data (as described above in the data collection procedure) will, however, be independent from the sponsor of the trial and of competing interests.

### Adverse event reporting and harms

Although the existing literature suggests that low-sodium salt substitutes provide beneficial effects at best and a neutral (or null) effect at worst, given a new product is being introduced into a vulnerable population, all study staff will be trained to elicit and report any potential adverse events and side effects to the study coordinators. Participants will subsequently be referred to appropriate healthcare facilities for follow-up care. In addition, to reduce the chance of transmission of the novel coronavirus (COVID-19), both interviewer and interviewee will maintain safe physical distancing protocols and wear face masks. Each of the respondents will be given face masks for ensuring the protection. If any respondent refuses to wear a mask, she or he will not be interviewed.

### Frequency and plans for auditing trial conduct

Data collection performance will be checked every day by the supervisors while pretesting. Data collectors with low performance will be given an initial warning and will have to recollect the data and pre-test again. If a second incident occurs, the data collector will be terminated, and a different data collector will be assigned and trained to collect data.

Data collection supervisors will be responsible for checking data discrepancies on a daily basis and taking necessary actions. All filled questionnaires will be cross-checked by the data collection supervisors at the end of the day at field for internal validation. This will involve collecting data at the same time as the data collector and checking for discrepancies holding the supervisor’s data as standard. 20% of the data collected on any given day will undergo the agreement test. Data collectors will not be informed beforehand which household the test will be performed at. In case of any missing or inconsistent data, the respective households will be revisited by the field coordinator. Confidentiality will be maintained throughout the interview and analysis process, and de-identified data will be stored under password protection.

### Plans for communicating important protocol amendments to relevant parties (e.g., trial participants, ethical committees)

If there are important protocol modifications (e.g., changes to eligibility criteria, outcomes, analyses) the principal investigator and study team steering committee will disseminate the information to all other investigators, IRBs, trial participants, and trial registries as soon as possible using electronic communication media (secure e-mail).

### Dissemination plans

During the planning phase of the study, our group held a very successful stakeholder workshop on January 16, 2023, in Parbatipur, Bangladesh, prior to the start of enrollment. Key government officials, local influencers, and schoolteachers attended the workshop and were happy with the research we intended to conduct. Many questions were asked by these stakeholders, which helped us understand the sentiments we might receive from the study subjects. These were qualitatively incorporated into our participant education and messaging materials.

Ultimately, we plan to publish our study findings in peer-reviewed journals to address the broader scientific community.

Specifically, we plan to disseminate our research findings to the following entities:

1. The Bangladesh Ministry of Health through a policy brief.
2. National seminars through presentations of research abstracts in Bangladesh.
3. International workshops and conferences through presentations of research abstracts (e.g., American Heart Association Scientific Sessions; American Heart Association Council on Epidemiology and Lifestyles).
4. Stanford University King Center on Development as project deliverable report.

As for academic outcomes, we believe our study will result in at least two peer-reviewed publications, with one describing our study protocol and one describing the results of our clinical trial.

## Discussion

High blood pressure is a global epidemic which is rapidly increasing in prevalence and severity in LMICs, particularly in South Asia. Given the challenges of implementing population-level behavioral interventions in low-resource settings, innovative products requiring minimal lifestyle change such as LSSS are attractive potential tools for combatting the problem. Recent large LMIC trials have shown impressive health benefits for LSSS in Asia, with the recent Salt Substitute and Stroke Study even finding a 12% all-cause mortality rate reduction in study participants randomized to an LSSS arm [18, 19]. We believe that the next step in translating such discoveries into actionable global health practice involves identifying the best mechanism for dissemination of LSSS in low-resource settings. Thus, our present RCT offers the opportunity to examine whether community health worker-based provision of low-sodium salt is sufficient to lead to a measurable decrease in blood pressure in a rural, community-dwelling adult population. CHWs are a powerful, respected force multiplier in public health, and are already integrated into many of the LMIC communities where LSSS would be most efficacious [20]. Thus, they represent an appealing potential personnel arm of the healthcare system to deliver LSSS interventions.

As for limitation of our study, the cluster randomization enrollment method risks introducing residual confounding, as individuals within a cluster are often more like each other than they are to participants in other clusters. This is a typical study design in practical global health interventions, however, and we will leverage the expertise of our collaboration at BRAC JPGSPH to capture and account for these variables by adjusting our final regression model for anticipated covariates. Additionally, although the intervention will not be blinded to the participants (as the study design will not allow this) we believe that this will actually allow us to capture the causal treatment effect of participants being provided information and the opportunity to purchase low-sodium salt on the open market (rather than being provided it free of charge), which is more reflective of real-life situations where our intervention may be encountered by our target population.

## Trial status

Protocol Version 7 (January, 2023). Recruitment is planned to be completed by approximately end of February, 2023.

## Supplementary Information

**Additional file 1: Supplementary material 1.** Flipchart on Noncommunicable Diseases.

**Additional file 2.**

**Additional file 3: Supplementary material 3.** LSSS Intervention Study: Baseline Survey Questionnaire.

**Additional file 4.**

**Additional file 5: Supplementary material 5. **Low-Sodium Salt Substitute Intervention: Consent Form.

## Data Availability

The final trial dataset will only be available to the BRAC site primary investigator (author MS) and co-investigators (authors MR, AT, and MH). Authors PG and AYC will only be allowed to access a deidentified, coded version of the dataset per the stipulations of the Stanford IRB protocol during the study period. Following publication of the primary analysis, a de-identified version of the dataset, along with the statistical code will be publicly available via publication in the Stanford Digital Repository.

## Abbreviations

LSSS: Low-sodium salt substitute
LMICs: Low- and middle-income countries
BP: Blood pressure
BRAC JPGSPH: BRAC James P Grant School of Public Health
IRB: Institutional Review Board
NaCl: Sodium chloride
KCl: Potassium chloride
MgSO_4_: Magnesium sulfate
RCT: Randomized controlled trial
ICC: Intra-cluster coefficient
MI: Myocardial infarction
OR: Odds ratio
CKD: Chronic kidney disease
GRT: Group- or cluster-randomized trials
CHW: Community health worker
SBP: Systolic blood pressure
DBP: Diastolic blood pressure
COVID-19: Coronavirus disease 2019
