# Supplementary material for: Effectiveness of a community health worker-led low-sodium salt intervention to reduce blood pressure in rural Bangladesh: protocol for a cluster randomized controlled trial
Source: Trials. 2023 Jul 27;24:480. doi: 10.1186/s13063-023-07518-3 (PMC10375753; doi:10.1186/s13063-023-07518-3)
Supplement: Supplementary file 1 — Additional file 1: Supplementary material 1. Flipchart on Noncommunicable Diseases. [file 13063_2023_7518_MOESM1_ESM.pdf]

অসংক্রামক রোগ বিষয়ক তথ্য সহায়িকা

(Flip chart on Noncommunicable Diseases)

কমিউনিটি স্বাস্থ্যকর্মী/স্বেচ্ছাসেবীদের ব্যবহারের জন্য

(For the use of Community Health Workers/Volunteers)

World Health Organization Bangladesh

Community Health Working Group

From the People of Japan

BRAC JAMES P GRANT SCHOOL OF PUBLIC HEALTH

BRAC UNIVERSITY Inspiring Excellence

পরিকল্পনা, প্রণয়ন ও প্রকাশনা

বিশ্ব স্বাস্থ্য সংস্থা
কমিউনিটি হেলথ ওয়ার্কিং গ্রুপ, কক্সবাজার
ব্র্যাক জেমস পি গ্রান্ট স্কুল অফ পাবলিক হেল্‌থ, ব্র্যাক বিশ্ববিদ্যালয়

প্রকাশকাল

নভেম্বর ২০১৯

ছবির উৎস

ফ্রি ক্লিপ আর্ট, যথাযথ অনুমতি নিয়ে তোলা ছবি

World Health Organization Bangladesh
Community Health Working Group
From the People of Japan
BRAC JAMES P GRANT SCHOOL OF PUBLIC HEALTH
BRAC UNIVERSITY Inspiring Excellence

অসংক্রামক রোগের প্রভাব

* শারীরিক অসুস্থতা
* মানসিক অসুস্থতা
* কর্মক্ষমতা হ্রাস
* স্বল্পমেয়াদী ও দীর্ঘমেয়াদী অক্ষমতা
* অকাল মৃত্যু

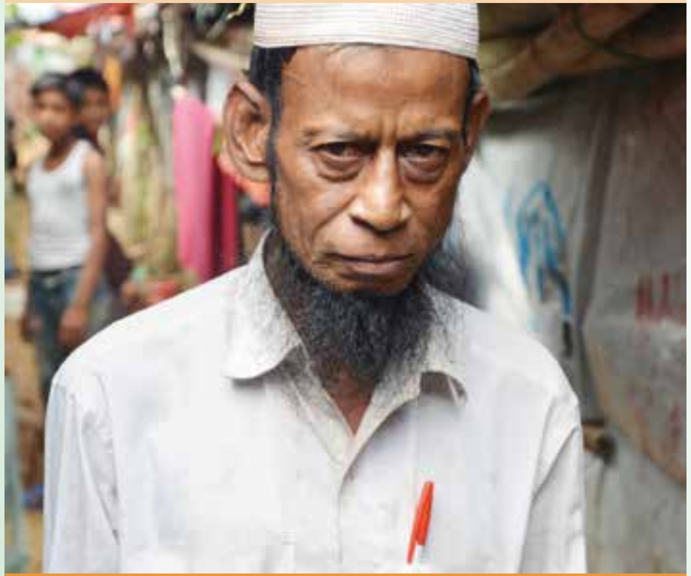

ব্যক্তির উপর প্রভাব

* আয় কমে যাওয়া
* চিকিৎসার খরচ বেড়ে যাওয়া
* দারিদ্র্য
* মৌলিক চাহিদা পূরণ না হওয়া
* পারিবারিক সহিংসতা

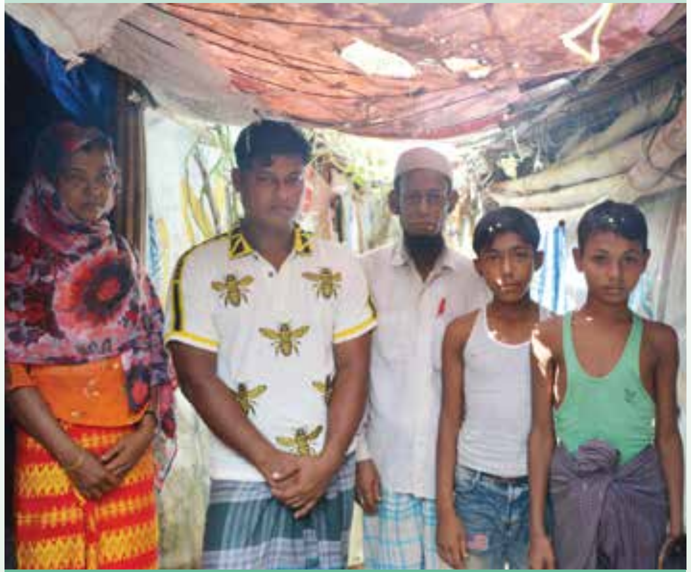

পরিবারের উপর প্রভাব

* কর্মক্ষম মানুষের সংখ্যা হ্রাস
* নির্ভরশীল মানুষের সংখ্যা বৃদ্ধি
* অসাম্যতা
* সহিংসতা ও অস্থিরতা

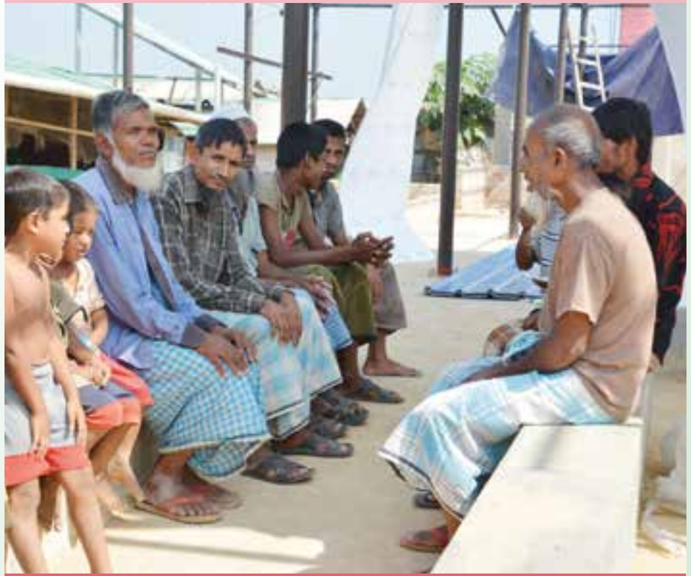

সমাজের উপর প্রভাব

অসংক্রামক রোগের প্রভাব

অসংক্রামক রোগের কারণে ব্যক্তি, পরিবার, সমাজ, সর্বোপরি দেশ ক্ষতিগ্রস্ত হয়

ব্যক্তির উপর প্রভাব

অসংক্রামক রোগের কারনে শারীরিক ও মানসিক অসুস্থতা হয়।
এর ফলে মানুষ শারীরিক বা মানসিকভাবে অক্ষম হয়ে যেতে পারে এবং মানুষের কর্মক্ষমতা কমে যায়।
অসংক্রামক রোগের কারনে মানুষের অকাল মৃত্যু ঘটে।

পরিবারের উপর প্রভাব

অসংক্রামক রোগের কারনে পরিবার ক্ষতিগ্রস্ত হয়।
পরিবারের আয় কমে যায়, চিকিৎসার খরচ বেড়ে যায়।
পরিবারের সদস্যদের মৌলিক চাহিদা পূরণ হয় না।
পরিবার দরিদ্র হয়ে পরে এবং পারিবারিক সহিংসতা বাড়ে।

সমাজের উপর প্রভাব

অসংক্রামক রোগের ফলে সমাজের উপর ক্ষতিকর প্রভাব পড়ে।
সমাজে কর্মক্ষম মানুষের সংখ্যা কমে যায় এবং নির্ভরশীল মানুষের সংখ্যা বৃদ্ধি পায়।
সমাজে অসাম্যতা বৃদ্ধি পায় এবং সমাজে সহিংসতা ও অস্থিরতা সৃষ্টি হয়।

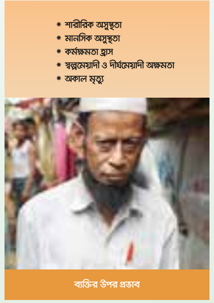

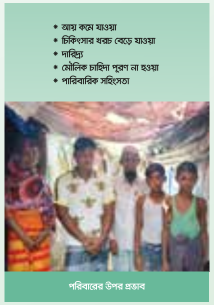

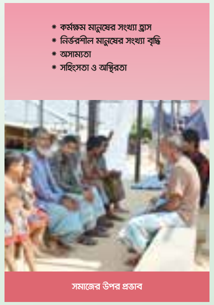

FOR CHW

অসংক্রামক রোগের ঝুঁকি

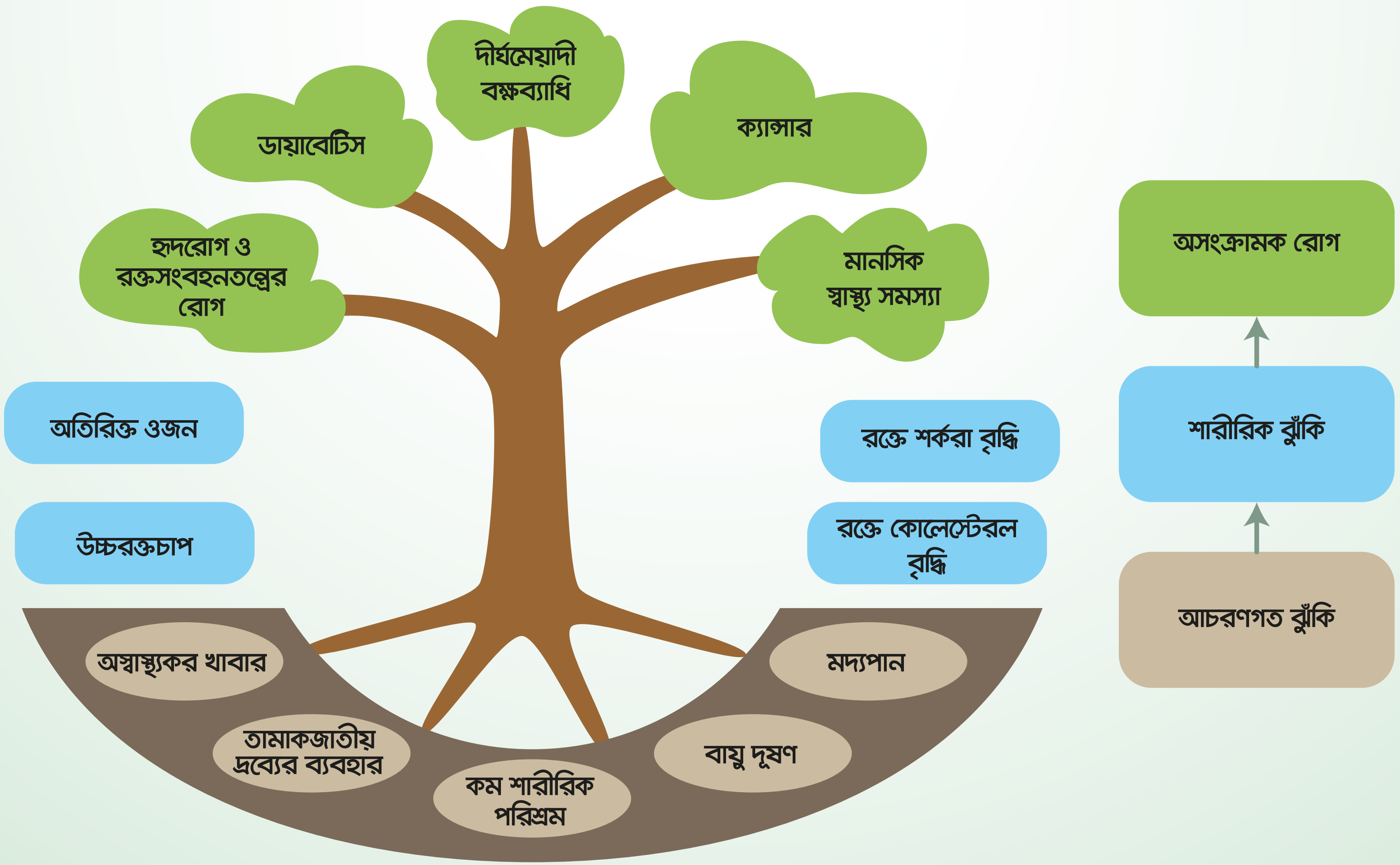

FOR COMMUNITY

অসংক্রামক রোগের ঝুঁকি

অসংক্রামক রোগের কতগুলো আচরণগত ও শারীরিক ঝুঁকি রয়েছে। চেষ্টা করলে এই ঝুঁকিগুলো কমানো যায়।

আচরণগত ঝুঁকিঃ

অস্বাস্থ্যকর খাবারঃ বেশি পরিমাণে লবন, চিনি, চর্বিযুক্ত খাবারে অসংক্রামক রোগ বাড়ে।
তামাকজাতীয় দ্রব্যের ব্যবহারঃ তামাকজাতীয় দ্রব্য যেমন বিড়ি, সিগারেট, জর্দা, সাদাপাতা খেলে ক্যান্সার ছাড়াও অন্যান্য অসংক্রামক রোগে আক্রান্ত হবার সম্ভাবনা বেড়ে যায়।
কম শারীরিক পরিশ্রমঃ কম শারীরিক পরিশ্রম করলে অসংক্রামক রোগ বাড়ে।
মদ্যপানঃ মদ্যপান করলেও অসংক্রামক রোগে আক্রান্ত হবার সম্ভাবনা বেড়ে যায়।
বায়ুদূষণঃ বায়ুদূষণের সাথেও অসংক্রামক রোগের সম্পর্ক আছে।

শারীরিক ঝুঁকিঃ

অতিরিক্ত ওজনঃ অতিরিক্ত শারীরিক ওজন থাকলে অসংক্রামক রোগে আক্রান্ত হবার সম্ভাবনা বেড়ে যায়।
উচ্চরক্তচাপঃ উচ্চরক্তচাপ হৃদপিণ্ড, রক্তনালী, মস্তিষ্ক এবং কিডনির উপর খারাপ প্রভাব ফেলে।
রক্তে শর্করা বৃদ্ধিঃ রক্তে শর্করার পরিমান বৃদ্ধি পেলে ডায়াবেটিস হয়। যার ফলে অন্যান্য অসংক্রামক রোগে আক্রান্ত হবার সম্ভাবনা বেড়ে যায়।
রক্তে কোলেস্টেরল বৃদ্ধিঃ রক্তে অতিরিক্ত পরিমান কোলেস্টেরল রক্তনালীতে রক্ত প্রবাহে বাঁধা তৈরি করে। যা পরে হার্ট অ্যাটাক বা স্ট্রোকের ঝুঁকি বাড়িয়ে দেয়।

অসংক্রামক রোগের আরো কতগুলো ঝুঁকি রয়েছে। যেগুলো পরিবর্তন করা যায় না।

অপরিবর্তন যোগ্য ঝুঁকিঃ

বয়সঃ অসংক্রামক রোগ বয়সের সাথে সম্পর্কিত। বয়স বাড়লে অসংক্রামক রোগের সম্ভাবনা বাড়ে।
লিঙ্গঃ পাকস্থলীতে ক্যান্সার মহিলাদের চেয়ে পুরুষদের বেশি হয়। কোন কোন অসংক্রামক রোগ পুরুষদের বেশি হয়। তেমনি কোন কোন অসংক্রামক রোগ মহিলাদের বেশি হতে পারে।
বংশগতির ধারাঃ পরিবারে কারো অসংক্রামক রোগ থাকলে, তাদের ছেলে-মেয়েরও অসংক্রামক রোগ হতে পারে।
জাতিগোষ্ঠীঃ জাতিভেদে কিছু কিছু অসংক্রামক রোগ বেশি হয়।

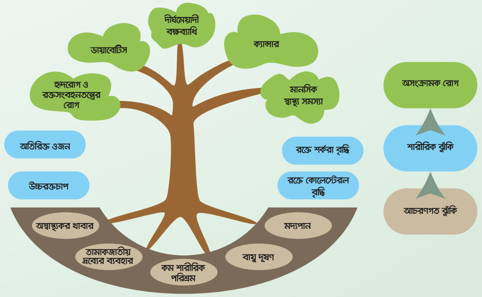

তথ্যসূত্র: আমেরিকান ক্যান্সার সোসাইটি

FOR CHW

স্বাস্থ্যকর খাবার

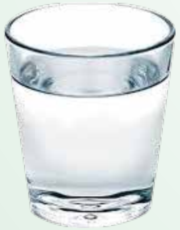

নিরাপদ পানি

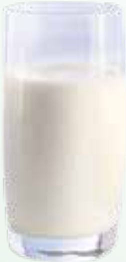

দুগ্ধ জাতীয় খাবার

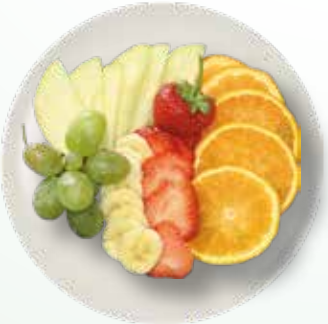

ফল

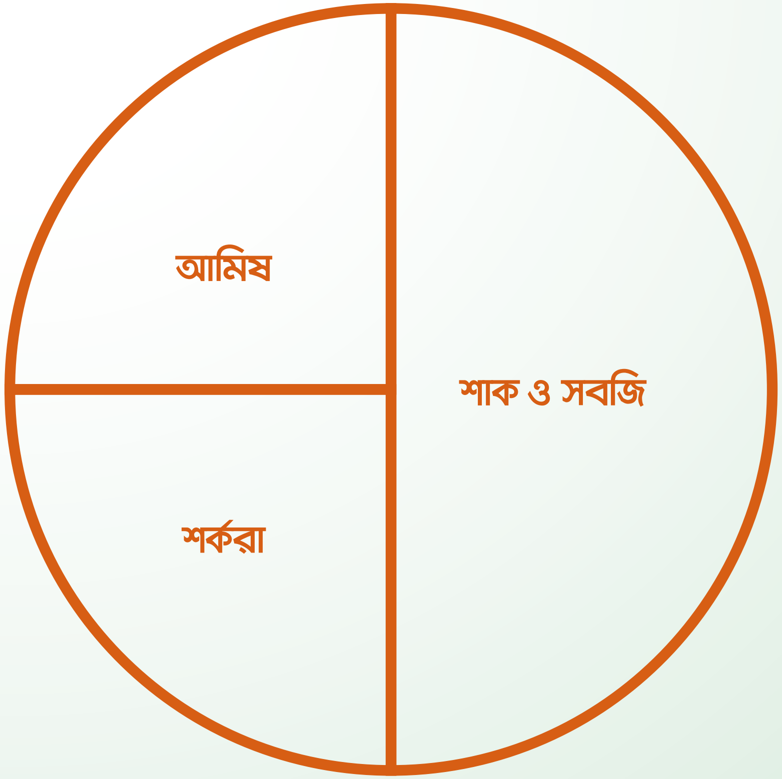

খাবারের থালায় বিভিন্ন ধরণের খাদ্যের অনুপাত

FOR COMMUNITY

স্বাস্থ্যকর খাবার

ছবিতে একটি থালার মধ্যে দুপুর বা রাতের খাবারে বিভিন্ন ধরণের খাদ্যের অনুপাত দেখানো হয়েছে। ছবিতে দেখা যাচ্ছে খাবারের থালার চার ভাগের এক ভাগ শর্করা জাতীয় খাবার, এক ভাগ আমিষ জাতীয় খাবার, ও দুইভাগ শাক সবজি হতে হবে।

শর্করা জাতীয় খাবারের মধ্যে রয়েছে ভাত, রুটি, পাউরুটি, আলু ইত্যাদি।

আমিষ জাতীয় খাবারের মধ্যে রয়েছে মাছ, মাংস, ডিম, ডাল, শিমের বীজ ইত্যাদি।

বিভিন্ন ধরণের শাক ও সবজি এবং নানা ধরণের ফলমূল নির্দিষ্ট অনুপাতে খেতে হবে। শাকের মধ্যে রয়েছে সবুজ শাক, পালং শাক, পুই শাক, লাল শাক ইত্যাদি। সবজির মধ্যে রয়েছে মিষ্টি কুমড়া, লাউ, কাঁচা পেঁপে ইত্যাদি।

এছাড়াও ফলমূল, দুধ ও দুগ্ধজাতীয় খাবার খাওয়া প্রয়োজন।

আমাদের শরীরের তিন ভাগের দুই ভাগ পানি থাকে। তাই সারাদিনে ২-২.৫ লিটার পানি খাওয়া প্রয়োজন।

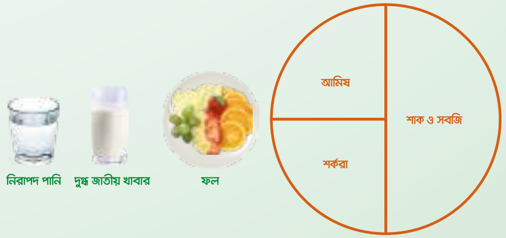

খাবারের থালায় বিভিন্ন ধরণের খাদ্যের অনুপাত

FOR CHW

বিভিন্ন রংয়ের ফলমূল ও শাকসবজি

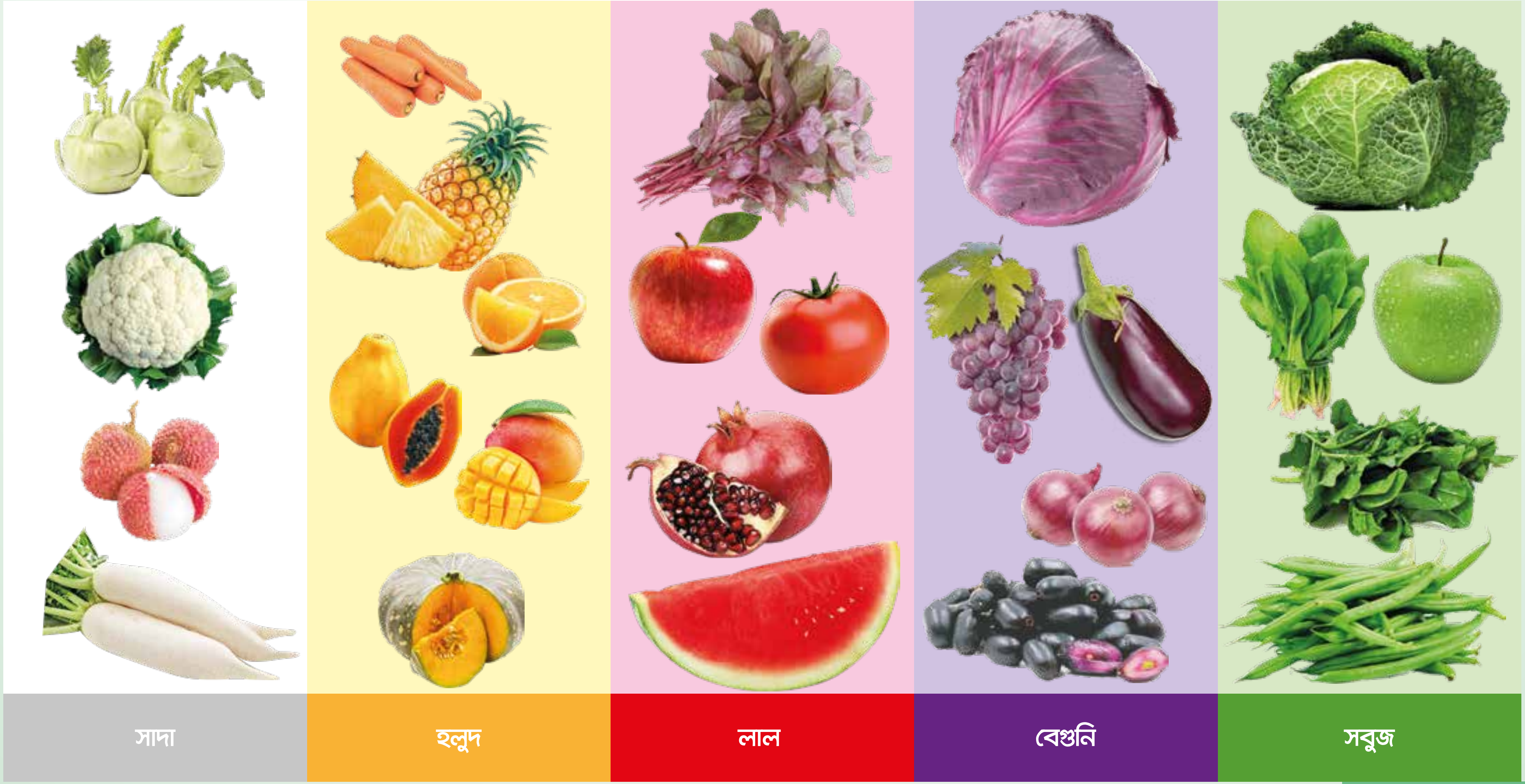

তথ্যসূত্র: জাপান ইন্টারন্যাশনাল কোঅপারেশন এজেন্সি (জাইকা)

বিভিন্ন রংয়ের ফলমূল ও শাকসবজি

শাক সবজি এবং ফলমূলের উপকার

বিভিন্ন রংয়ের শাক সবজি রয়েছে। যেগুলো শরীরের রোগ প্রতিরোধ ক্ষমতা বাড়ায় এবং সুস্বাস্থ্যের অধিকারী হতে সাহায্য করে। প্রতিদিন শাক সবজি ও ফলমূল খেলে হৃদরোগ, স্ট্রোক, ডায়াবেটিস এবং ক্যান্সারের ঝুঁকি কমে যায়।

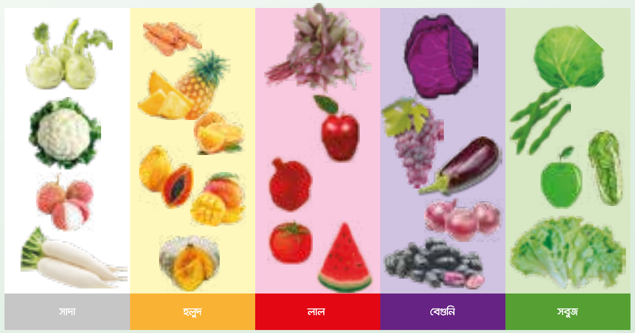

FOR CHW

দৈনন্দিন খাবারে শাকসবজি, ফলমূল এবং তার পরিমান

যা খাবেন

ফল

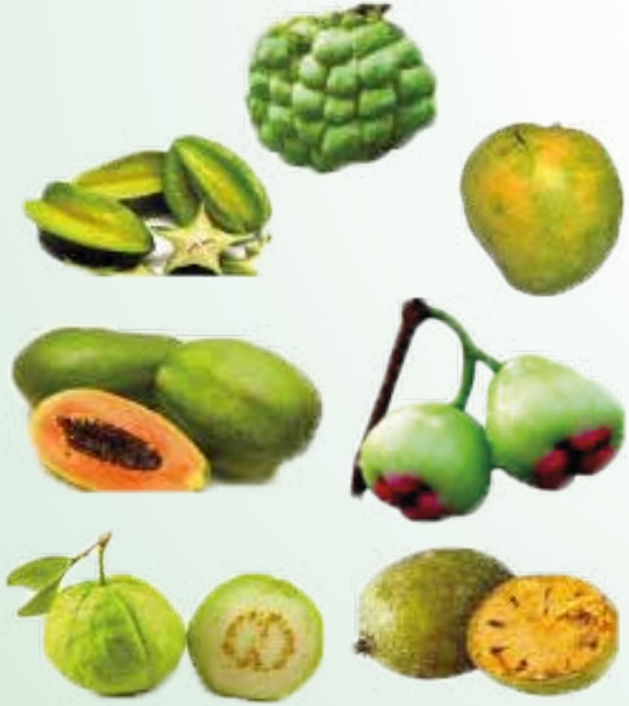

সবজি

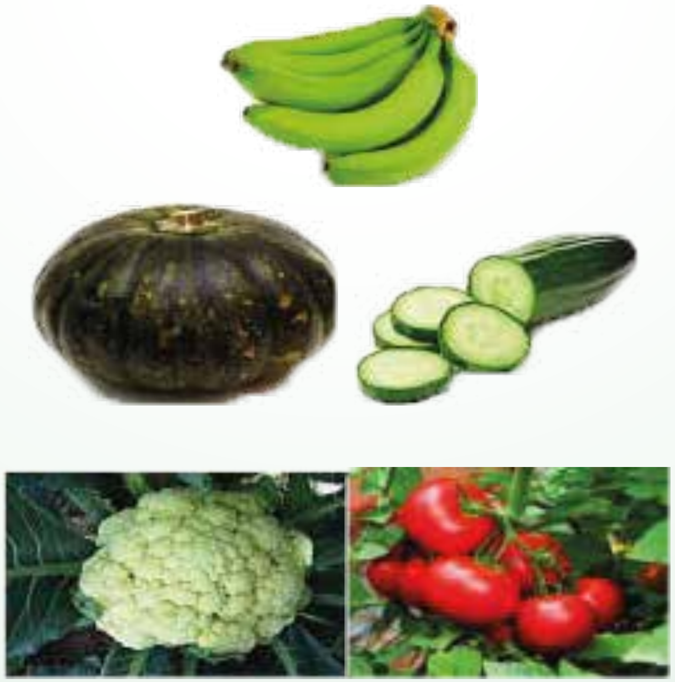

শাক

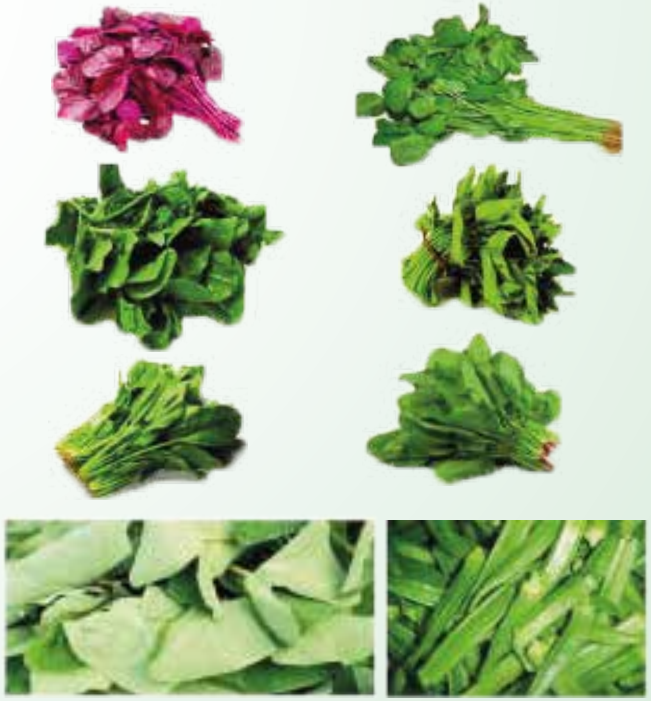

প্রতিদিন ন্যূনতম ৪০০ গ্রাম (৫ ভাগ) বিভিন্ন রংয়ের ফল ও শাক সবজি 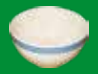 X ৫

FOR COMMUNITY

তথ্যসূত্র: জাপান ইন্টারন্যাশনাল কোঅপারেশন এজেন্সি (জাইকা)

দৈনন্দিন খাবারে শাকসবজি, ফলমূল এবং তার পরিমান

প্রতিদিন শাকসবজি এবং ফলমূল কতটুকু খাওয়া প্রয়োজন?

প্রতিদিন কমপক্ষে ৫ ভাগ (১ ভাগ= ৮০ গ্রাম) ফল ও শাকসবজি খাওয়া প্রয়োজন। আপনি বিভিন্ন ভাবে ৫ ভাগ বা ৪০০ গ্রাম শাকসবজি খেতে পারেন। যেমনঃ একবাটি রান্না করা সবজি, ২ ভাগের সমান। আপনি যদি সারাদিনে দেড়বাটি রান্না করা সবজি, দুটি কলা এবং একটি কমলা খান তাহলে আপনার ৫ ভাগ শাকসবজি ও ফলমূল খাওয়া হয়ে যাবে।

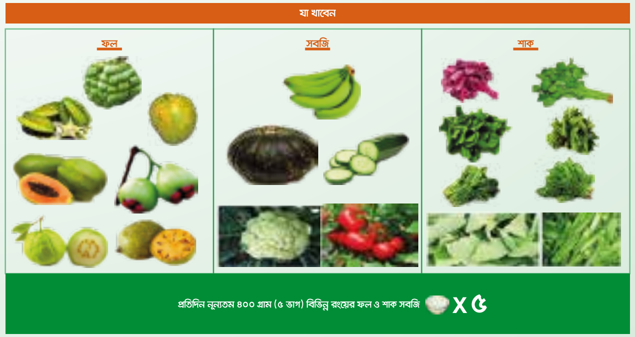

FOR CHW

দৈনন্দিন খাবারে লবণ

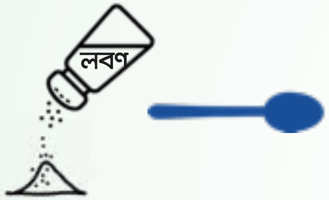

১ দিনে ১ চা চামচের বেশি লবণ নয় (সব ধরনের খাবার মিলিয়ে)। লবণ অবশ্যই আয়োডিনযুক্ত হতে হবে।

শরীরের উপর ক্ষতিকর প্রভাব

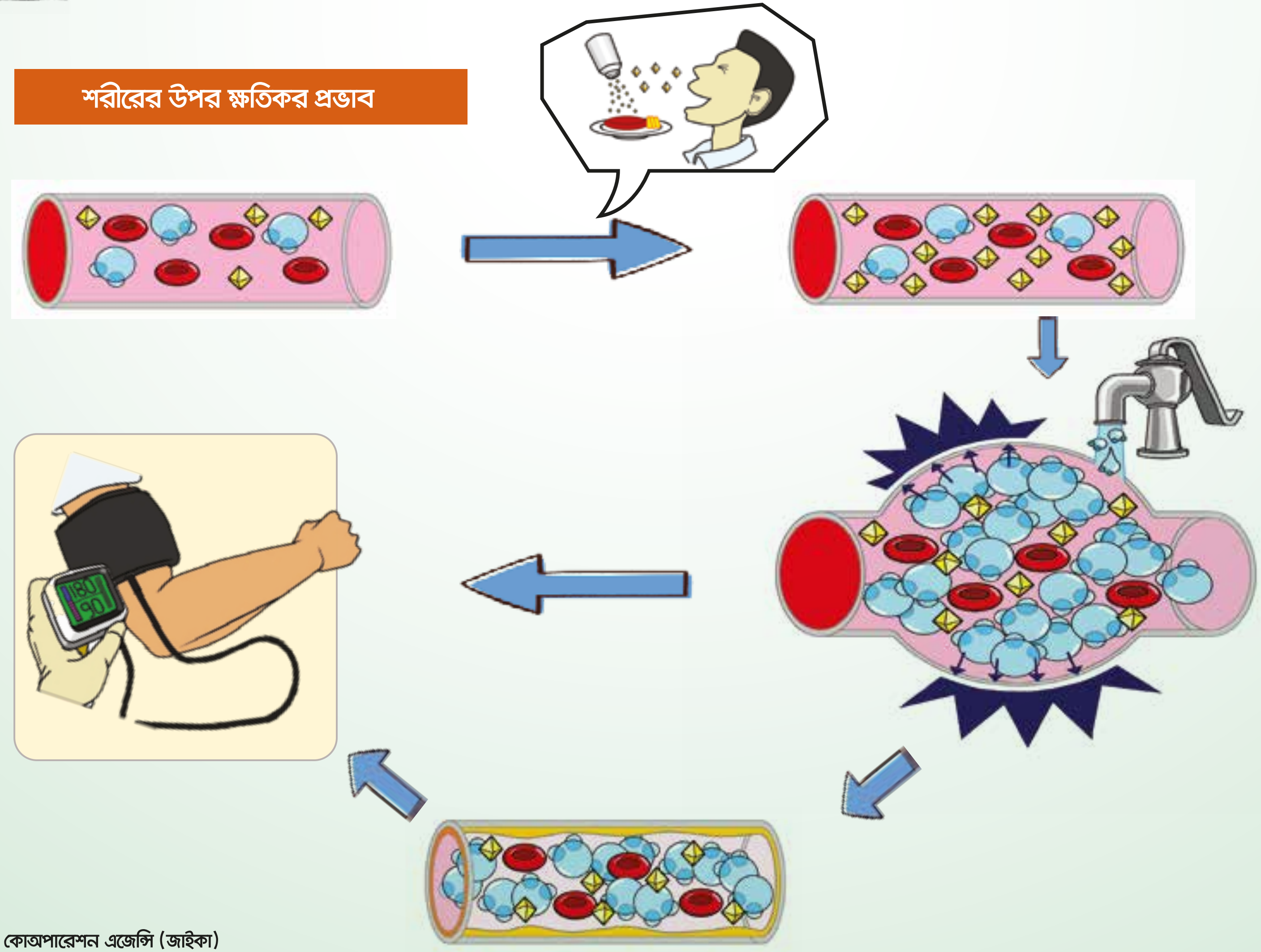

তথ্যসূত্র: জাপান ইন্টারন্যাশনাল কোঅপারেশন এজেন্সি (জাইকা)

FOR COMMUNITY

দৈনন্দিন খাবারে লবণ

খাবারে লবণ এবং এর ক্ষতিকর প্রভাব

প্রতিদিন কতটুকু লবণ খেতে পারি?

প্রতিদিন সর্বোচ্চ ৫ গ্রাম (১ চা চামচ) আয়োডিনযুক্ত লবণ খাওয়া উচিত। মনে রাখবেন, কাঁচা ও ভাজা লবনের মধ্যে কোন তফাৎ নেই। বিট লবণও শরীরের জন্য ক্ষতিকর।

শরীরের উপর লবণের ক্ষতিকর প্রভাব

লবণ আপনার শরীরে পানি ধরে রাখে। আপনি যদি বেশি লবণ খান, আপনার শরীরে জমা অতিরিক্ত পানি রক্তনালীতে ঢুকে গিয়ে রক্তচাপ বাড়িয়ে দেয়। যা স্ট্রোক, হৃদরোগ ও কিডনি রোগের ঝুঁকি বাড়িয়ে দেয়।

লবণ খাওয়া কমানোর উপায় কী ?

* পাতে লবণ একেবারে খাবেন না।
* রান্নার সময় কম লবণ ব্যবহার করুন।
* চিপস, বিট লবণ, নোনা ইলিশ, শুঁটকি, ফাস্ট ফুড সহ অন্যান্য লবণ যুক্ত খাবার পরিহার করুন।

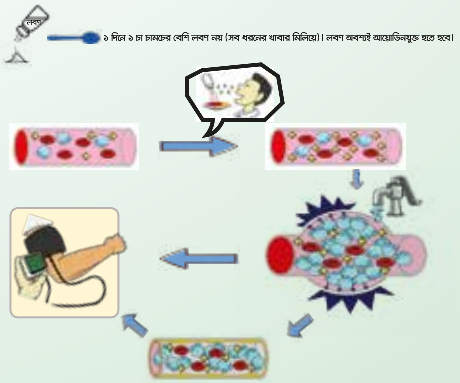

FOR CHW

দৈনন্দিন খাবারে চিনি

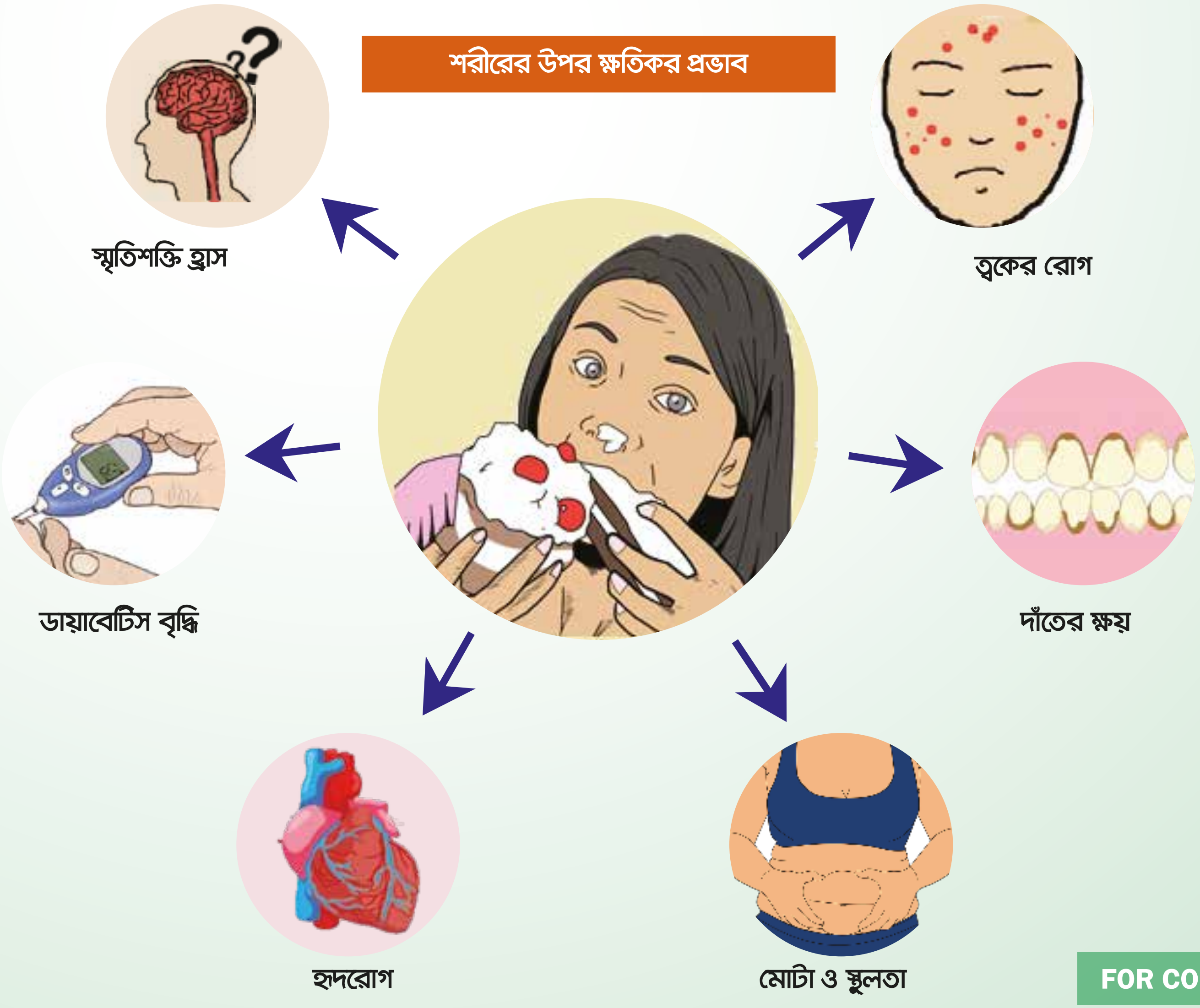

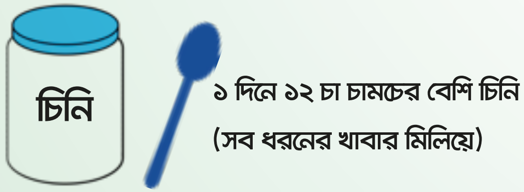

১ দিনে ১২ চা চামচের বেশি চিনি নয়
(সব ধরনের খাবার মিলিয়ে)

FOR COMMUNITY

তথ্যসূত্র: জাপান ইন্টারন্যাশনাল কোঅপারেশন এজেন্সি (জাইকা)

দৈনন্দিন খাবারে চিনি

প্রতিদিন কতটুকু চিনি খেতে পারি?

বয়স্করা দৈনিক সর্বোচ্চ ১২ চা চামচ চিনি খেতে পারবেন।

শরীরের উপর চিনির ক্ষতিকর প্রভাব

চিনি প্রধানত খাবার বা পানীয় মিষ্টি করার কাজে ব্যবহার হয়। অধিক চিনি শরীরের স্থূলতা বাড়িয়ে দেয়। এর ফলে হৃদরোগ এবং ডায়াবেটিসের ঝুঁকি সহ স্মৃতিশক্তি হ্রাস, চর্মরোগ, দাঁতের ক্ষয় ও যকৃতের সমস্যা দেখা দিতে পারে।

চিনি খাওয়া কমানোর উপায় কী ?

* প্রক্রিয়াজাত খাদ্য (চিপস, সস, ফলের জুস), ফাস্ট ফুড, কোমল পানীয় ও এনার্জি ড্রিঙ্কসের মধ্যে লুকানো চিনি থাকে। এসব খাবার পরিহার করুন।
* কোমল পানীয়ের পরিবর্তে পানি পান করুন।
* মিষ্টি খাবারের পরিবর্তে টাটকা ফল খান।
* চা বা কফিতে চিনি ব্যবহার বাদ দিয়ে দিন বা কমিয়ে ফেলুন।

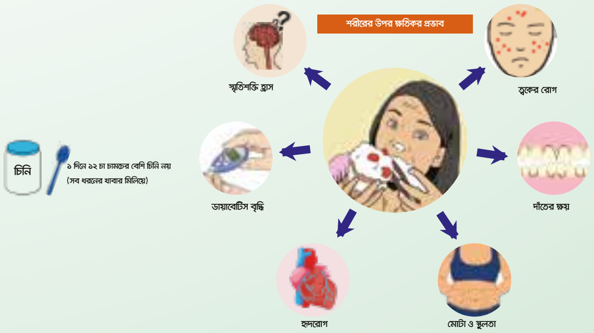

FOR CHW

দৈনন্দিন খাবারে তেল ও চর্বি

পরিমিত পরিমাণ তেল ও চর্বি

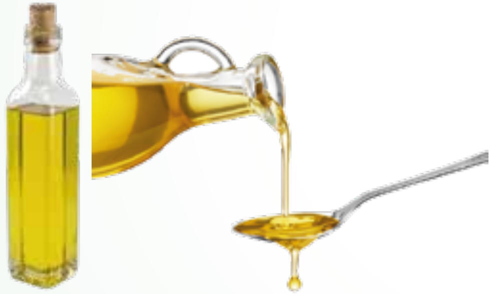

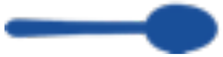

প্রতিদিন পরিমিত পরিমাণ স্বাস্থ্যসম্মত তেল ও চর্বি খান

স্বাস্থ্যসম্মত তেল ও চর্বি গ্রহন

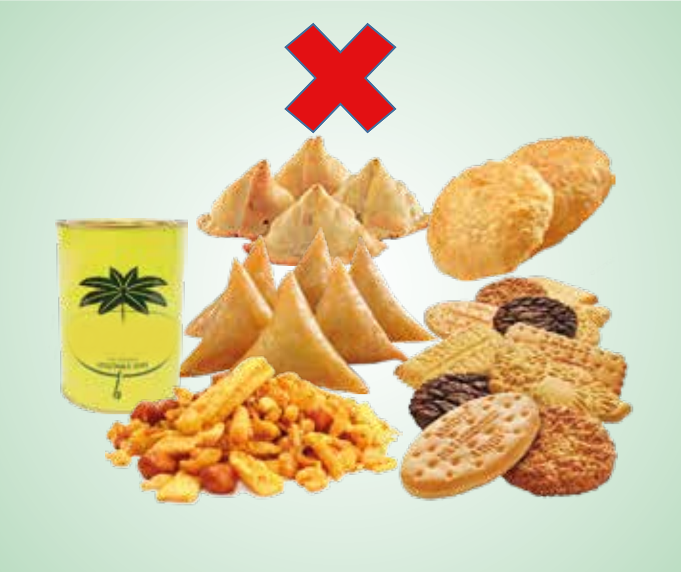

পরিবর্তিত চর্বি

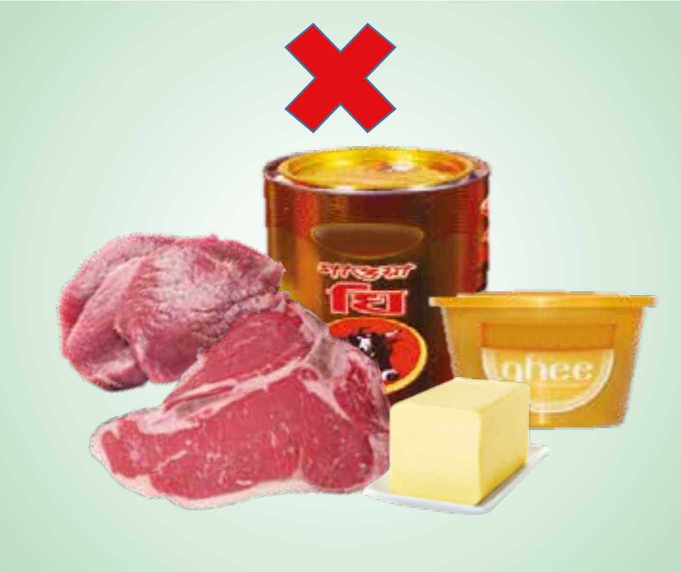

অন্যান্য প্রানি থেকে প্রস্তুত তেল ও চর্বি

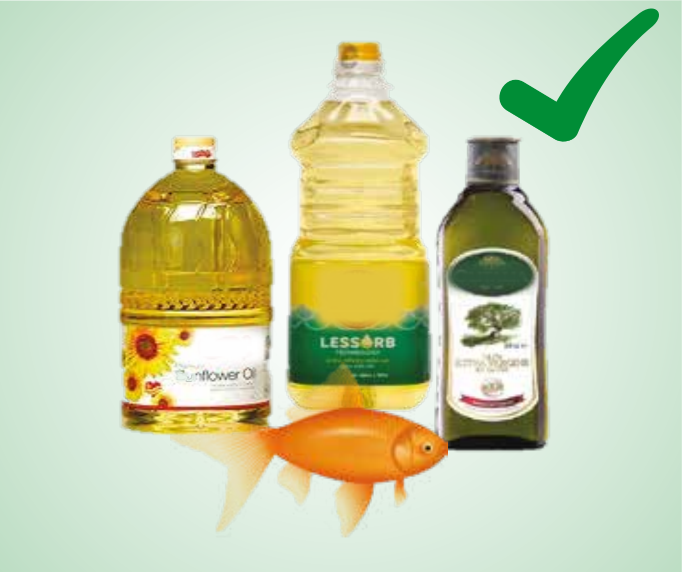

উদ্ভিদ থেকে প্রস্তুত তেল ও চর্বি এবং মাছের তেল

FOR COMMUNITY

দৈনন্দিন খাবারে তেল ও চর্বি

প্রতিদিন কতটুকু তেল ও চর্বি খেতে পারেন?

প্রতিদিন পরিমিত পরিমাণ স্বাস্থ্যসম্মত তেল ও চর্বি খেতে পারেন।

শরীরের উপর তেল ও চর্বির ক্ষতিকর প্রভাব

প্রানি থেকে উৎপন্ন সম্পৃক্ত চর্বি ও পরিবর্তিত চর্বি (যেমন ডালডা, বনস্পতি ঘি) আপনার রক্তে কোলেস্টেরলের মাত্রা বাড়িয়ে দেয়। রক্তে অধিক কোলেস্টেরল ধমনী সরু করে দেয় এবং এর ফলে হৃদরোগ ও স্ট্রোকের ঝুঁকি বেড়ে যায়।

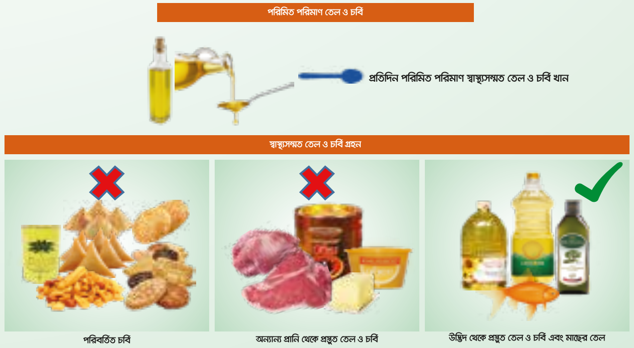

FOR CHW

দৈনন্দিন খাবারে তেল ও চর্বি কমানোর উপায়

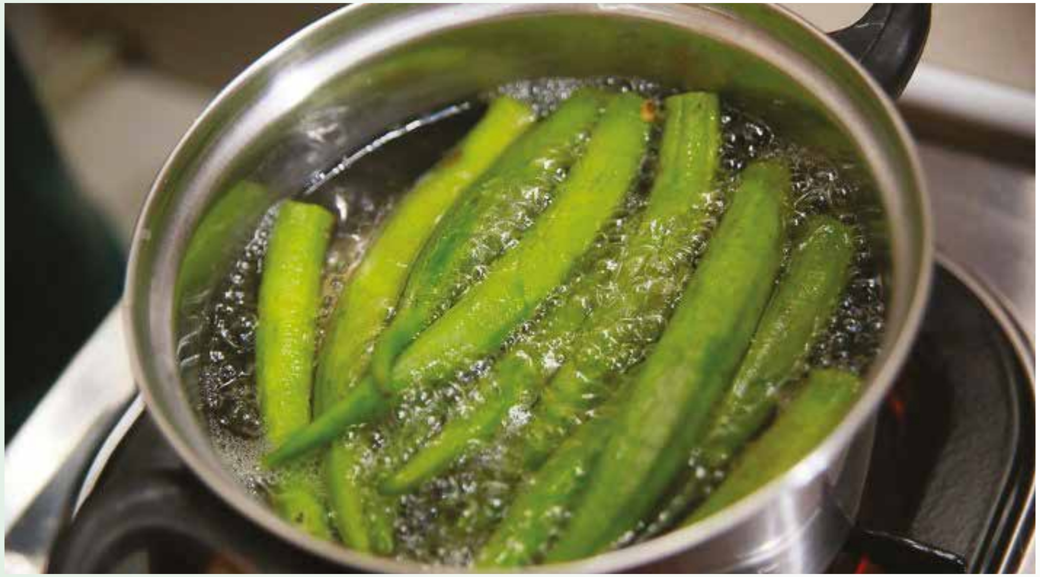

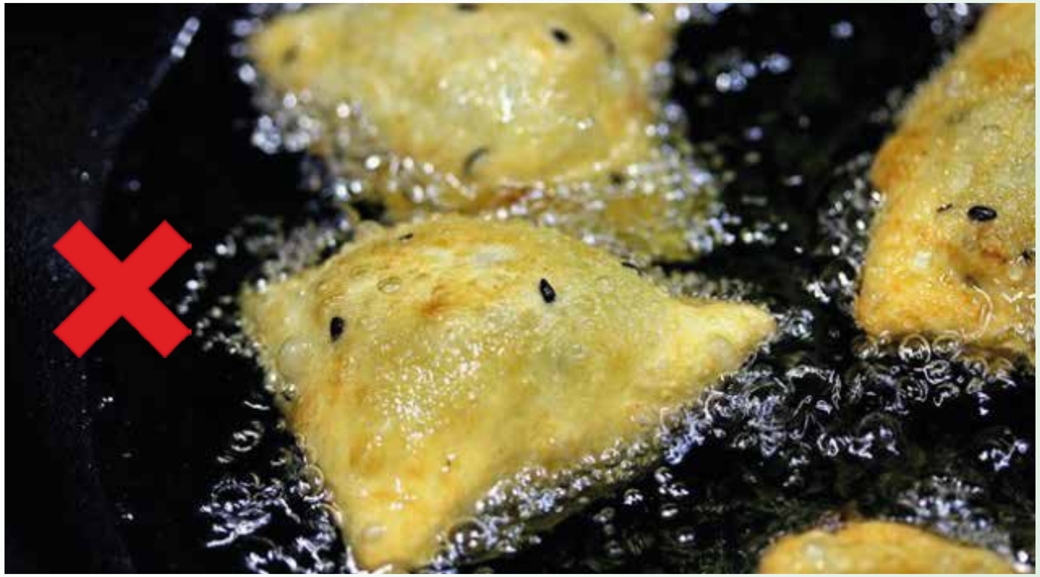

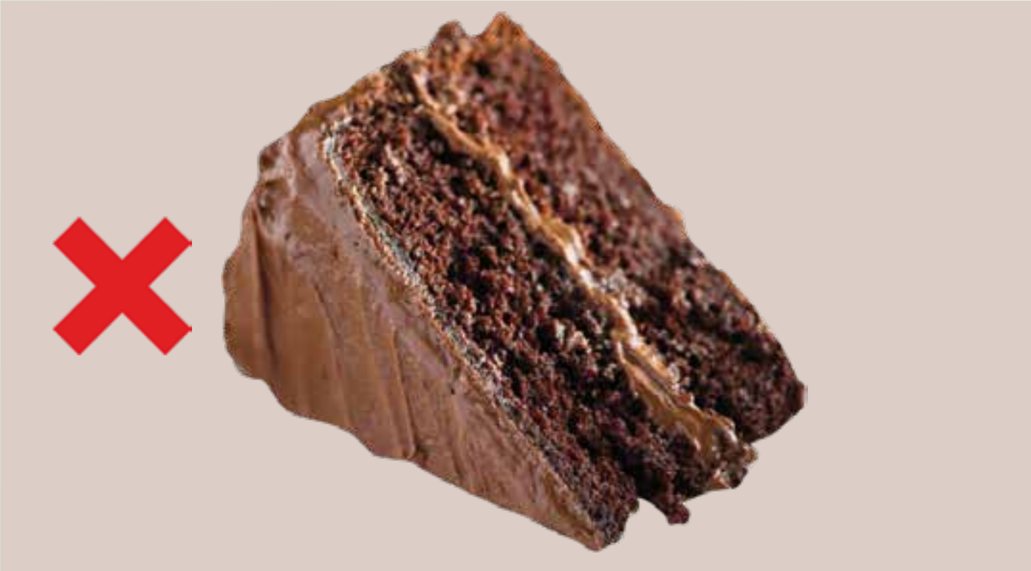

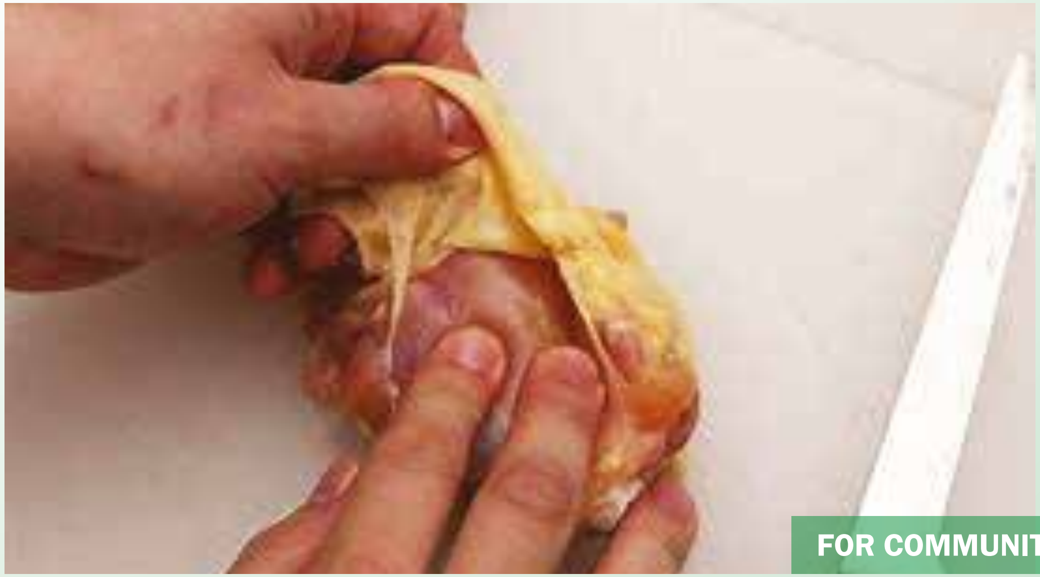

FOR COMMUNITY

দৈনন্দিন খাবারে তেল ও চর্বি কমানোর উপায়

কমানোর উপায় কী কী?

* স্বাস্থ্যকর রান্নার তেল ব্যবহার করুন।
* চর্বি ছাড়া মাংস খেতে পারেন। হাঁস ও মুরগীর চামড়া খাওয়া যাবেনা।
* খাদ্য তৈরির ক্ষেত্রে ভাজার চেয়ে সিদ্ধ করে বা ভাপে সিদ্ধ করে খান।
* উচ্চ সম্পৃক্ত চর্বিযুক্ত খাবার যেমনঃ আইসক্রিম, কেক খাদ্য তালিকা থেকে বাদ দিন।
* লাল মাংস বাদ দিয়ে সাদা মাংস খান।
* হাঁস, মুরগী, মাংসের পরিবর্তে মাছ খান।
* ডালডা, বনস্পতি ঘি দিয়ে তৈরি খাবার পরিহার করুন।

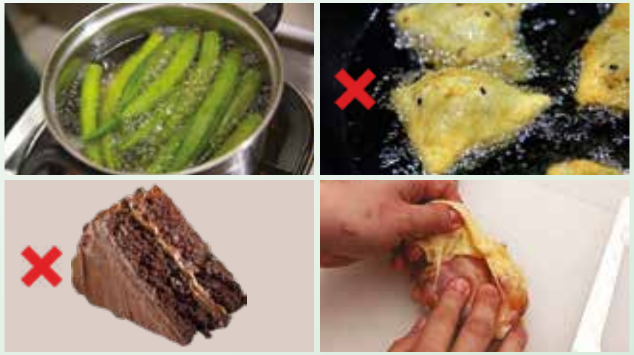

FOR CHW

অস্বাস্থ্যকর, প্রক্রিয়াজাত এবং তেলচর্বিযুক্ত খাবার

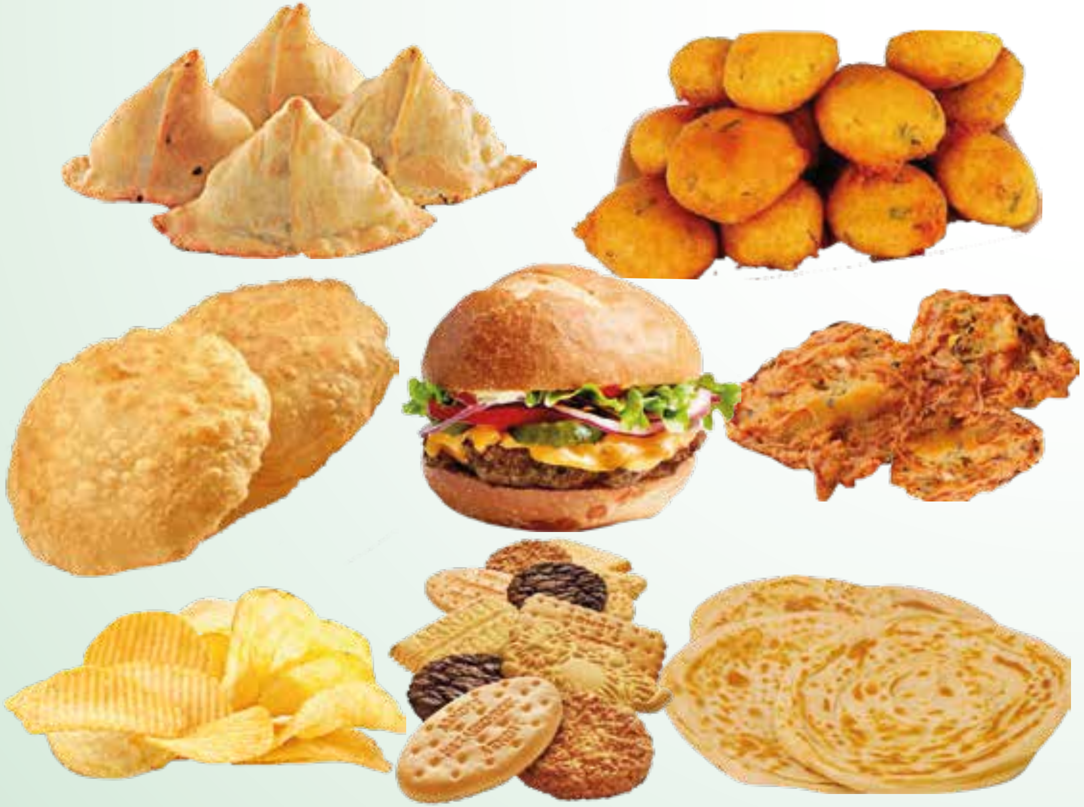

স্বাস্থ্যকর তেল ও চর্বিজাতীয় খাবার

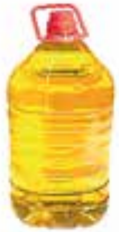
সয়াবিন তেল

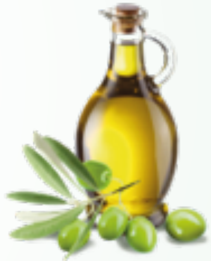
জলপাইয়ের তেল

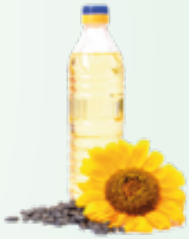
সূর্যমুখীর তেল

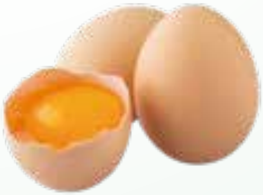
ডিম

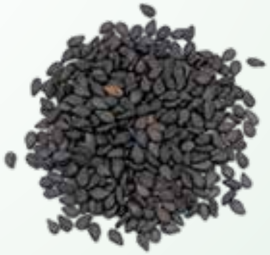
তিল

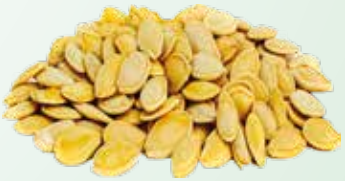
মিষ্টি কুমড়ার বীজ

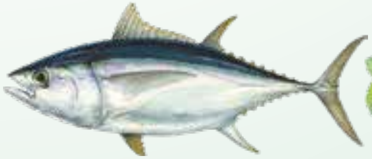
মাছের তেল

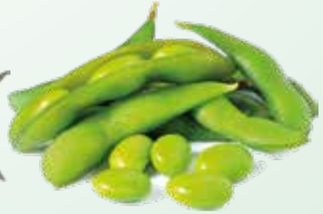
সয়াবিন

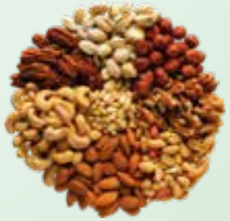
বিভিন্ন ধরণের বাদাম

FOR COMMUNITY

কেন প্রক্রিয়াজাত খাবার পরিহার করা উচিত

* প্রক্রিয়াজাত খাবারে প্রচুর পরিমানে লবন, চিনি এবং চর্বি থাকে।
* প্রক্রিয়াজাত খাবারে পচনরোধী রাসায়নিক দ্রব্য থাকে।

অস্বাস্থ্যকর তেল ও চর্বি কেন খাবেন না

* অস্বাস্থ্যকর চর্বির (সম্পৃক্ত) চেয়ে স্বাস্থ্যকর চর্বি (অসম্পৃক্ত) তুলনামূলক ভাবে ভাল। সূর্যমুখী তেল, জলপাইয়ের তেল, সয়াবিন তেল, ডিম, তিসি, মাছের তেল, সয়াবিন, বিভিন্ন ধরণের বাদাম ইত্যাদি থেকে স্বাস্থ্যকর তেল বা চবি পাওয়া যায়।
* অতিরিক্ত চর্বিযুক্ত খাবার খেলে, অল্প খাবারে অধিক শক্তি পাওয়া যায়। এই অতিরিক্ত শক্তি পরবর্তীতে দেহে জমা হয়। এর ফলে ওজন বেড়ে যায়।
* যদি আপনি “চর্বি খাওয়া খারাপ” বলে মনে করেন এবং এর পরিবর্তে আরও বেশি করে শর্করা খান তবেও আপনার ওজন বাড়বে এবং রক্তের কোলেস্টেরলের মাত্রাও বাড়বে।

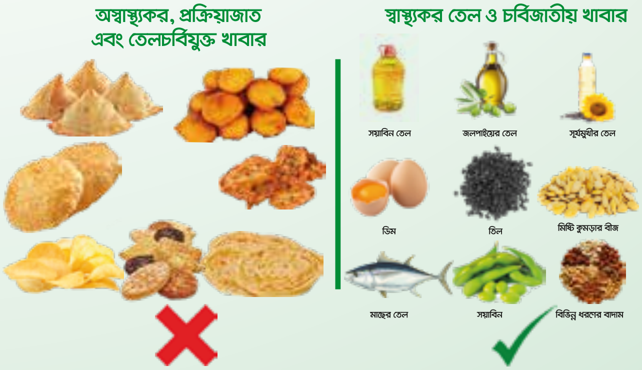

FOR CHW

বিভিন্ন ধরণের তামাকজাত দ্রব্য

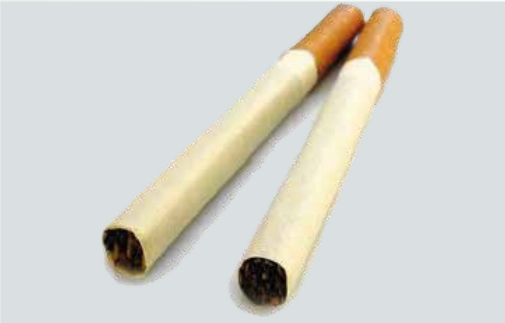

সিগারেট

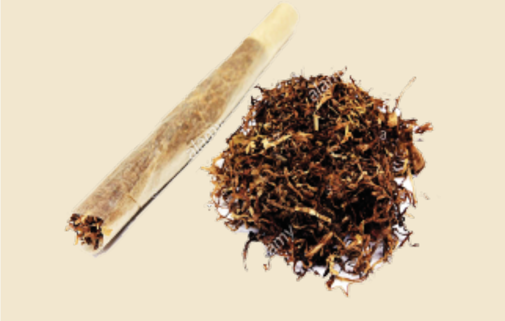

হাতে তৈরি সিগারেট

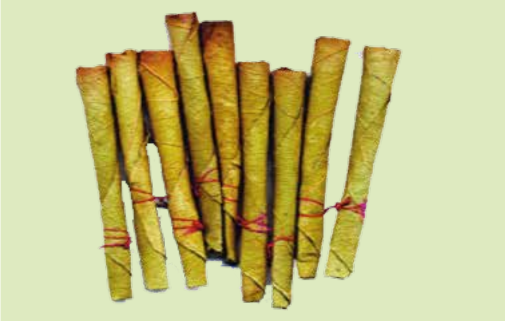

বিড়ি

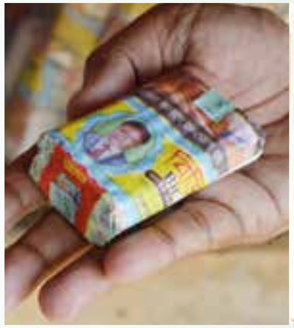

বিড়ির প্যাকেট

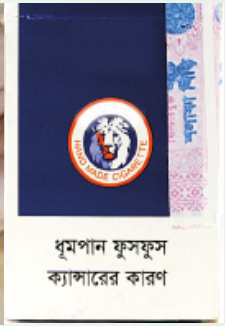

সিগারেটের প্যাকেট

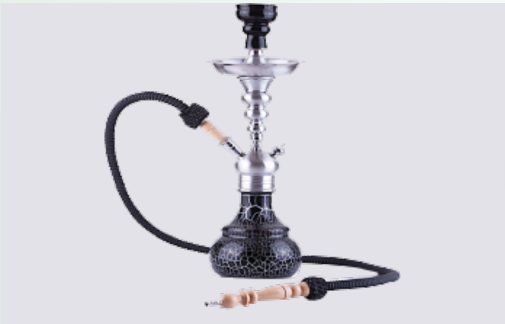

শিশা

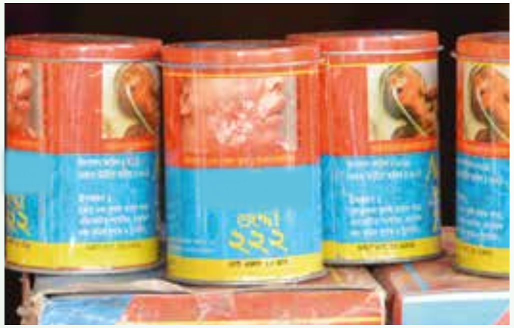

জর্দা

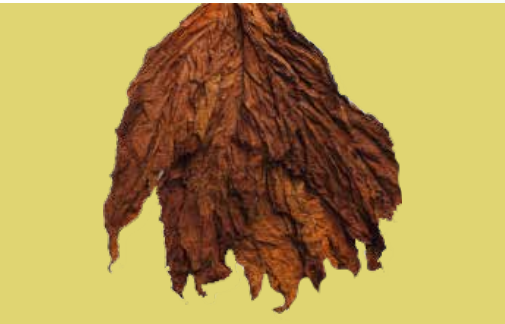

সাদাপাতা

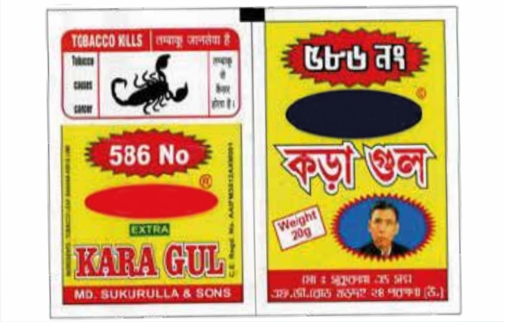

গুল

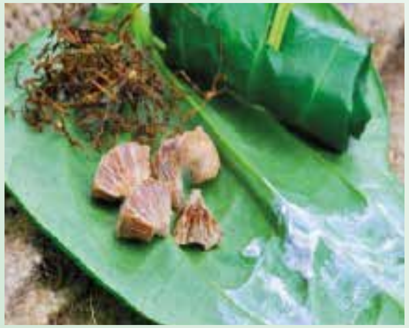

জর্দাসহ পান

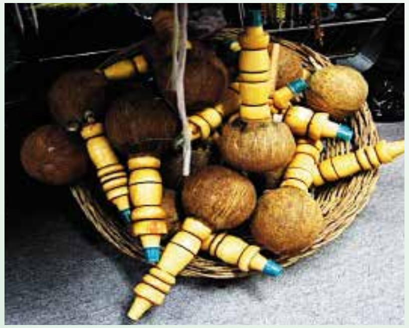

হুকা/বনেদি হুকা

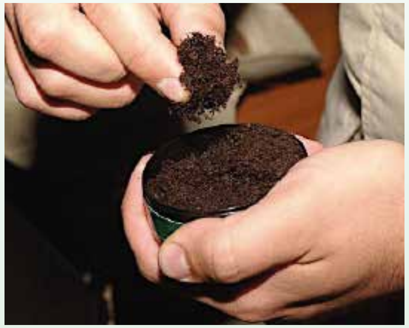

নস্যি (আর্দ্র)

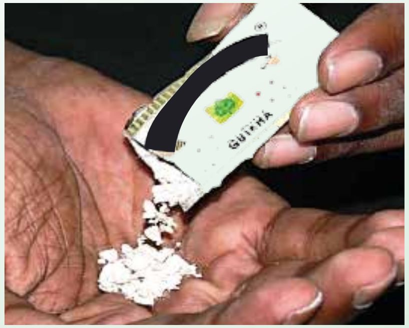

গুটকা

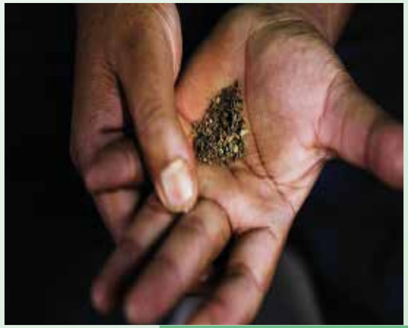

খৈনি

FOR COMMUNITY

বিভিন্ন ধরণের তামাকজাত দ্রব্য

তামাকজাত দ্রব্যের বিভিন্ন ধরন আছে। তামাকজাত দ্রব্যকে সাধারনত দুই ভাগে ভাগ করা যায়।

* **ধোঁয়াযুক্ত তামাক:** ধোঁয়াযুক্ত তামাকের মধ্যে রয়েছে সিগারেট, বিড়ি, সিগার, পাইপ এবং হুক্কা।
* **ধোঁয়া বিহীন তামাক:** ধোঁয়া বিহীন তামাকজাত দ্রব্যের মধ্যে রয়েছে জর্দা, নস্যি, গুল, সাদাপাতা ইত্যাদি।

* সমস্ত তামাকজাতীয় দ্রব্যে নিকোটিন থাকে, যা তামাকের পণ্য ব্যবহার করার সময় রক্ত প্রবাহে মিশে যায় এবং নেশার সৃষ্টি করে।
* তামাক জাতীয় পদার্থে ৭০০০ এর চেয়ে বেশি উপাদান থাকে, যার মধ্যে ক্যান্সারের ঝুঁকি বাড়ায় এমন ৬৯ ধরণের উপাদান রয়েছে।

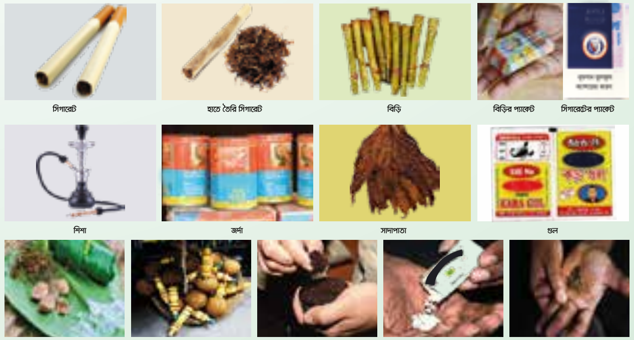

সিগারেট, হাতে তৈরি সিগারেট, বিড়ি, বিড়ির প্যাকেট, সিগারেটের প্যাকেট, শিশা, জর্দা, সাদাপাতা, গুল, জর্দাসহ পান, হুকা/বনেদি হুকা, নস্যি (আর্দ্র), গুটকা, খৈনি

FOR CHW

তথ্যসূত্র: বিশ্ব স্বাস্থ্য সংস্থা

তামাকের ক্ষতিকর প্রভাব

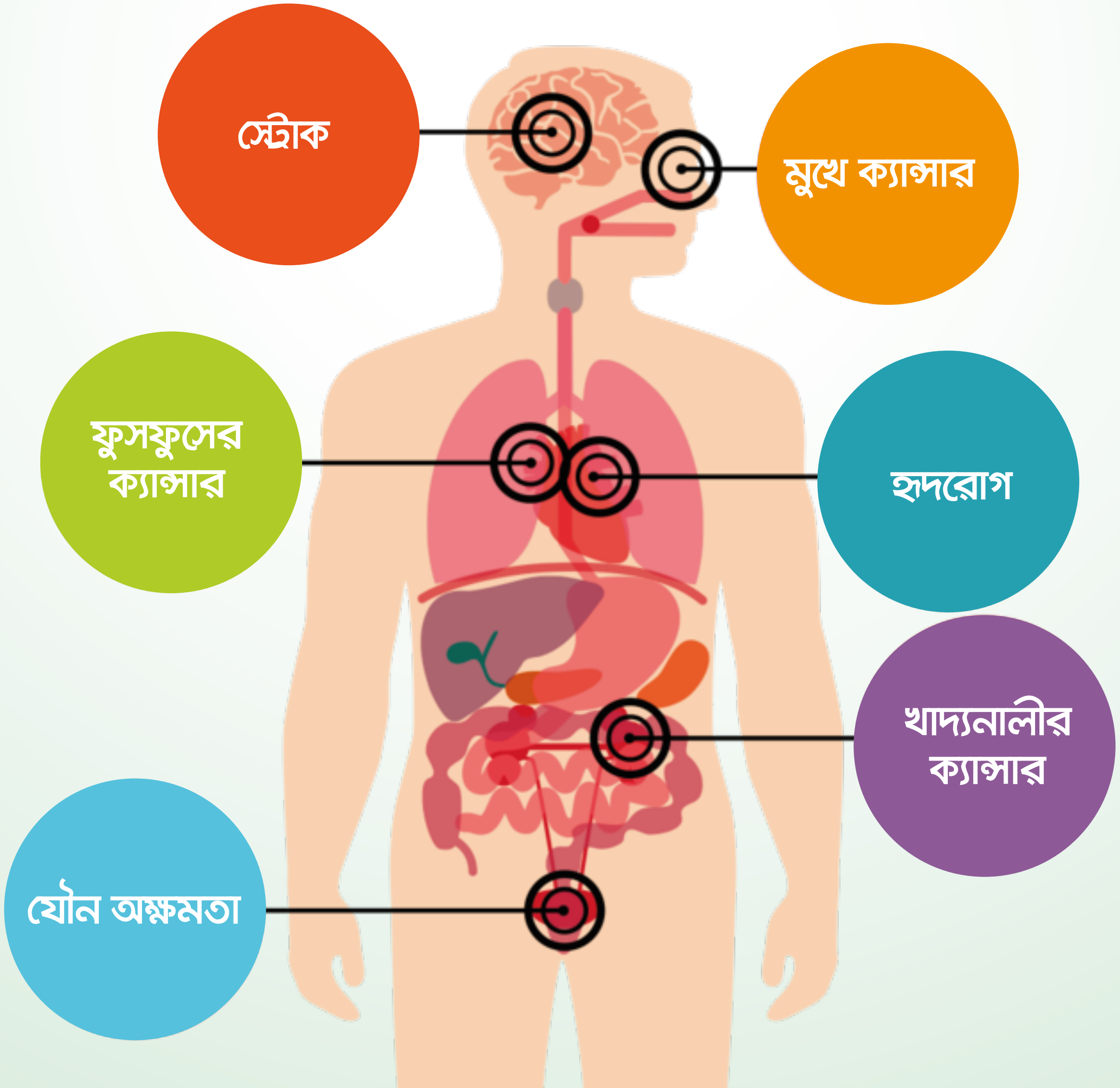

FOR COMMUNITY

তামাকের ক্ষতিকর প্রভাব

* তামাক জাতীয় দ্রব্য ব্যবহার করলে রক্তচাপ বেড়ে যায়। যা স্ট্রোকের ঝুঁকি বাড়িয়ে দেয়।
* তামাকজাত দ্রব্য ব্যবহারের ফলে সব ধরণের ক্যান্সারে মৃত্যুর হার বেশি। ধূমপায়ীদের মধ্যে ধূমপান জনিত ক্যান্সারে মৃত্যুর হার ৩০ শতাংশ।
* ফুসফুসের ক্যান্সারে আক্রান্ত হওয়ার ঝুঁকি অধূমপায়ীদের চেয়ে ধূমপায়ীদের মধ্যে ৪ থেকে ২৪ গুন বেশি।
* তামাকজাত দ্রব্য বাবহারকারীদের হৃদরোগে মৃত্যুর ঝুঁকির পরিমাণ অধূমপায়ীদের চেয়ে ২.৫ গুণ বেশি থাকে।
* তামাকজাত দ্রব্য ব্যবহার ছেড়ে দিলে, হৃদরোগের ঝুঁকির হার ৫৫ শতাংশ কমে যায়।
* তামাক মুখ এবং খাদ্যনালীতে ক্যান্সার সৃষ্টি করে।
* তামাকজাত দ্রব্য ব্যবহার পুরুষদের ক্ষেত্রে যৌন অক্ষমতা এবং মহিলাদের ক্ষেত্রে যৌন অনীহা তৈরি করে।

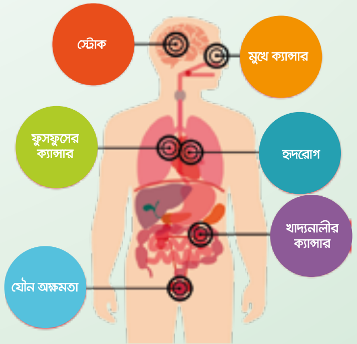

FOR CHW

তথ্যসূত্র: বিশ্ব স্বাস্থ্য সংস্থা

তামাক জাতীয় দ্রব্যে কিভাবে নেশা হয়

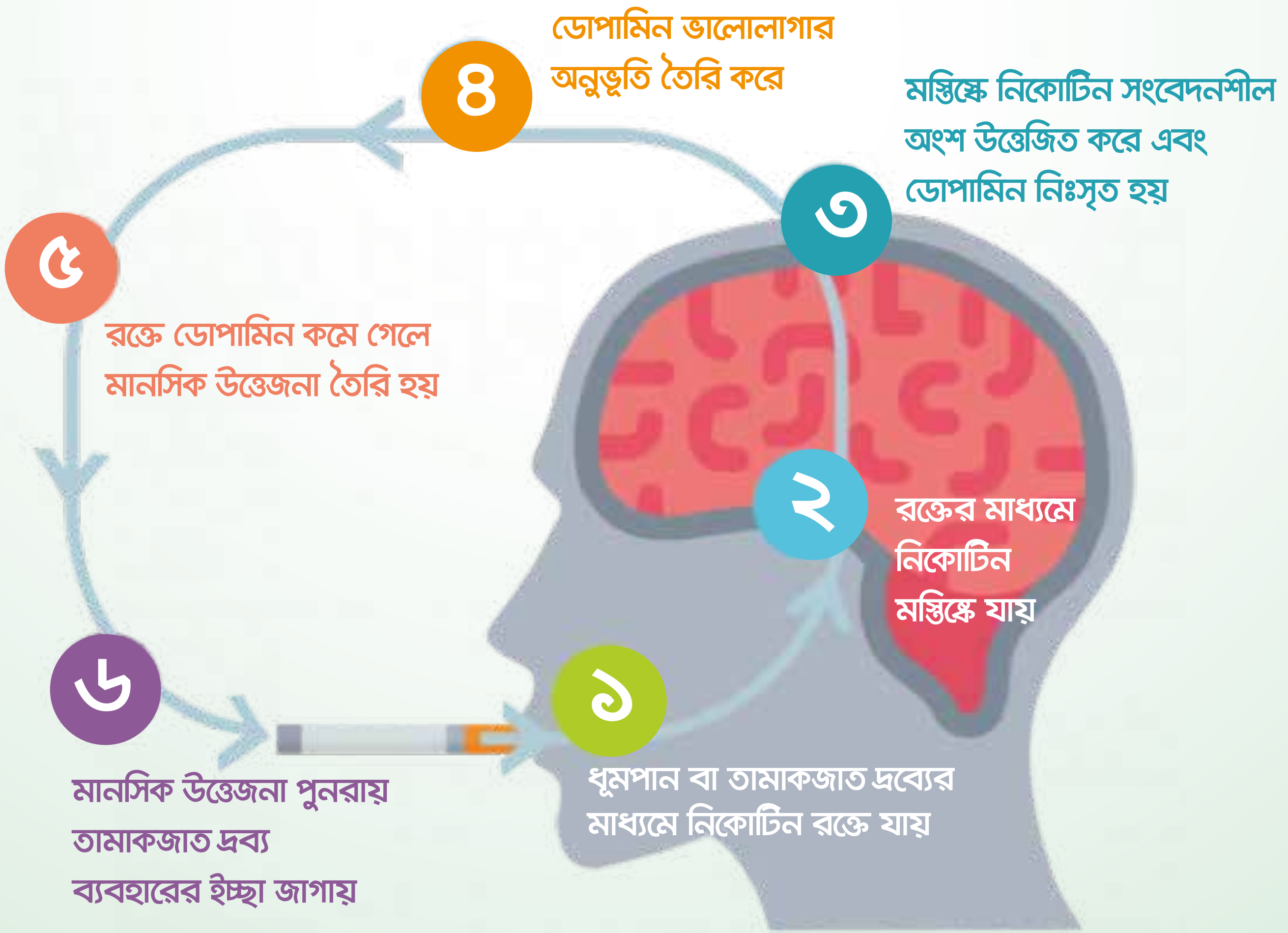

FOR COMMUNITY

তামাক জাতীয় দ্রব্যে কিভাবে নেশা হয়

* ধূমপান করলে নিকোটিন ফুসফুসের মাধ্যমে রক্তে যায়।
* রক্তের মাধ্যমে নিকোটিন মস্তিষ্কে যায়।
* নিকোটিন মস্তিষ্কের বিভিন্ন অংশকে উত্তেজিত করে এবং ডোপামিন নামের একটি পদার্থ নিঃসৃত হয় ।
* কেন ভাল লাগে: ডোপামিন মস্তিষ্কে সংবেদনশীল অংশে কাজ করে এবং ভাল লাগার অনুভূতি তৈরি করে।
* তামাকজাত দ্রব্য ব্যবহার কমে গেলে রক্তে ডোপামিন কমে যায় এবং মানসিক উত্তেজনা তৈরি হয়। উত্তেজনা কমানোর জন্য আবার ডোপামিনের প্রয়োজন হয় এবং সন্তুষ্টি অর্জনের জন্য তাই তামাক ব্যবহারকারীদের আবার তামাকের প্রয়োজন হয়।

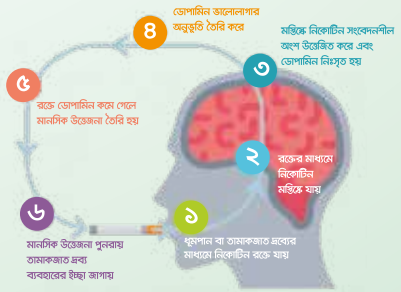

FOR CHW

তামাকজাত দ্রব্য ছেড়ে দেয়ার স্বাস্থ্য উপকারিতা

ধূমপান ছাড়ার পরে →

৮ ঘণ্টা
রক্তে নিকোটিন ও কার্বন মনোঅক্সাইড অর্ধেকে নেমে আসে

৪৮ ঘণ্টা
ধূমপানের ফলে ফুসফুসে জমে যাওয়া ময়লা পরিষ্কার হতে শুরু করে

৭২ ঘণ্টা
শ্বাস নেয়া সহজ হয়

২-১২ সপ্তাহ
রক্ত চলাচলে উন্নতি হয়

৩-৯ মাস
কাশি ও শ্বাসকষ্ট কমে আসে

১ বছর
হৃদরোগের ঝুঁকি অর্ধেক হয়ে যায়

১০ বছর
ক্যান্সারের ঝুঁকি ধূমপায়ীদের চেয়ে অর্ধেক হয়ে যায়

১৫ বছর
হার্ট অ্যাটাকের ঝুঁকি, অধূমপায়ী মানুষের মত হয়ে যায়

তথ্যসূত্র: বিশ্ব স্বাস্থ্য সংস্থা

FOR COMMUNITY

তামাকজাত দ্রব্য ছেড়ে দেয়ার স্বাস্থ্য উপকারিতা

যত দ্রুত আপনি ধূমপান ছেড়ে দেবেন, তত বেশি সুবিধা পাবেন। ধূমপান ছাড়ার নির্দিষ্ট সময় পর থেকে স্বাস্থ্যে যে উন্নতিগুলো লক্ষ্য করবেন-

৮ ঘণ্টা পরঃ ধূমপান বন্ধের ৮ ঘণ্টার মধ্যে রক্তের নিকোটিন ও কার্বন মনোঅক্সাইডের পরিমান অর্ধেকে নেমে আসে।
৪৮ ঘন্টা পরঃ ধূমপানের ফলে ফুসফুসে জমে যাওয়া ময়লা পরিষ্কার হতে শুরু করে।
৭২ ঘণ্টা পরঃ শ্বাস নেয়া তুলনামূলক ভাবে সহজ হয়।
২ থেকে ১২ সপ্তাহ পরঃ রক্ত চলাচলে উন্নতি হয়।
৩ থেকে ৯ মাস পরঃ কাশি ও শ্বাসকষ্ট কমে আসে।
১ বছর পরঃ হৃদরোগের ঝুঁকি অর্ধেক হয়ে যায়।
১০ বছর পরঃ ক্যান্সারের ঝুঁকি ধূমপায়ীদের চেয়ে অর্ধেক হয়ে যায়।
১৫ বছর পরঃ হার্ট অ্যাটাকের ঝুঁকি, অধূমপায়ী মানুষের মত হয়ে যায় ।

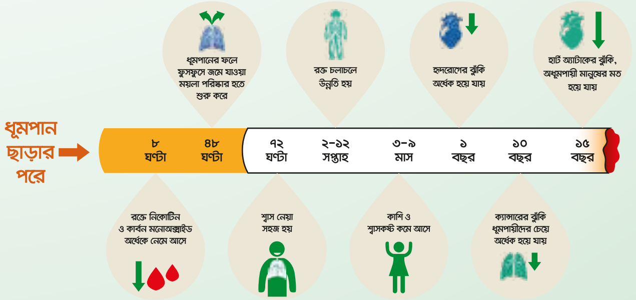

FOR CHW

ধূমপান ও তামাকজাত দ্রব্য ছাড়ার উপায়

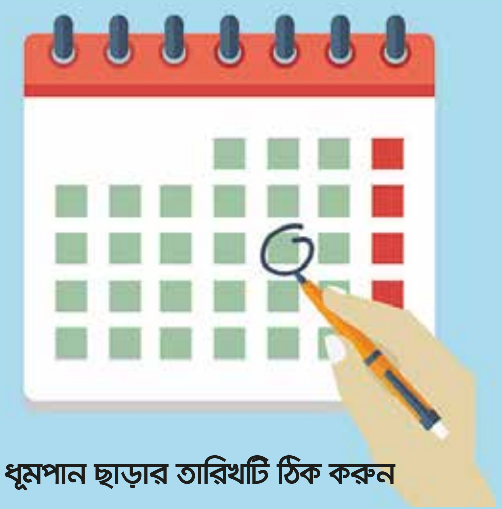

ধূমপান ছাড়ার তারিখটি ঠিক করুন

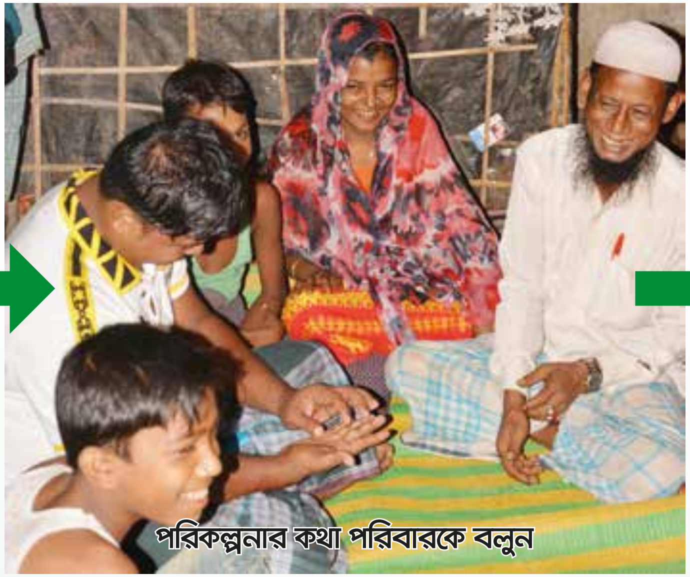

পরিকল্পনার কথা পরিবারকে বলুন

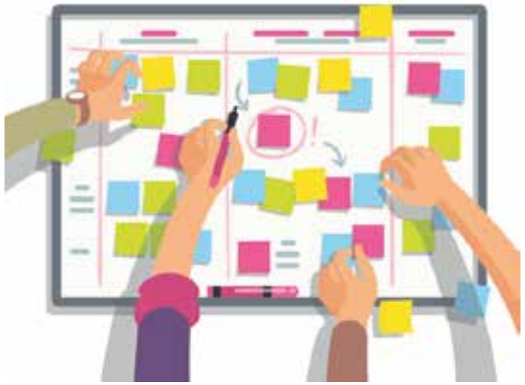

ধূমপান ও তামাকজাত দ্রব্য ছাড়ার কারন ও বাঁধাগুলোর বিপক্ষে প্রস্তুতি নিন

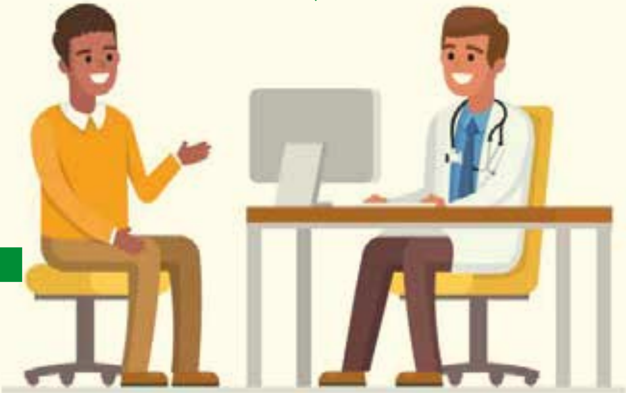

সাহায্যের জন্য ডাক্তার বা স্বাস্থ্যকর্মীর পরামর্শ নিন

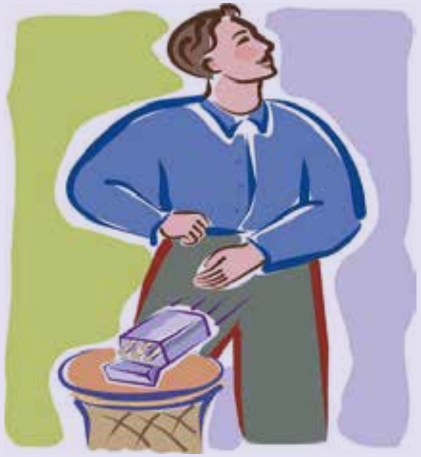

সকল তামাক জাতীয় দ্রব্য ফেলে দিন

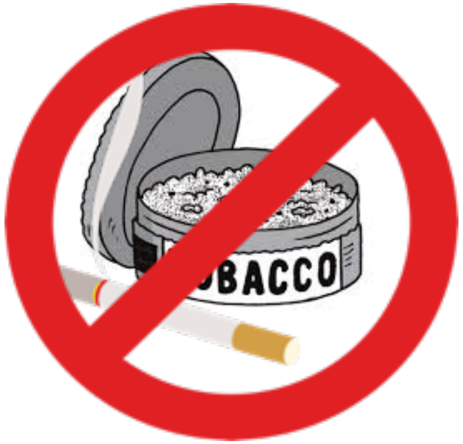

ধূমপান ও তামাকজাত দ্রব্য বর্জন করুন

FOR COMMUNITY

ধূমপান ও তামাকজাত দ্রব্য ছাড়ার উপায়

ধূমপান ও তামাকজাত দ্রব্য ব্যবহার ছাড়া সম্ভব, কিভাবে ছাড়তে পারেন

* ধূমপান বা তামাকজাত দ্রব্যের ব্যবহার ছাড়ার তারিখটি ঠিক করুন। এমন একটি তারিখ ঠিক করুন যা ১ বা ২ সপ্তাহ পর, যাতে আপনি ধূমপান বা তামাকজাত দ্রব্য ব্যবহার ছাড়ার জন্য প্রস্তুত হতে পারেন। যদি সম্ভব হয় তবে এমন একটি সময় নির্দিষ্ট করুন, যখন আপনার জীবনে কোন পরিবর্তন হবে বা এমন সময় বেছে নিন, যখন আপনার কর্মস্থানে বা বাড়িতে কোনও অতিরিক্ত চাপ থাকবে না।
* আপনার বন্ধু এবং পরিবারের সদস্যদের আপনার ধূমপান বা তামাকজাত দ্রব্য ব্যবহার ছাড়ার পরিকল্পনার কথা বলুন।
* আপনি যে কারণে ধূমপান বা তামাকজাত দ্রব্য ব্যবহার ছাড়তে চান এবং ছাড়ার বাঁধাগুলোর একটি তালিকা তৈরি করুন। তালিকাটি কাছে রাখুন, যাতে আপনার ধূমপান বা তামাকজাত দ্রব্যের ব্যবহারের ইচ্ছা হলেই আপনি এটি দেখতে পান। আপনি কোথায়, কখন এবং কেন ধূমপান করেন, সে কারণটি নিয়ে চিন্তা করুন।
* ধূমপান বা তামাকজাত দ্রব্যের ব্যবহার ছাড়ার জন্য প্রয়োজনে ডাক্তার বা স্বাস্থ্যকর্মীর পরামর্শ নিন।
* আপনার কাছে থাকা সব তামাক জাতীয় দ্রব্য ফেলে দিন।
* নির্দিষ্ট দিনে ধূমপান বা তামাকজাত দ্রব্য ব্যবহার করা ছেড়ে দিন।

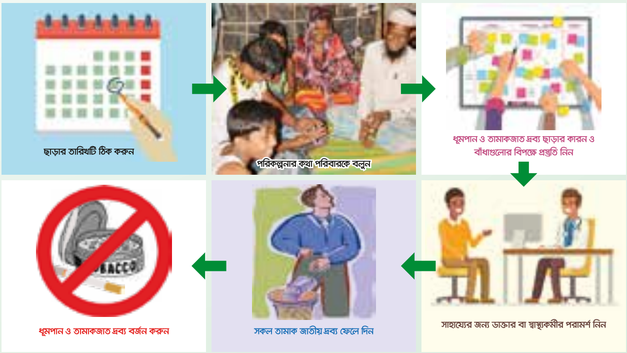

FOR CHW

অতিরিক্ত ওজন ও স্থূলতা

অতিরিক্ত ওজন ও স্থূলতার কারণ কী?

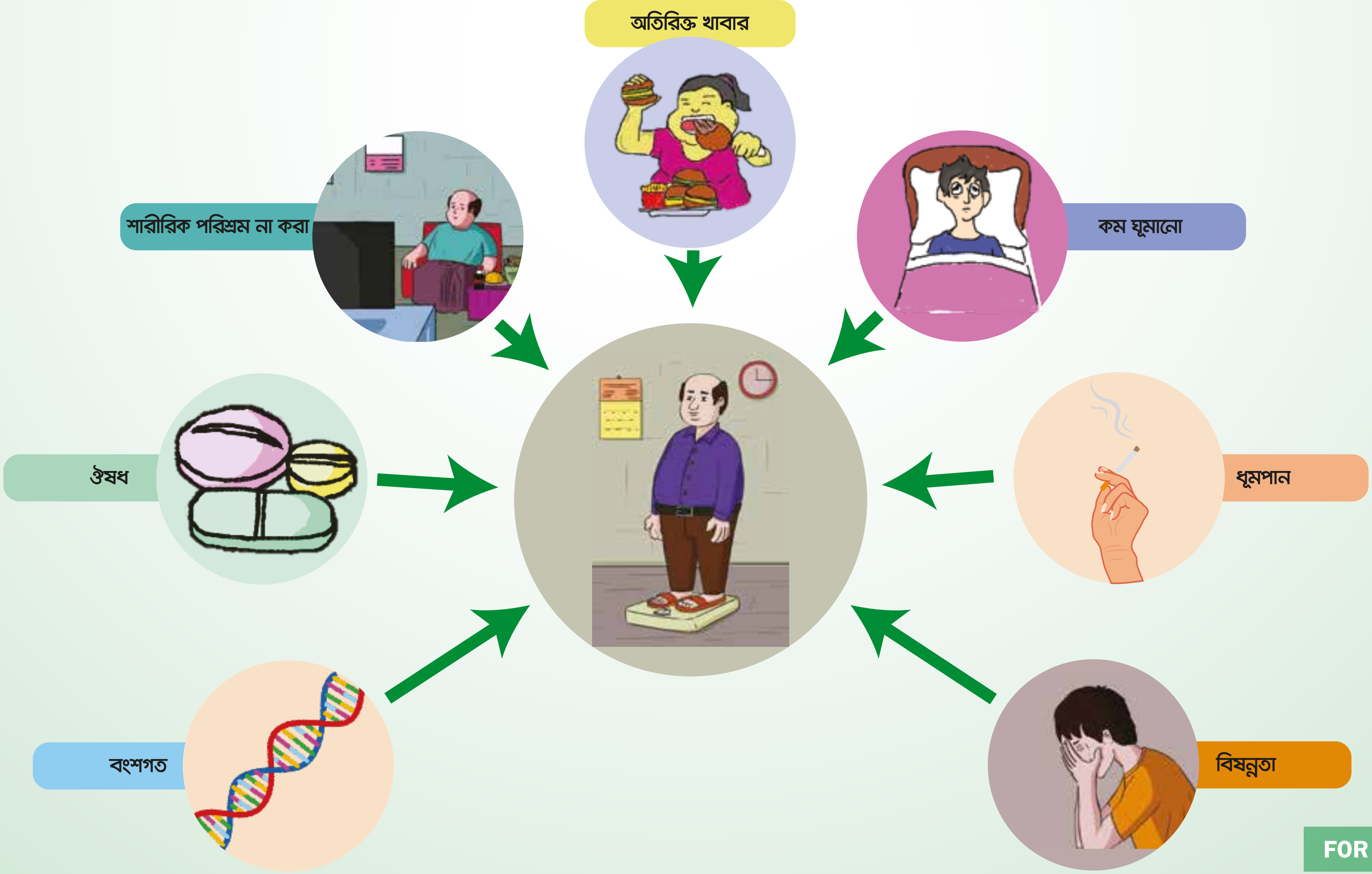

 তথ্যসূত্র: জাপান ইন্টারন্যাশনাল কোঅপারেশন এজেন্সি (জাইকা)

অতিরিক্ত ওজন ও স্থূলতা

বাড়তি ওজন ও স্থূলতার কারন

বাড়তি ওজন ও স্থূলতার কারন হল খাবার গ্রহন ও খাবার থেকে তৈরি শক্তি খরচের তারতম্য। বর্তমানে যেসব খাবারে প্রচুর চর্বি, শকর্রা ও চিনি থাকে, সে সব খাবার খাওয়ার প্রবনতা বেড়ে গেছে। আবার কাজের ধরন, পরিবহন ব্যবস্থা এবং নগরায়নের কারনে শারীরিক পরিশ্রম করার প্রবনতা কমে গেছে। এছাড়াও বংশগতি, ঔষধ, ধূমপান, কম ঘুমানো, বিষন্নতা পরোক্ষভাবে ওজন বাড়ানোর জন্য দায়ী।

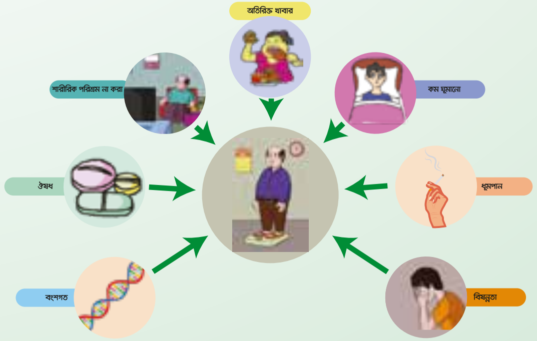

FOR CHW

শারীরিক পরিশ্রমের উপকারিতা

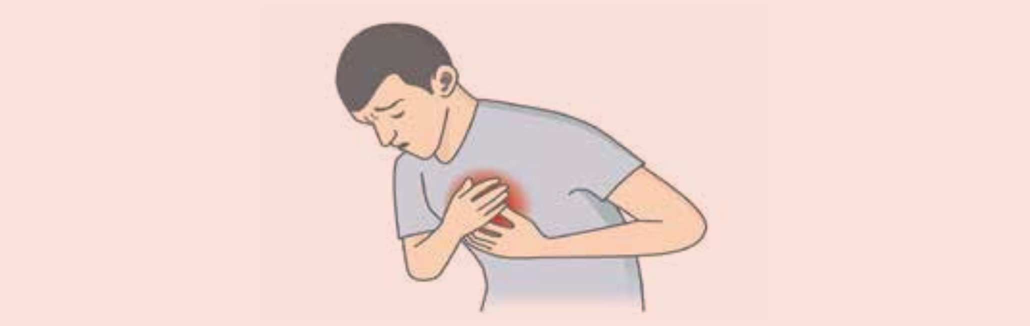

হৃদরোগের ঝুঁকি কমায়

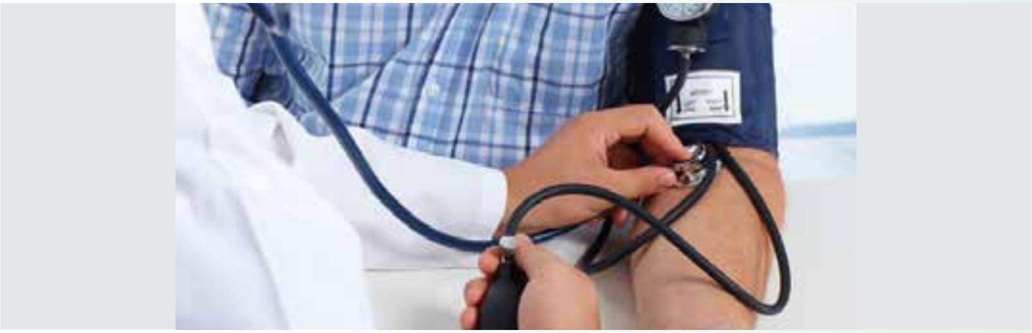

উচ্চ রক্তচাপ নিয়ন্ত্রণ করে

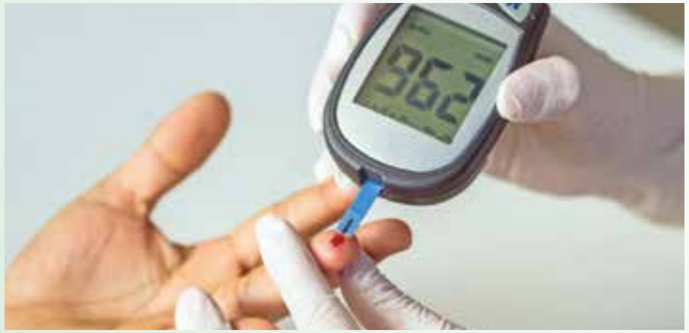

ডায়াবেটিস নিয়ন্ত্রণ করে

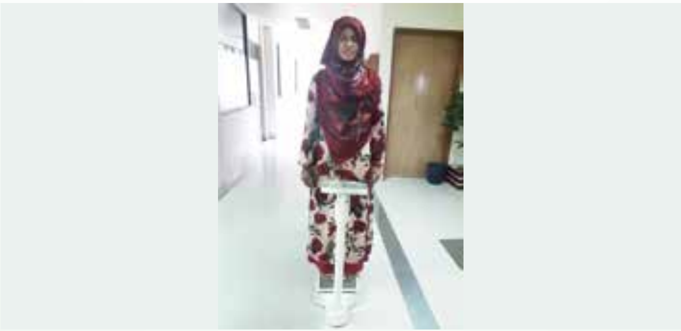

ওজন নিয়ন্ত্রণে রাখে

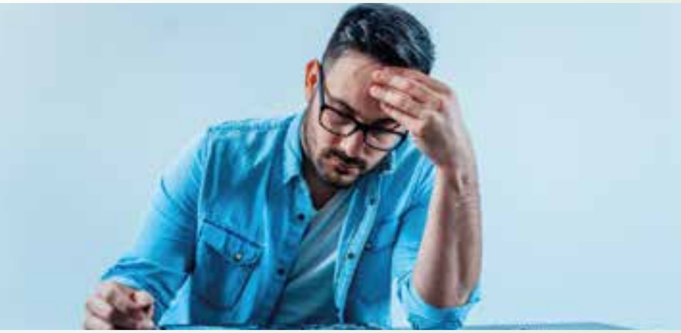

মানসিক চাপ থেকে মুক্তি দেয়

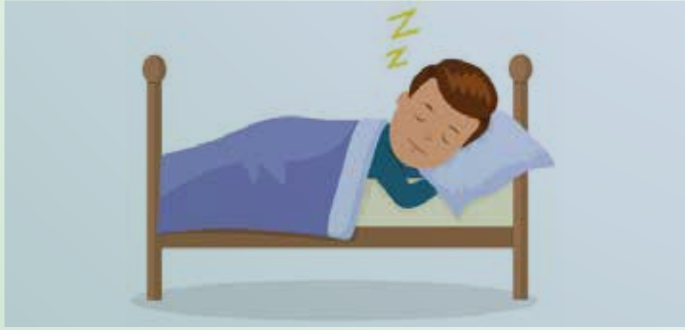

ভাল ঘুমে সহায়তা করে

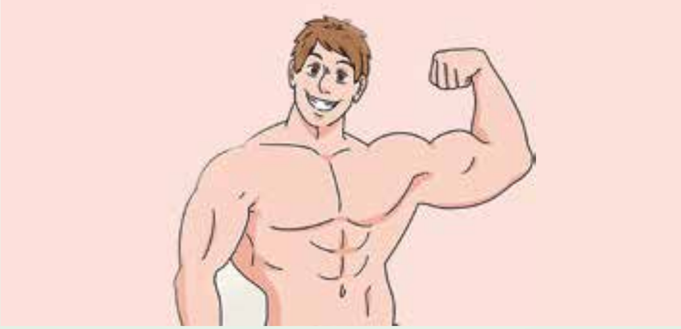

পেশী শক্তিশালী ও হাড় মজবুত করে

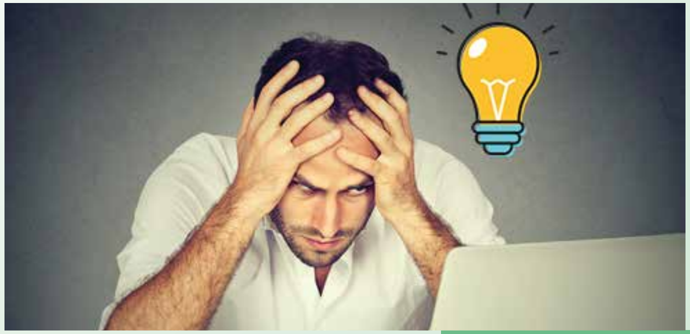

স্মৃতিশক্তি বাড়াতে সহায়তা করে

তথ্যসুত্র: জাপান ইন্টারন্যাশনাল কোঅপারেশন এজেন্সি (জাইকা)

শারীরিক পরিশ্রমের উপকারিতা

নিয়মিত শারীরিক পরিশ্রম করলে শারীরিক ও মানসিক অবস্থার উন্নতি হয়। শারীরিক পরিশ্রমের উপকারিতাগুলো হলঃ

* হৃদরোগ, স্ট্রোক, ক্যান্সার ও ডায়াবেটিসের ঝুঁকি কমে যায়।
* উচ্চ রক্তচাপ নিয়ন্ত্রনে থাকে।
* ডায়াবেটিস নিয়ন্ত্রনে থাকে।
* ওজন কমে এবং নিয়ন্ত্রনে থাকে।
* বিষন্নতা কমে যায় এবং নিয়ন্ত্রনে থাকে।
* ঘুম ভাল হয়।
* পেশী শক্তিশালী ও হাড় মজবুত হয়।
* স্মৃতিশক্তি বাড়াতে সাহায্য করে।

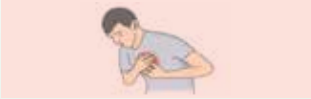
হৃদরোগের ঝুঁকি কমায়

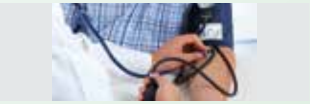
উচ্চ রক্তচাপ নিয়ন্ত্রণ করে

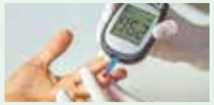
ডায়াবেটিস নিয়ন্ত্রণ করে

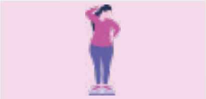
ওজন নিয়ন্ত্রণে রাখে

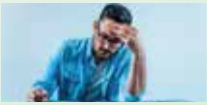
মানসিক চাপ থেকে মুক্তি দেয়

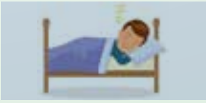
ভাল ঘুমে সহায়তা করে

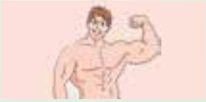
পেশী শক্তিশালী ও হাড় মজবুত করে

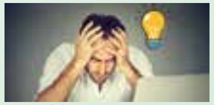
স্মৃতিশক্তি বাড়াতে সহায়তা করে

FOR CHW

বিভিন্ন ধরণের শারীরিক পরিশ্রম

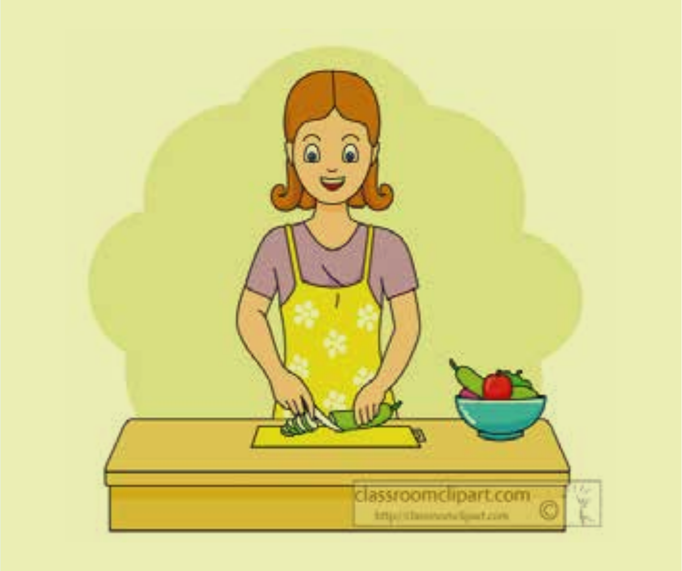

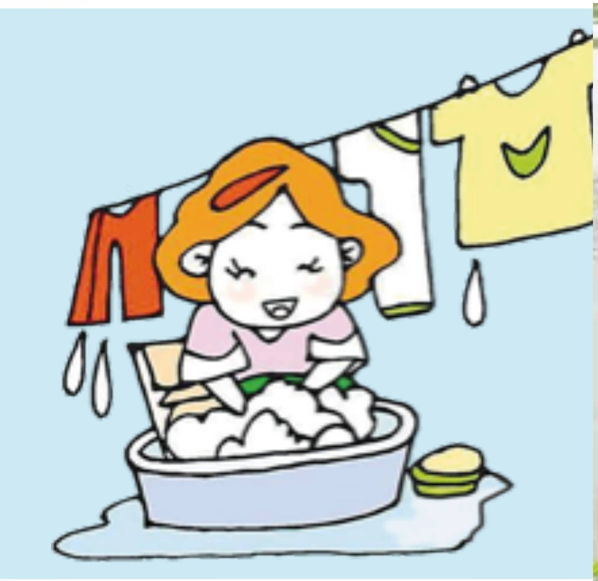

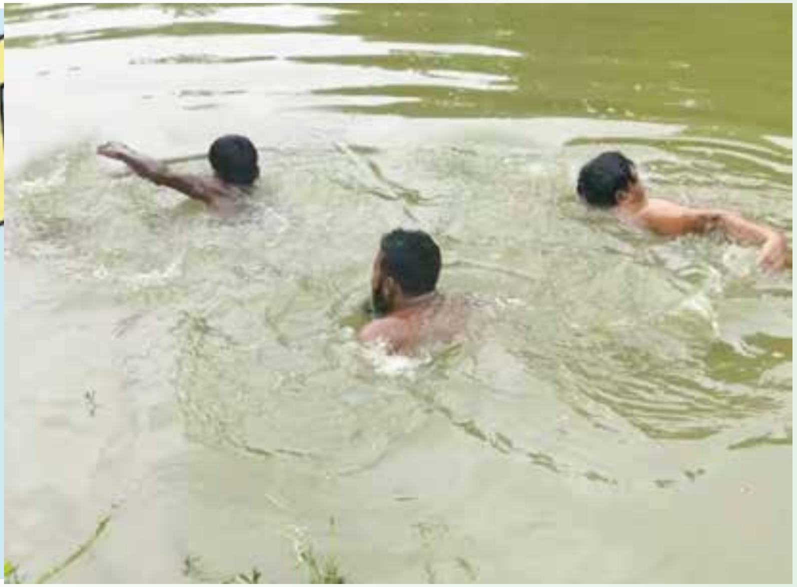

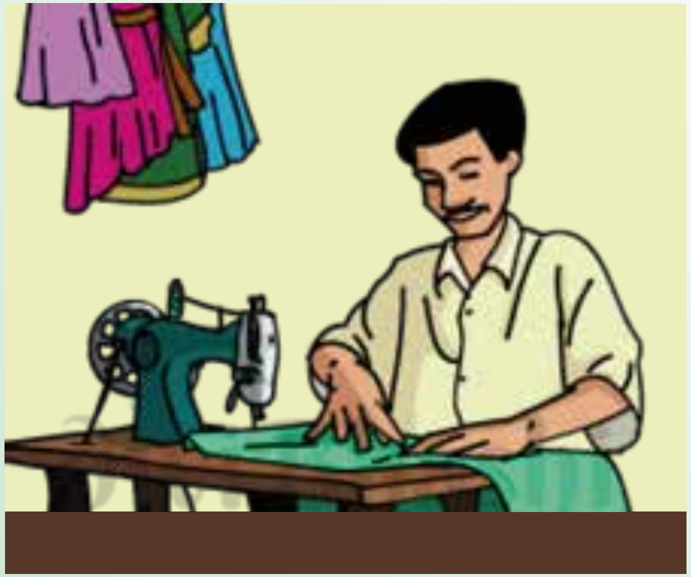

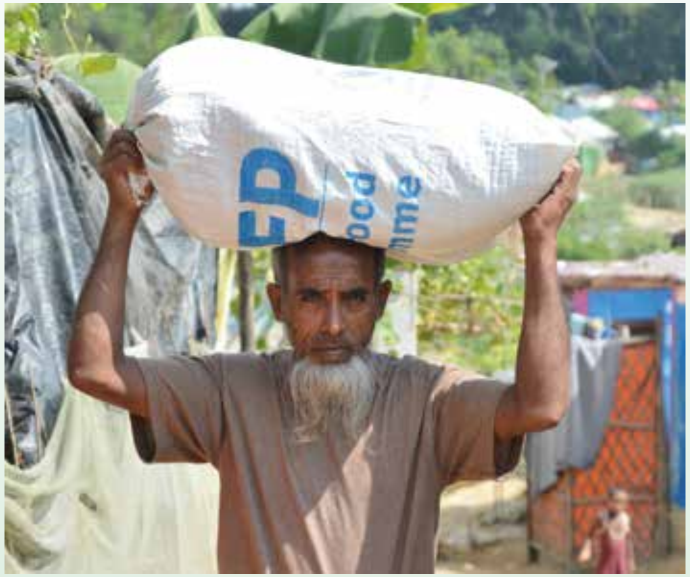

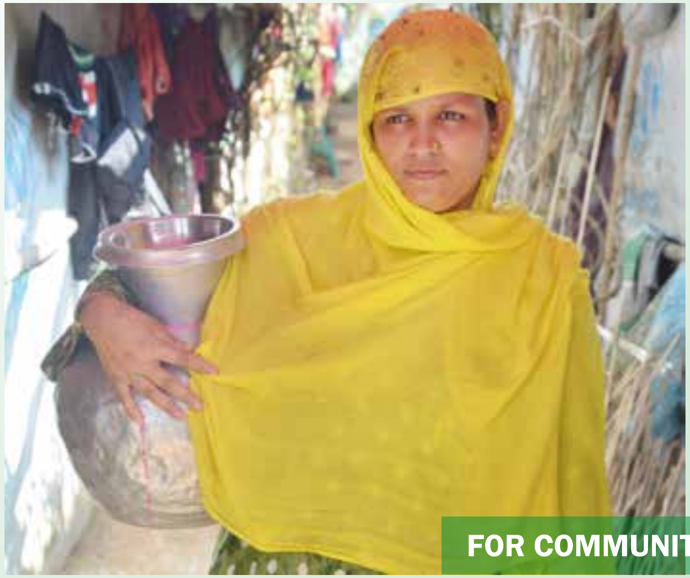

FOR COMMUNITY

বিভিন্ন ধরণের শারীরিক পরিশ্রম

শারীরিক পরিশ্রম বিভিন্ন ধরণের হতে পারে

হালকা কাজঃ যে সব কাজ করলে শ্বাস প্রশ্বাস দ্রুত হয় না।
মাঝারি কাজঃ যে সব কাজ করলে শ্বাস প্রশ্বাস অল্প দ্রুত হয়।
ভারী কাজঃ যে সব কাজ করলে শ্বাস প্রশ্বাস বেশি দ্রুত হয়।

অসংক্রামক রোগ প্রতিরোধ ও প্রতিকারের জন্য মাঝারী মাত্রার কাজ ও ভারী কাজ গুরুত্বপূর্ণ।

এই কাজগুলি কমপক্ষে একটানা ১০ মিনিট ধরে করতে হবে। তবেই, শারীরিক সুবিধা পাওয়া যাবে।

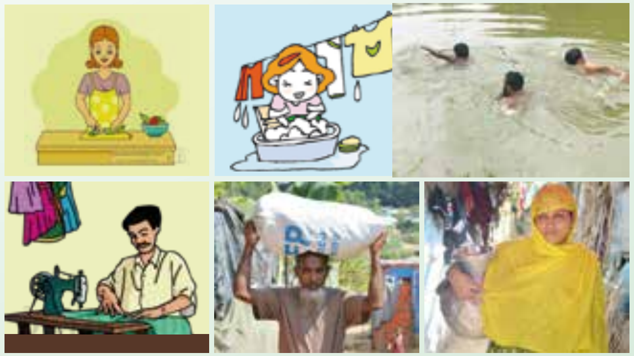

FOR CHW

মাঝারি কাজ

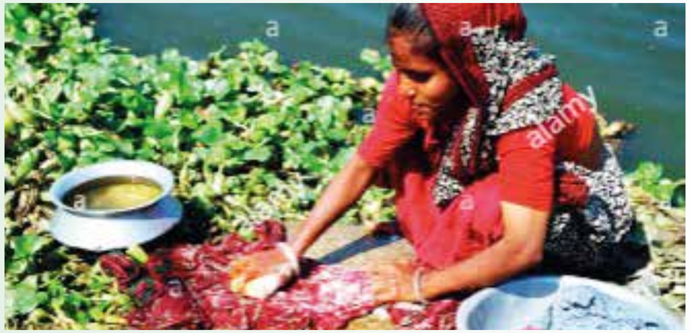

কাপড় ধোয়া

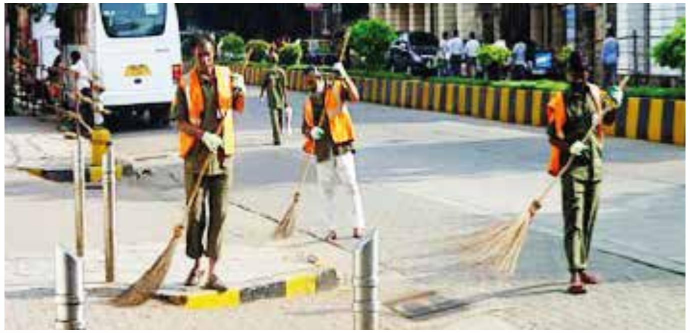

ঝাঁড়ু দেয়া

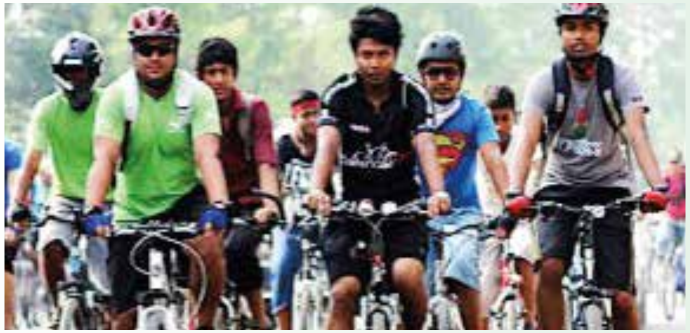

সাইকেল চালানো

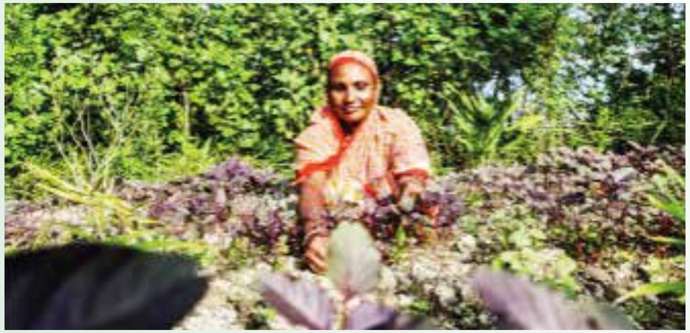

বাগান করা

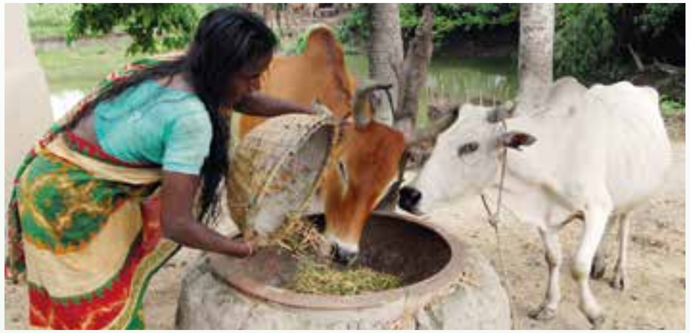

পশু পালন

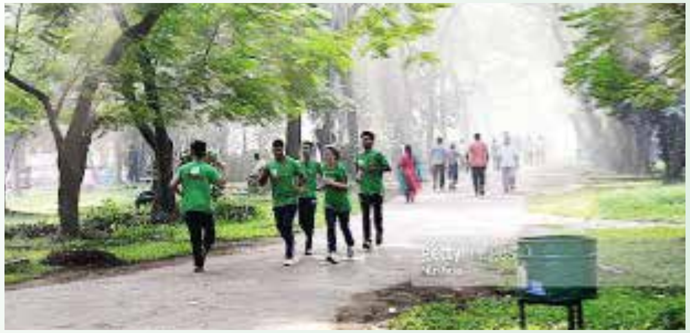

জগিং

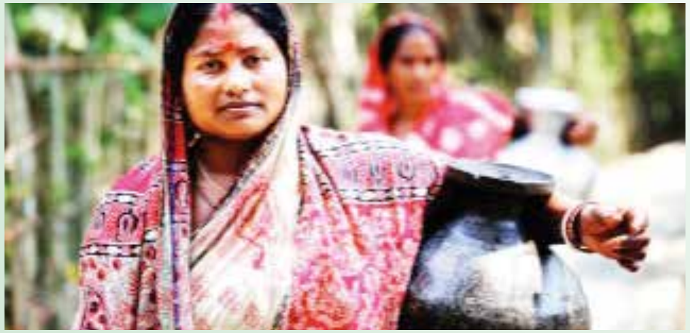

পানি বহন করা

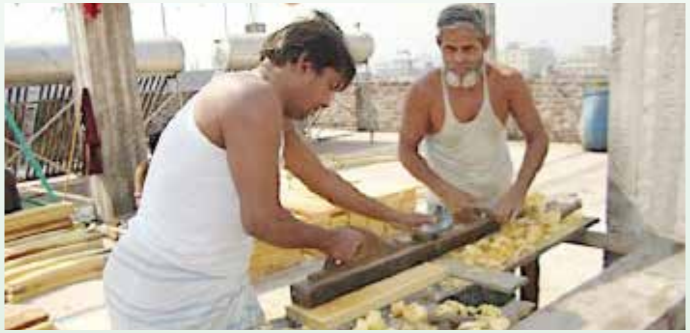

রেদা বা কুড়নি দিয়ে চাঁছার কাজ করা

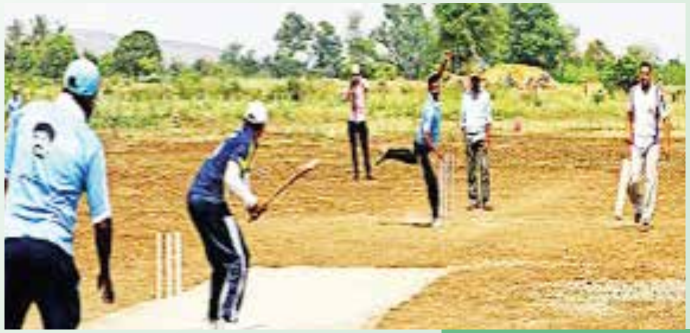

ক্রিকেট

মাঝারি কাজ

মাঝারি কাজের মধ্যে রয়েছে কাপড় ধোয়া, ঝাড়ু দেয়া, সাইকেল চালানো, বাগান করা, পশু পালন, পানি আনা, গৃহস্থালির অন্যান্য কাজ করা, ক্রিকেট খেলা ইত্যাদি।

মাঝারী কাজ শুরু করলে কমপক্ষে একটানা ১০ মিনিট ধরে করা উচিত।
তবেই, শারীরিক সুবিধা পাওয়া যাবে।

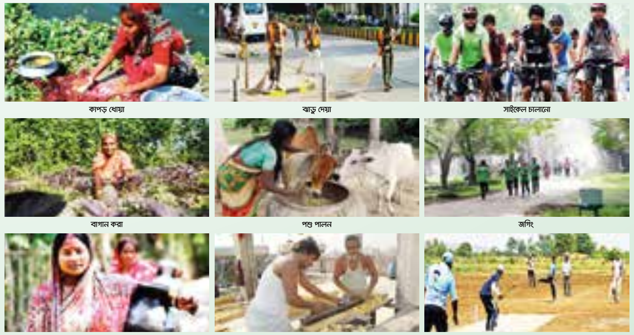

কাপড় ধোয়া | ঝাড়ু দেয়া | সাইকেল চালানো

বাগান করা | পশু পালন | জগিং

পানি বহন করা | রেদা বা কুড়নি দিয়ে চাঁছার কাজ করা | ক্রিকেট

FOR CHW

ভারী কাজ

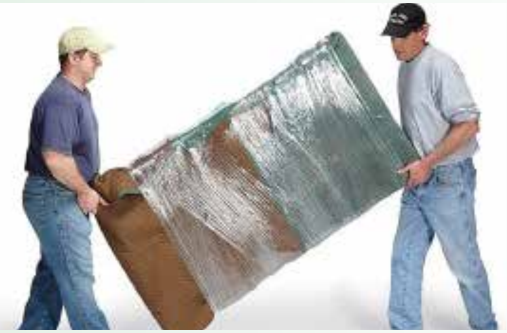
ভারী জিনিস বহন করা বা তোলা

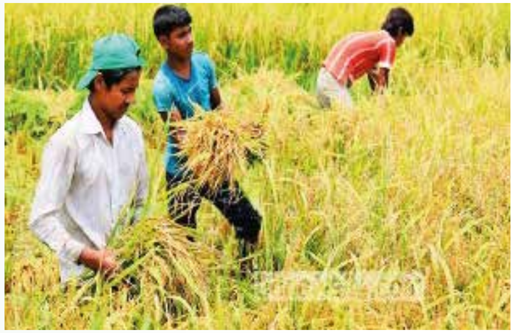
ফসল কাটা

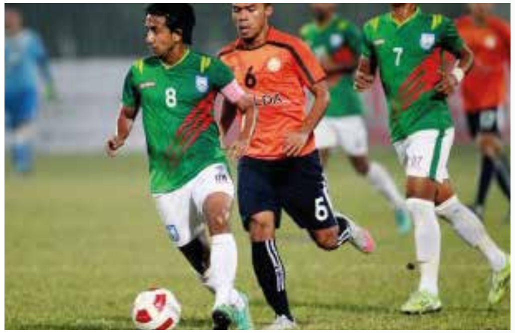
ফুটবল

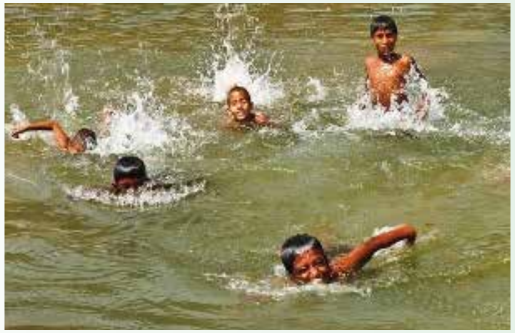
দ্রুত সাঁতার কাটা

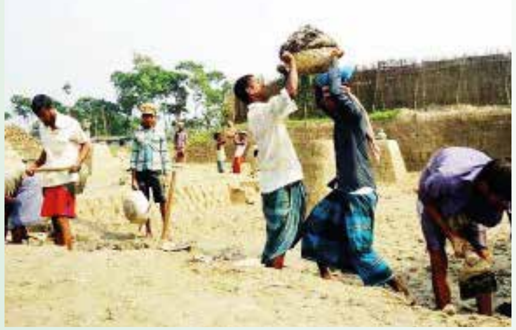
মাটি কাটা

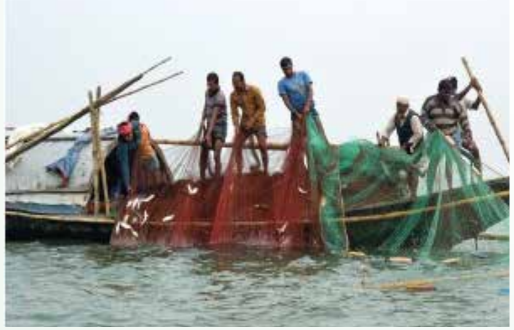
জাল দিয়ে মাছ ধরা

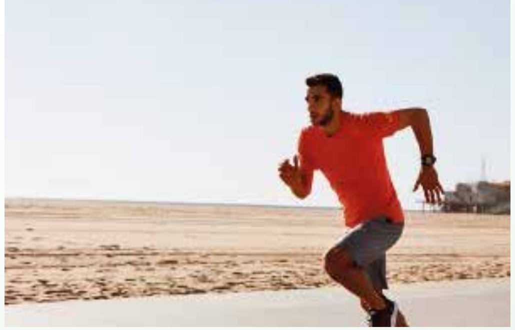
দ্রুত দৌড়ানো

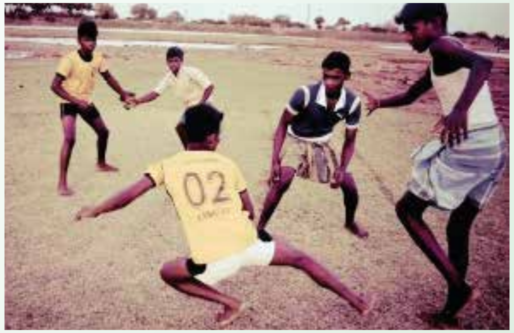
কাবাডি খেলা

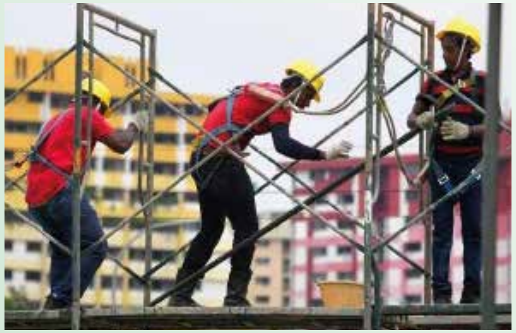
নির্মাণ কাজ

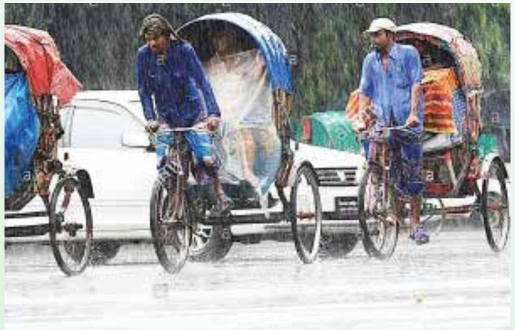
রিকশা চালানো

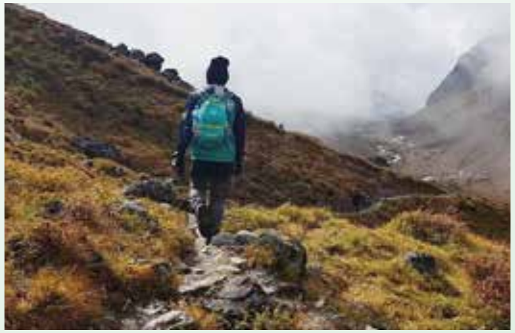
পাহাড় বেয়ে উপরে উঠা

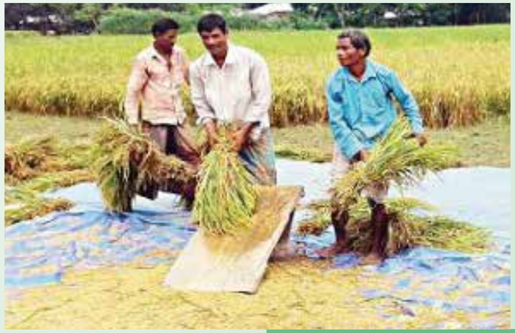
ধান মাড়াই

ভারী কাজ

ভারী কাজের সময় আপনার শ্বাস প্রশ্বাস স্বাভাবিকের চেয়ে অনেক বেশি হয়

ভারী কাজের মধ্যে রয়েছে ভারী জিনিস (২০ কেজির বেশি) বহন করা বা তোলা, ফসল কাটা, দ্রুত সাঁতার কাটা, মাটি কাটা, জাল দিয়ে মাছ ধরা, দ্রুত দৌড়ানো, কাবাডি খেলা, নির্মাণ কাজ, রিকশা চালানো, পাহাড় বেয়ে উপরে উঠা, ধান মাড়াই ইত্যাদি।

এই কাজগুলি শুরু করলে কমপক্ষে একটানা ১০ মিনিট ধরে করা উচিত।
তবেই, শারীরিক সুবিধা পাওয়া যাবে।

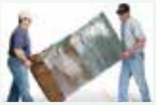
ভারী জিনিস বহন করা বা তোলা

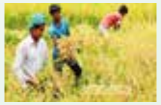
ফসল কাটা

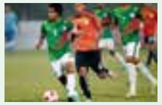
ফুটবল

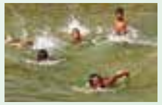
দ্রুত সাঁতার কাটা

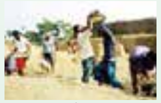
মাটি কাটা

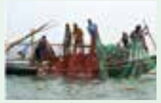
জাল দিয়ে মাছ ধরা

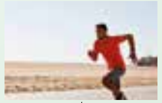
দ্রুত দৌড়ানো

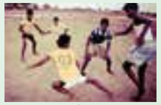
কাবাডি খেলা

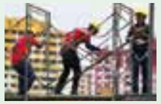
নির্মাণ কাজ

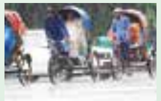
রিকশা চালানো

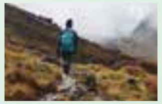
পাহাড় বেয়ে উপরে উঠা

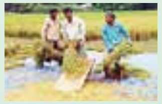
ধান মাড়াই

FOR CHW

সপ্তাহে কতক্ষণ শারীরিক পরিশ্রম করা প্রয়োজন?

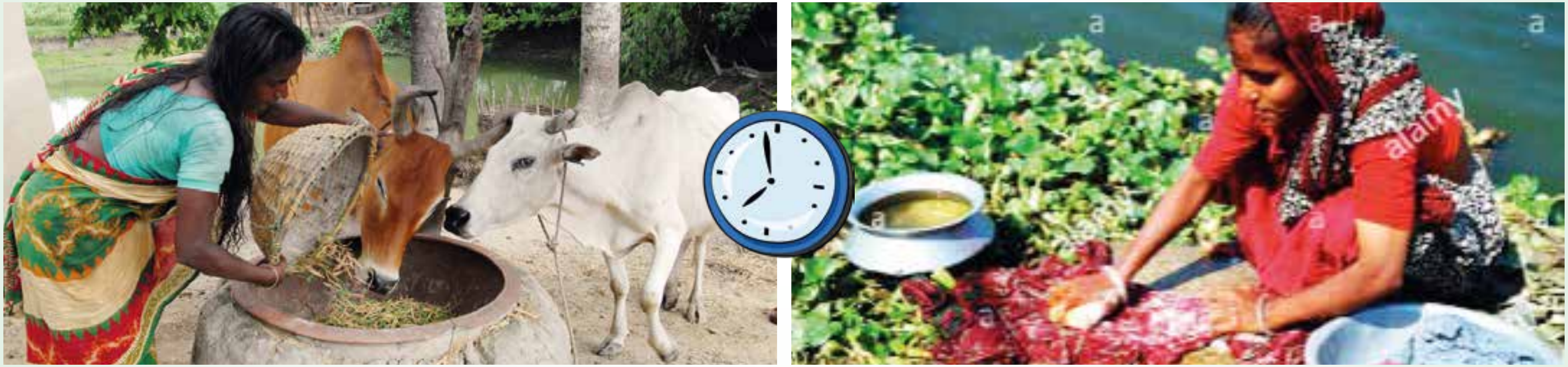

মাঝারি কাজের ক্ষেত্রে সপ্তাহে কমপক্ষে ১৫০ মিনিট

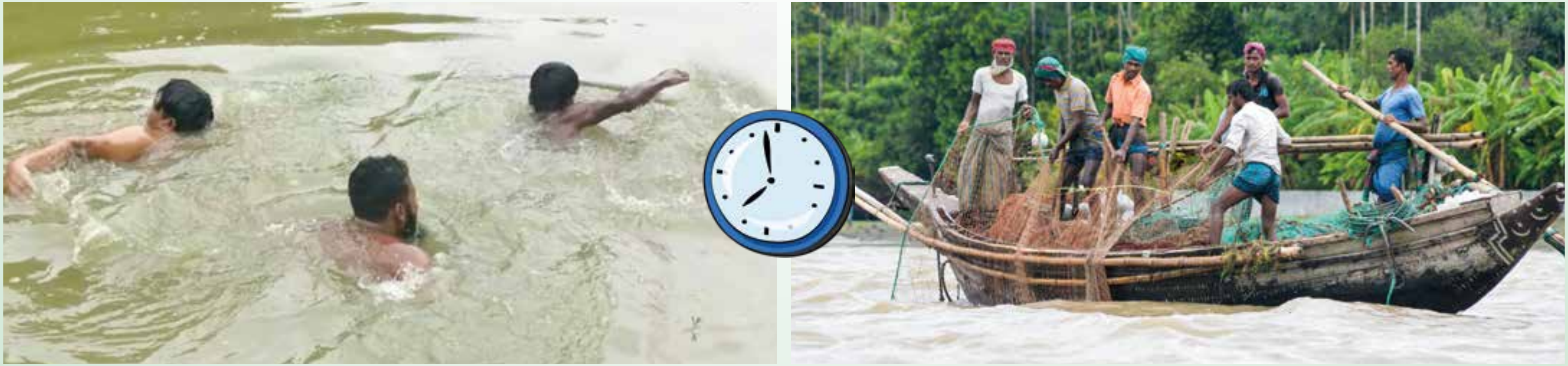

ভারী কাজের ক্ষেত্রে সপ্তাহে কমপক্ষে ৭৫ মিনিট

সপ্তাহে কতক্ষণ শারীরিক পরিশ্রম করা প্রয়োজন?

মাঝারি কাজের ক্ষেত্রে সপ্তাহে কমপক্ষে ১৫০ মিনিট (আড়াই ঘন্টা)
অথবা
সপ্তাহে ৫ দিন, প্রতিদিন কমপক্ষে ৩০ মিনিট।

ভারী কাজের ক্ষেত্রে সপ্তাহে কমপক্ষে ৭৫ মিনিট (সোয়া ঘন্টা)
অথবা
সপ্তাহে ৫ দিন, প্রতিদিন কমপক্ষে ১৫ মিনিট।

একটানা দুই দিনের বেশি বিশ্রাম নেবেন না। শারীরিক পরিশ্রম শুরুর আগে চিকিৎসকের পরামর্শ নিন।

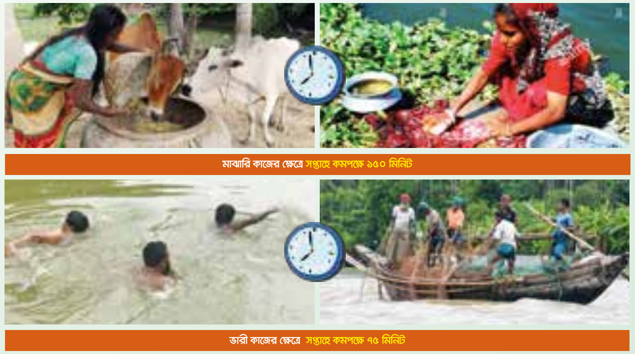

FOR CHW

উচ্চ রক্তচাপের লক্ষন সমূহ

উচ্চ রক্তচাপের বেশিরভাগ রোগীর কোনও লক্ষণই থাকেনা। কারো কারো ক্ষেত্রে নিম্নের লক্ষণগুলো থাকতে পারে।

সাধারণ লক্ষণসমূহ

* মাথা ব্যাথা
* ঘাড়ে ব্যাথা
* চোখে ঝাপসা দেখা
* মাথা ঘোরা
* কানে ভোঁ ভোঁ শব্দ শোনা
* শ্বাস প্রশ্বাস দ্রুত হওয়া
* নাক দিয়ে রক্ত পড়া

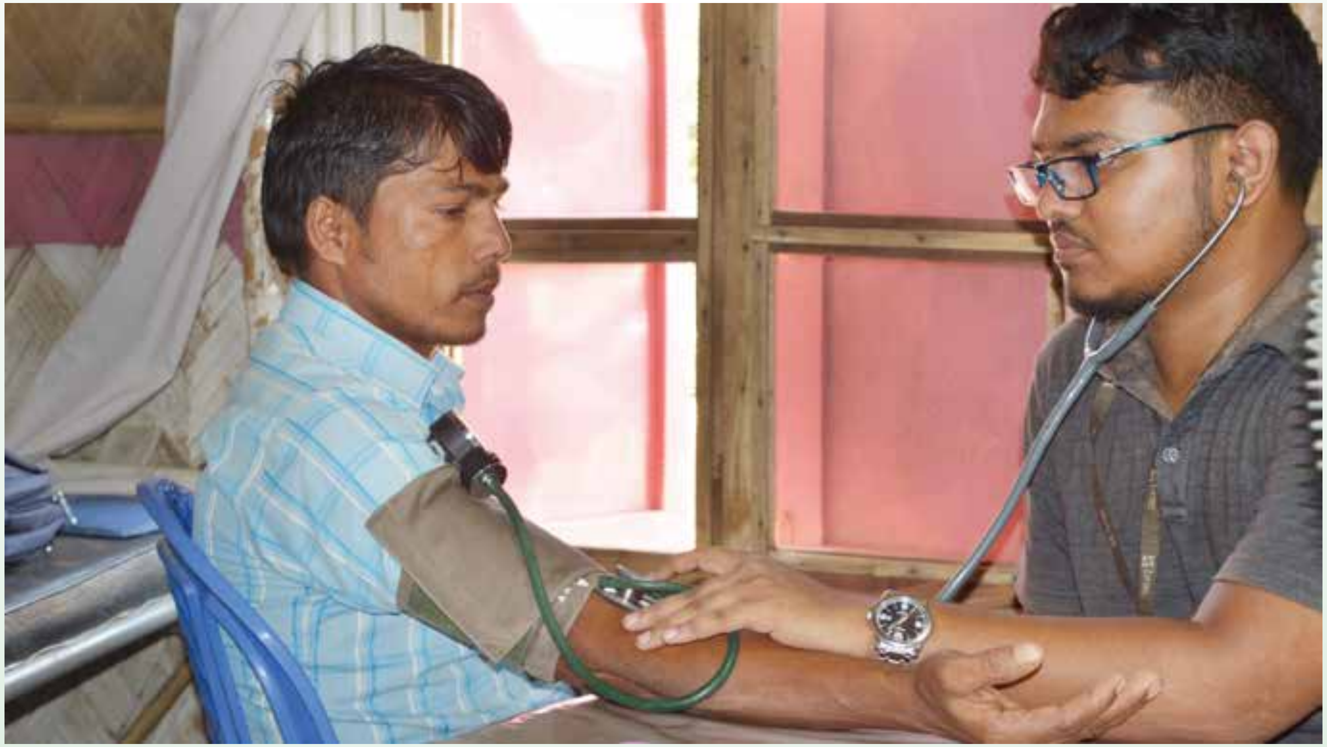

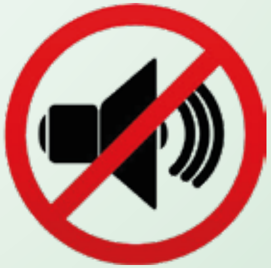

উচ্চরক্তচাপ নীরব ঘাতক !!!

FOR COMMUNITY

উচ্চ রক্তচাপের লক্ষণ সমূহ

বেশিরভাগ লোক মাথা ঘোরা বা মাথাব্যথাকে উচ্চ রক্তচাপের সাধারণ লক্ষণ হিসাবে ভাবেন। তবে উচ্চ রক্তচাপের বেশিরভাগ রোগীর রক্তচাপ খুব বেশি না হলে কোনো লক্ষণই থাকেনা।

উচ্চ রক্তচাপের সাধারণ লক্ষণসমূহের মধ্যে রয়েছেঃ

* মাথা ব্যাথা
* চোখে ঝাপসা দেখা
* মাথা ঘোরা
* কানে ভোঁ ভোঁ শব্দ শোনা
* শ্বাস প্রশ্বাস দ্রুত হওয়া
* নাক দিয়ে রক্ত পড়া

* উচ্চ রক্তচাপের লক্ষণ থাকেনা বলে, উচ্চ রক্তচাপের রোগীরা অনেকেই জানেন না তার উচ্চ রক্তচাপ আছে। যারা জানেন তারাও নিয়মিত চেকআপ করেন না, এমনকি তাদের রক্তচাপ কমানোর ঔষধ নিয়মিত খান না।
* কোনো লক্ষণ ছাড়াই উচ্চ রক্তচাপের কারনে স্ট্রোক, হার্ট অ্যাটাক, এমনকি মৃত্যুও হতে পারে।

  এ কারণেই উচ্চ রক্তচাপকে "নীরব ঘাতক" বলা হয়।

বেশিরভাগ উচ্চ রক্তচাপ রোগীর কোনও লক্ষণ থাকে না, এ বিষয়ের উপর জোর দিন।

উচ্চ রক্তচাপের বেশিরভাগ রোগীর কোনও লক্ষণই থাকেনা।
কারো কারো ক্ষেত্রে নিম্নের লক্ষণগুলো থাকতে পারে।

সাধারণ লক্ষণসমূহ
* মাথা ব্যাথা
* ঘাড়ে ব্যাথা
* চোখে ঝাপসা দেখা
* মাথা ঘোরা
* কানে ভোঁ ভোঁ শব্দ শোনা
* শ্বাস প্রশ্বাস দ্রুত হওয়া
* নাক দিয়ে রক্ত পড়া

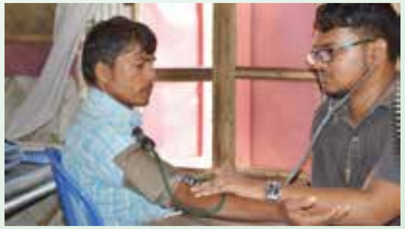

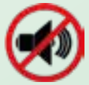

উচ্চরক্তচাপ নীরব ঘাতক !!!

FOR CHW

উচ্চ রক্তচাপের ফলে সৃষ্ট স্বাস্থ্য সমস্যা ও জটিলতা

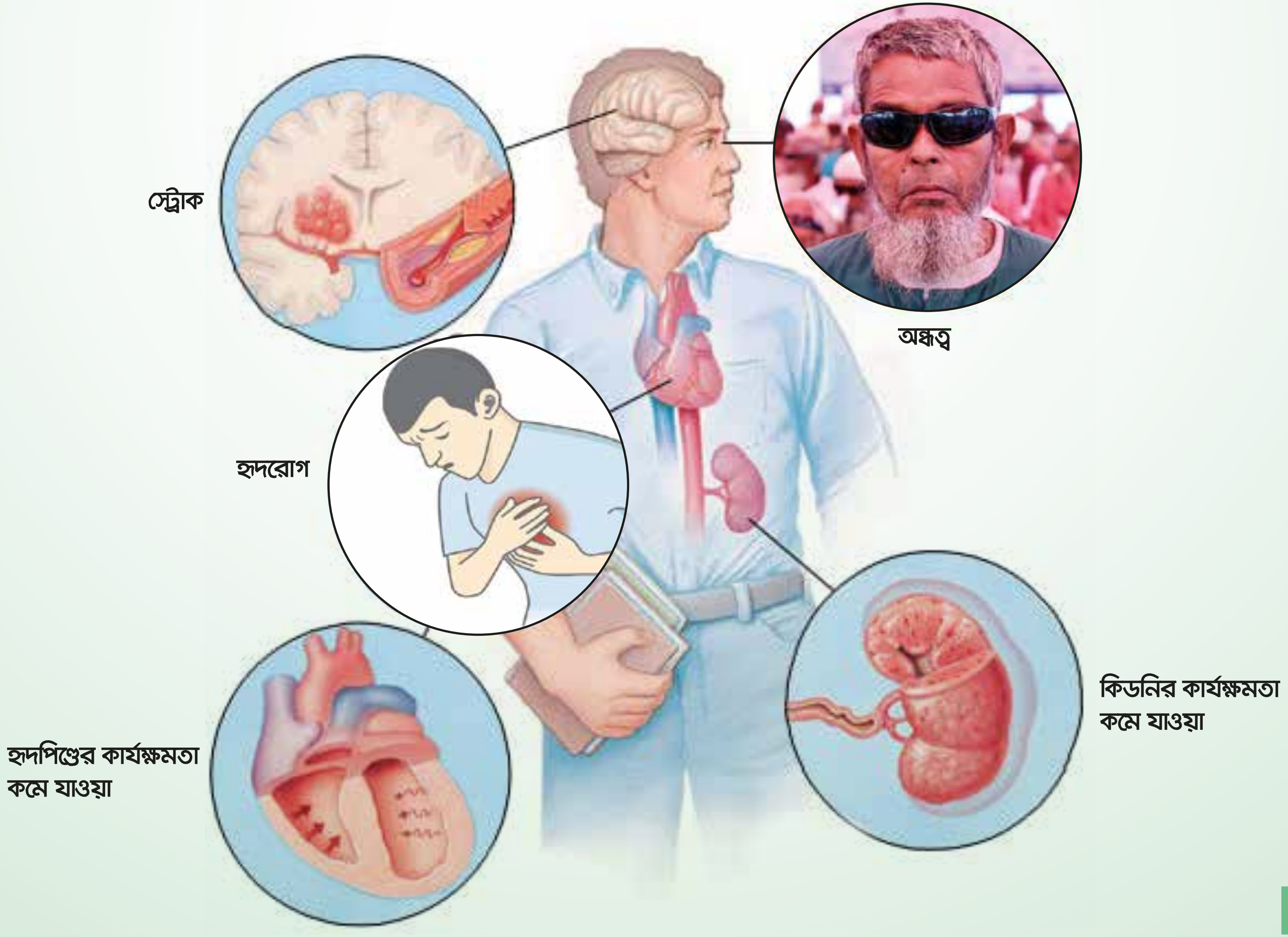

FOR COMMUNITY

উচ্চ রক্তচাপের ফলে সৃষ্ট স্বাস্থ্য সমস্যা ও জটিলতা

বেশিরভাগ ক্ষেত্রে, উচ্চ রক্তচাপের ফলে স্বাস্থ্য সমস্যা হতে লম্বা সময়ের প্রয়োজন হয়। উচ্চ রক্তচাপ যদি সনাক্ত না হয় (বা উচ্চরক্তচাপ অনিয়ন্ত্রিত থাকে), তাহলে যে সব সমস্যা দেখা দেয় তা হলঃ

স্ট্রোকঃ উচ্চ রক্তচাপ মস্তিষ্কের রক্তনালীগুলোকে সহজে বন্ধ করে দিতে পারে বা এমনকি রক্তনালী ফেটেও যেতে পারে। যার ফলে স্ট্রোক হয়।

দৃষ্টি শক্তি হ্রাসঃ উচ্চ রক্তচাপ চোখের রক্তনালীগুলিকে সঙ্কুচিত করে। যার ফলে দৃষ্টি শক্তি হ্রাস পায়।

হৃদরোগঃ উচ্চ রক্তচাপ ধমনীর ক্ষতি করে, যা পরবর্তীতে বন্ধ হয়ে যেতে পারে এবং হৃদপিণ্ডের পেশীতে রক্তের প্রবাহে বাঁধা দিতে পারে।

হৃদপিণ্ডের কার্যক্ষমতা কমে যাওয়াঃ উচ্চ রক্তচাপ হৃদপিণ্ডের কাজ বাড়িয়ে দেয়, যার ফলে হৃদপিণ্ডের আকার বেড়ে যায় এবং হৃদপিণ্ড দেহে রক্ত সরবরাহ করতে ব্যর্থ হতে পারে।

কিডনির রোগঃ উচ্চ রক্তচাপ কিডনির চারপাশের রক্তনালীর ক্ষতি করে এবং কার্যকরভাবে রক্ত পরিশোধন করার ক্ষমতাকে কমিয়ে দিতে পারে।

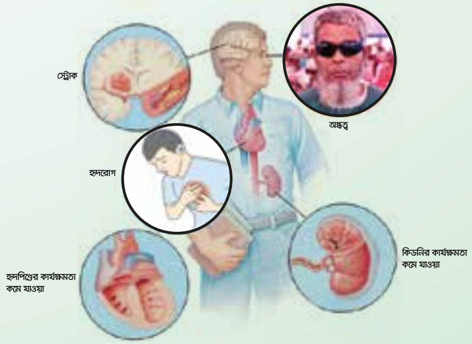

FOR CHW

উচ্চ রক্তচাপ নিয়ন্ত্রণের জন্য স্বাস্থ্যকর জীবনধারা

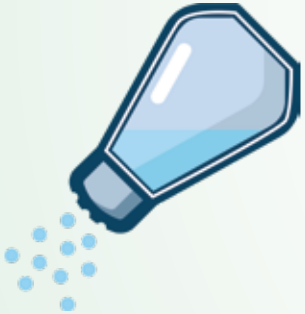
কম লবণ খাওয়া

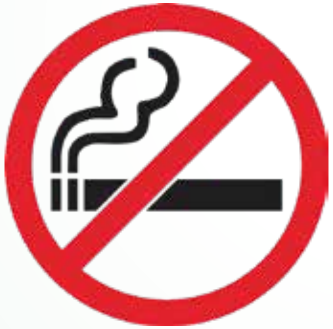
ধূমপান পরিহার করা

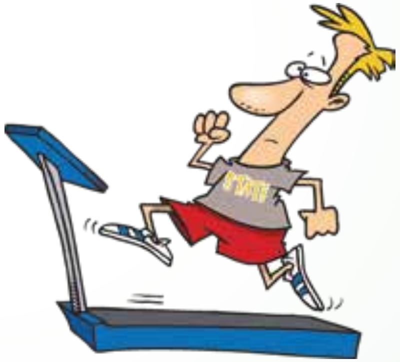
নিয়মিত শারীরিক পরিশ্রম

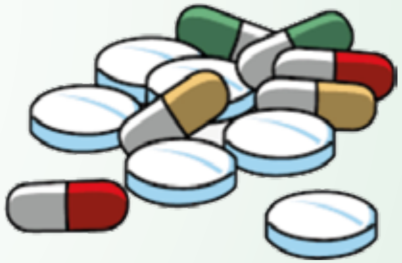
নিয়মিত ঔষধ খাওয়া

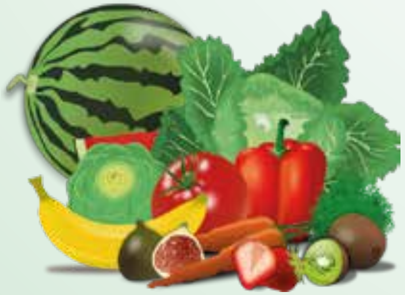
স্বাস্থ্যকর খাবার খওয়া

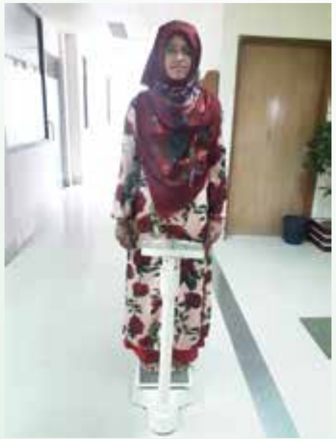
ওজন নিয়ন্ত্রণে রাখা

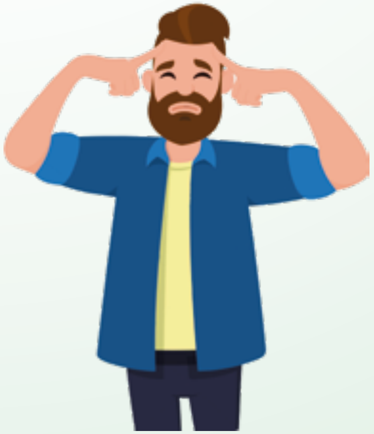
মানসিক চাপ পরিহার

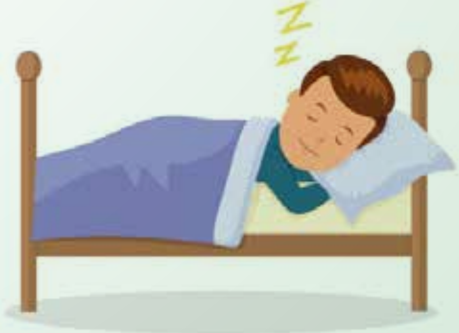

৬-৮ ঘণ্টা ঘুম

FOR COMMUNITY

উচ্চ রক্তচাপ নিয়ন্ত্রণের জন্য স্বাস্থ্যকর জীবনধারা

উচ্চ রক্তচাপ নিয়ন্ত্রণের দুটি উপায় রয়েছে: স্বাস্থ্যকর জীবনধারা অনুশীলন করা এবং নিয়মিত ওষুধ খাওয়া। স্বাস্থ্যকর জীবনযাত্রার মধ্যে রয়েছে কম লবণ খাওয়া, ধূমপান পরিহার করা, নিয়মিত শারীরিক পরিশ্রম করা, স্বাস্থ্যকর খাবার খাওয়া, ওজন নিয়ন্ত্রনে রাখা, মানসিক চাপ পরিহার করা ও ৬ থেকে ৮ ঘণ্টা ঘুমানো।

যদি আপনার রক্তচাপ খুব বেশি থাকে, আপনার চিকিৎসক আপনাকে সাথে সাথেই ওষুধ খাওয়ার পরামর্শ দিতে পারেন। ওষুধ খাওয়ার পাশাপাশি আপনাকে স্বাস্থ্যকর জীবনযাত্রা চালিয়ে যেতে হবে।

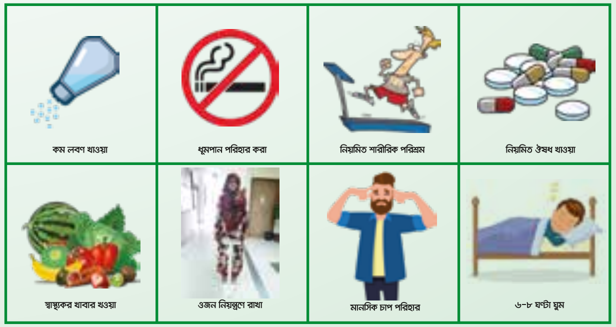

FOR CHW

উচ্চ রক্তচাপ হলে অবশ্যই মনে রাখবেন ...

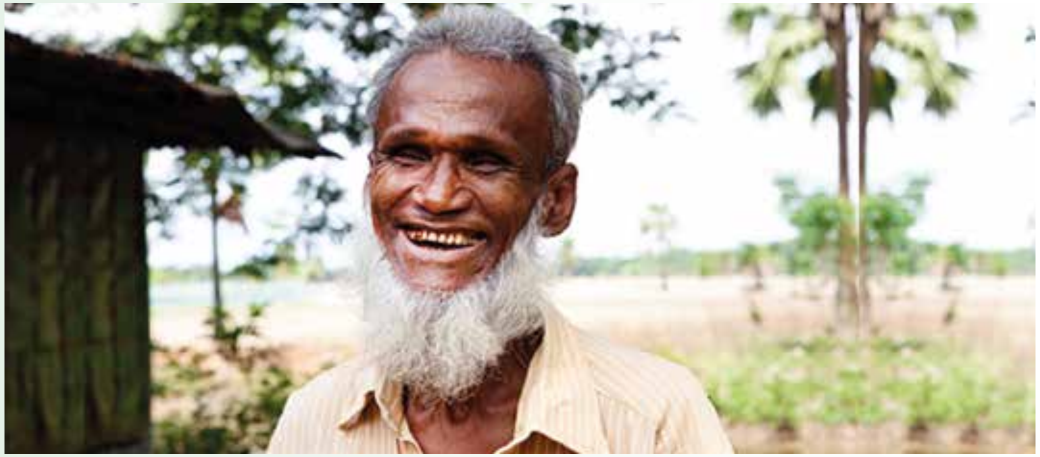

স্বাস্থ্যকর জীবন যাপন করা

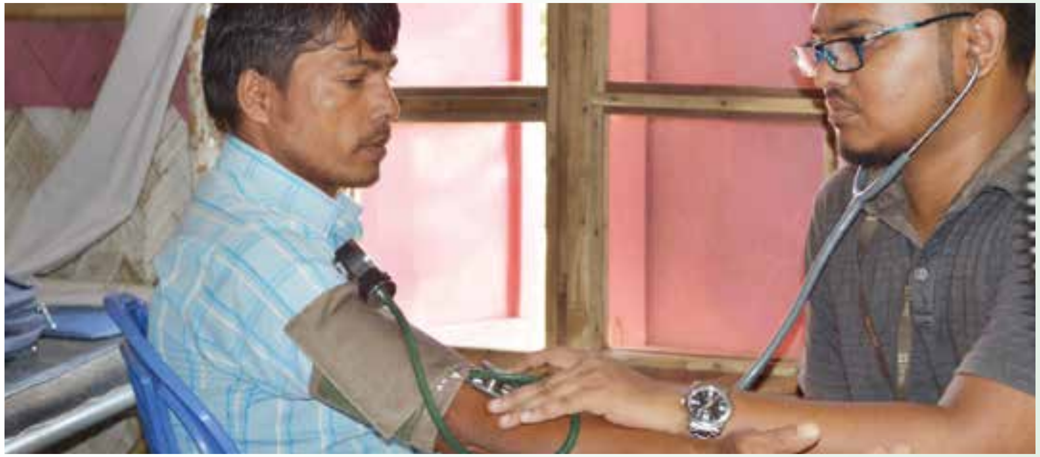

নিয়মিত রক্তচাপ পরিমাপ করানো

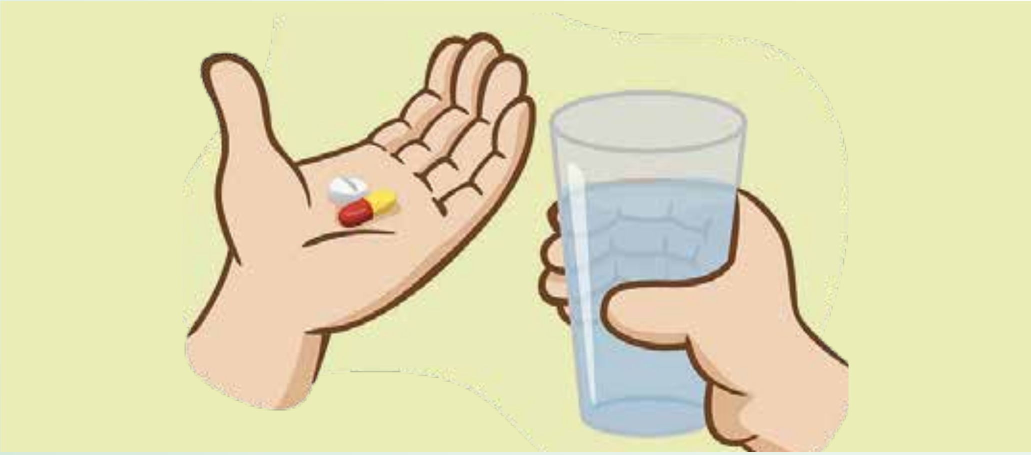

নিজেকে সুস্থ মনে হলেও, নিয়মিত ওষুধ খাওয়া

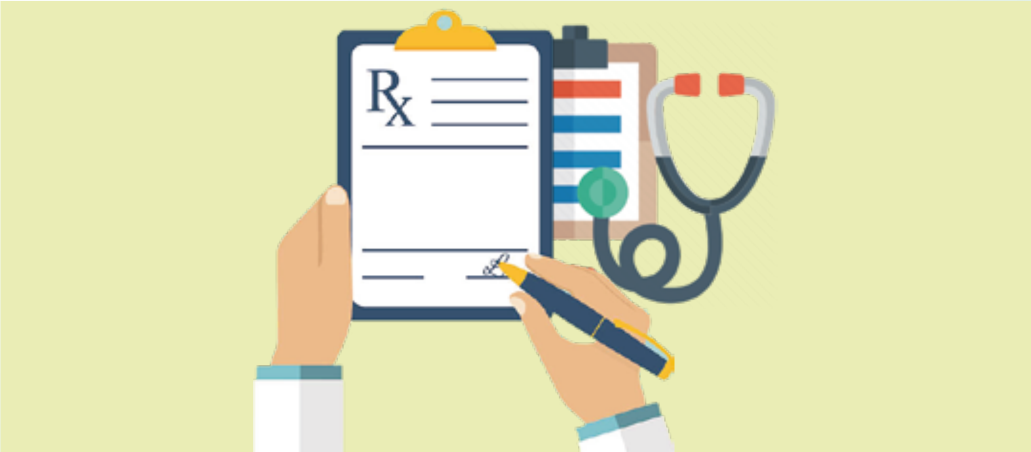

উচ্চরক্তচাপের জটিলতাগুলি সনাক্ত করতে
নিয়মিত চেকআপ করানো

FOR COMMUNITY

উচ্চ রক্তচাপ হলে অবশ্যই মনে রাখবেন ...

স্বাস্থ্যকর জীবন যাপন করা

নিয়মিত রক্তচাপ পরিমাপ করানো

নিজেকে সুস্থ মনে হলেও, নিয়মিত ওষুধ খাওয়া

উচ্চরক্তচাপের জটিলতাগুলি সনাক্ত করতে নিয়মিত চেকআপ করানো

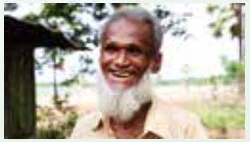

স্বাস্থ্যকর জীবন যাপন করা

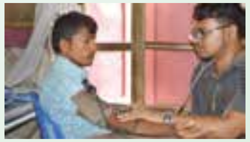

নিয়মিত রক্তচাপ পরিমাপ করানো

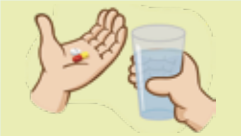

নিজেকে সুস্থ মনে হলেও, নিয়মিত ওষুধ খাওয়া

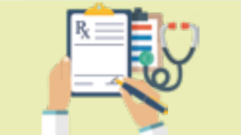

উচ্চরক্তচাপের জটিলতাগুলি সনাক্ত করতে নিয়মিত চেকআপ করানো

FOR CHW

World Health Organization
Bangladesh

Community Health Working Group

From the People of Japan

BRAC JAMES P GRANT SCHOOL OF PUBLIC HEALTH

BRAC UNIVERSITY
Inspiring Excellence
