# Supplementary figures and images for: Effectiveness of a community health worker-led low-sodium salt intervention to reduce blood pressure in rural Bangladesh: protocol for a cluster randomized controlled trial
Source: Trials. 2023 Jul 27;24:480. doi: 10.1186/s13063-023-07518-3 (PMC10375753; doi:10.1186/s13063-023-07518-3)

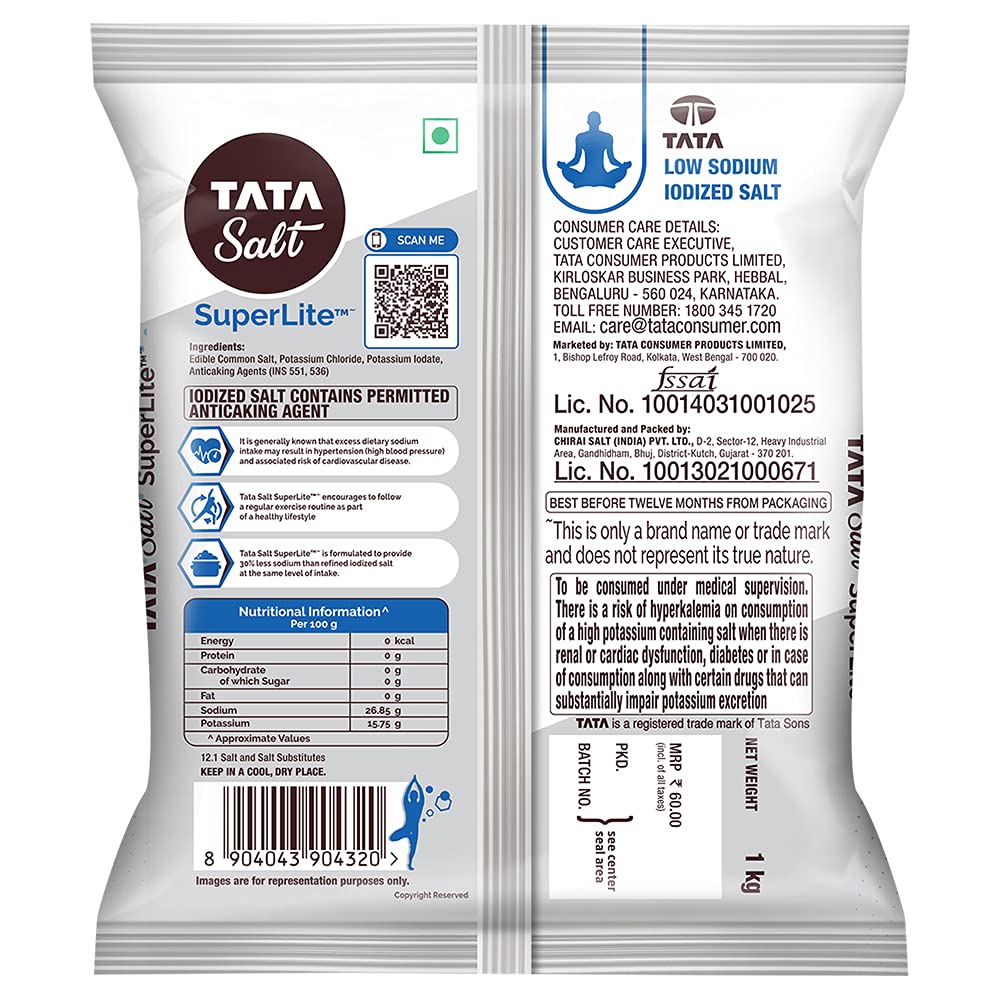

Supplement: Supplementary file 2 — Additional file 2. [file 13063_2023_7518_MOESM2_ESM.jpeg]
