# Supplementary material for: Effectiveness of a community health worker-led low-sodium salt intervention to reduce blood pressure in rural Bangladesh: protocol for a cluster randomized controlled trial
Source: Trials. 2023 Jul 27;24:480. doi: 10.1186/s13063-023-07518-3 (PMC10375753; doi:10.1186/s13063-023-07518-3)
Supplement: Supplementary file 3 — Additional file 3: Supplementary material 3. LSSS Intervention Study: Baseline Survey Questionnaire. [file 13063_2023_7518_MOESM3_ESM.docx]

LSSS Intervention Study: Baseline Survey Questionnaire

Contents

[1.0 Demographic and socioeconomic information 2](#_Toc120802286)

[2.0 Diet 4](#_Toc120802287)

[2.1 Fruits and vegetables 4](#_Toc120802288)

[2.2 Dietary salt 4](#_Toc120802289)

[2.3 Others 6](#_Toc120802290)

[3.0 Tobacco 7](#_Toc120802291)

[4.0 Alcohol 8](#_Toc120802292)

[5.0 Physical activity 9](#_Toc120802293)

[6.0 History of illness and management 10](#_Toc120802294)

[7.0 Lifestyle advice 11](#_Toc120802295)

[8.0 Caffeine intake 12](#_Toc120802296)

[9.0 Physical measurements: Blood pressure 13](#_Toc120802297)

[10.0 Physical measurements: Anthropometric 13](#_Toc120802298)

| **Question** | **Response** | **Code** |
| --- | --- | --- |
| Name of enumerator | **\|__\|__\|** | enumerator |
| Household ID | **\|__\|__\|** | a1 |
| Respondent ID | **\|__\|__\|** | a8 |
| Date | **\|__\|__\|** | A9 |

# Demographic and socioeconomic information

| **Question** | **Response** | | **Code** |
| --- | --- | --- | --- |
| Ask/observe whether this household or any person who lives in the household has the following items: | |  |  |
| Electricity | | Yes 1  No 2  Refused 88 | Cex1a |
| Flush toilet | | Yes 1  No 2  Refused 88 | Cex1b |
| Land phone | | Yes 1  No 2  Refused 88 | Cex1c |
| Mobile phone | | Yes 1  No 2  Refused 88 | Cex1d |
| Television | | Yes 1  No 2  Refused 88 | Cex1e |
| Refrigerator | | Yes 1  No 2  Refused 88 | Cex1g |
| Car | | Yes 1  No 2  Refused 88 | Cex1h |
| Moped/scooter/motorcycle/auto-rickshaw | | Yes 1  No 2  Refused 88 | Cex1i |
| Washing machine | | Yes 1  No 2  Refused 88 | Cex1j |
| Bicycle | | Yes 1  No 2  Refused 88 | Cex1k |
| Sewing machine | | Yes 1  No 2  Refused 88 | Cex1l |
| Almirah/wardrobe | | Yes 1  No 2  Refused 88 | Cex1m |
| Table | | Yes 1  No 2  Refused 88 | Cex1n |
| *Khat/chowki* | | Yes 1  No 2  Refused 88 | Cex1o |
| Chair/bench | | Yes 1  No 2  Refused 88 | Cex1p |
| Watch/clock | | Yes 1  No 2  Refused 88 | Cex1q |
| Computer/laptop/tablet | | Yes 1  No 2  Refused 88 | Cex1r |
| Domestic animal: cow/buffalo/goat | | Yes 1  No 2  Refused 88 | Cex1s |
| Shallow machine/ power tiller/ tractor | | Yes 1  No 2  Refused 88 | Cex1t |
| Rickshaw/Van - (own) | | Yes 1  No 2  Refused 88 | Cex1u |
| Electric Fan | | Yes 1  No 2  Refused 88 | Cex1u_n |
| What is the main material of the roof of the main house?  *[Record observation]*  *[If 1 household has more than 1 houses, ask the respondent which one of those is considered as the main house]* | | *Katcha* (bamboo/thatched/straw/gunny)1  Tin, tiles, or similar materials 2  Cement/concrete 3  Others (Please, specify) 96 | Cex2 |
| What is the main material of the floor of the main house?  *[Record observation]*  *[If 1 household has more than 1 houses, ask the respondent which one of those is considered as the main house]* | | Cement 1  Earth/Sand 2  Others (Please, specify) 96 | Cex2_a |
| What is the main material of the exterior wall of the main house?  *[Record observation]*  *[If 1 household has more than 1 houses, ask the respondent which one of those is considered as the main house]* | | Cement/concrete 1  Others (Please, specify) 96 | Cex2_b |
| What is the monthly average household income?  *[Consider the last 6 month’s income to decide average monthly estimate]* | | BDT **\|_______________________\|**  Refused 88 | Cinc |

# Diet

## Fruits and vegetables

The next questions I shall ask are about fruits and vegetables in your diet. I have a nutrition card that includes pictures of local fruits and vegetables. Each picture represents the size of a serving. Think of a typical week to answer these questions.

| **Question** | **Response** | **Code** |
| --- | --- | --- |
| In a typical week, on how many days do you eat fruits?  *[Use showcard 1]* | Number of days **\|__\|__\|**  Don’t know 77  *[If zero days, go to D3]* | D1 |
| How many servings of fruits do you eat on one of these days?  *[Use showcard 2]* | Number of servings **\|__\|__\| . \|__\|**  Don’t know 77 | D2 |
| In a typical week, on how many days do you eat vegetables?  *[Use showcard 3]* | Number of days **\|__\|__\|**  Don’t know 77  *[If zero days, go to Dx1]* | D3 |
| How many servings of vegetables do you eat on one of these days?  *[Use showcard 4]* | Number of servings **\|__\|__\| . \|__\|**  Don’t know 77 | D4 |
| What do you think is the desirable or recommended number of servings of fruits and vegetables one should have every day to remain healthy? | Number of servings **\|__\|__\| . \|__\|**  Don’t know 77 | Dx1 |

## Dietary salt

The next questions I shall ask are about the amount of salt in your diet. Dietary salt includes ordinary table salt, unrefined salt such as sea salt, iodized salt, salty rock cubes and powder, bit salt, testing salt, salty sauces, soya sauce, fish sauce, etc. (Use show cards 5 to 8).

The following questions are on adding salt to the food right before eating it. Besides, there are questions on controlling salt intake. Please, answer these questions even though you believe that you are on a diet that low in salt.

| **Question** | **Response** | **Code** |
| --- | --- | --- |
| How often do you add salt to your food right before eating it or as you are eating it?  *[Select one only]*  *[Use showcard 5]* | Always 1  Often 2  Sometimes 3  Rarely 4  Never 5  Don’t know 77 | D5a |
| How often do you add salty sauce such as soya sauce to your food right before eating it or as you are eating it?  *[e.g. Tomato sauce, tomato ketchup, chili sauce, soya sauce, fish sauce]*  *[Select one only]*  *[Use showcard 6]* | Always 1  Often 2  Sometimes 3  Rarely 4  Never 5  Don’t know 77 | D5b |
| How often do you eat processed food high in salt?  *[Processed food high in salt: food that has been altered from its natural state such as packaged salty snacks (e.g., chips, chanachur, jhal muri), canned salty food including pickles and preservatives, salty food prepared in a fast-food restaurant, cheese, processed meat, dried fish, salty fish, etc.]*  *[Use showcard 7]* | Always 1  Often 2  Sometimes 3  Rarely 4  Never 5  Don’t know 77 | D7 |
| How much salt do you think you consume?  *[Count all the sources of salt that the respondent consumes including salt used for meal preparation, extra intake, and others.]* | Far too much 1  Too much 2  Just the right amount 3  Too little 4  Far too little 5  Don’t know 77 | D8a |
| How much salty sauce do you think you consume?  *[e.g. Tomato sauce, tomato ketchup, chili sauce, soya sauce, fish sauce]*  *[Count all the sources of salt that the respondent consumes including salt used for meal preparation, extra intake, and others.]* | Far too much 1  Too much 2  Just the right amount 3  Too little 4  Far too little 5  Don’t know 77 | D8b |
| How much extra salt do you take in a typical day?  *[Use show card 8]* | **\|__\|__\| . \|__\|** Teaspoonful  Don’t know 77 | Dx2 |
| How important is it to lower the amount of salt in your diet? | Very important 1  Somewhat important 2  Not at all important 3  Don’t know 77 | D9 |
| What is the maximum amount of salt that a person should take in a day from all sources?  (Expressed in Teaspoonful)  *[Use show card 8]* | **\|__\|__\| . \|__\|** Teaspoonful  Don’t know 77 | Dx3 |
| What can too much salt or salty sauce can do to your health?  *[Multiple response]* | Nothing, more salt is good for one’s health  1  Increase blood pressure 2  Kidney disease 3  Asthma 4  Cancer 5  Tuberculosis 6  Others (Please, specify…) Dx4_others_  Don’t know 77 | Dx4/ Dx4_others_ |
| Currently, are you doing anything on a regular basis to control your salt intake? | Yes 1  No 2  Don’t know 77  *[If ‘No’, go to D12]* | Dx5 |
| Do you do any of the following on a regular basis to control your salt intake? |  |  |
| Limit consumption of processed food | Yes 1  No 2 | D11a |
| Look at the salt or sodium content on food labels | Yes 1  No 2 | D11b |
| Buy low-salt/ low-sodium substitutes | Yes 1  No 2 | D11c |
| Use spices other than salt while cooking | Yes 1  No 2 | D11d |
| Avoid eating food prepared outside home | Yes 1  No 2 | D11e |
| Stop/reduce having added salt | Yes 1  No 2 | D11f |
| Do other things specifically to control your salt intake | Yes 1  No 2  *[If ‘Yes’, go to D11_others_]* | D11g |
| Others (Please, specify) | **\|_________________________\|** | D11_others_ |
| Do you share salt with any other households? | Yes 1  No 2  *[If ‘No’, go to Dx6]* | D12 |
| Do you do that on a regular basis? | Yes 1  No 2 | D12a |
| How much salt do you need for your household on a monthly basis? | \|_____________\| in grams | D13_n |
| How much do you spend per month to purchase salt? | \|_____________\| in BDT | D14_n |

## Others

| **Question** | **Response** | **Code** |
| --- | --- | --- |
| What type of oil is used most often to cook in your household?  *[Select one only]* | Soybean oil 1  Palm oil 2  Sunflower oil 3  Mustard oil 4  Rice bran oil 5  *Dalda* 6  *Ghee*/Butter 7  None specific 8  Others (Please, specify…) Dx6_others_ | Dx6/Dx6_others_ |
| How often do you eat in a restaurant or take away in a typical week?  *[Applicable to any of breakfast, lunch, or dinner]* | **\|__\|__\|** Times  Don’t know 77 | Dx7 |
| How many times in a typical day (on average) do you eat snacks such as *singara, samosa, puri, chips, chanachur, fuchka/panipuri, chatpati, jhal muri,* salted biscuits, etc.? | **\|__\|__\|** Times  Don’t know 77 | Dx8 |
| How often do you eat red meat in a typical week? | **\|__\|__\|** Times  Don’t know 77 | Dx9 |
| How often do you consume dairy products (e.g., milk, cream, cheese, butter, and yoghurt) in a typical week? | **\|__\|__\|** Times  Don’t know 77 | Dx10 |

# Tobacco

| Do you currently smoke any tobacco product such as cigarette, *bidi*, *hookah*, cigar, or pipe?  *[Use showcard 14]* | Yes 1  No 2  [If No, go to T12] | T1 |
| --- | --- | --- |
| Do you smoke tobacco products daily?  *[Use showcard 14]* | Yes 1  No 2 | T2 |
| On average, how many of the following products do you smoke each day/week?  *[If less frequent than daily, record weekly]*  *[Record for each type]*  *[Use showcard 14]* | Daily Weekly |  |
|  | Manufactured **\|__\|__\|__\|__\| \|__\|__\|__\|__\|**  cigarettes | T5a/T5aw |
|  | *Bidi* **\|__\|__\|__\|__\| \|__\|__\|__\|__\|** | T5b/T5bw |
|  | *Hookah* ***\|__\|__\|__\|__\| \|__\|__\|__\|__\|***  */Dhaba* | T5c/T5cw |
|  | Pipes full **\|__\|__\|__\|__\| \|__\|__\|__\|__\|**  of tobacco | T5d/T5dw |
|  | Hand-rolled **\|__\|__\|__\|__\| \|__\|__\|__\|__\|**  cigarettes | T5e/T5ew |
|  | Cigar,  Cheroot, **\|__\|__\|__\|__\| \|__\|__\|__\|__\|**  Cigarillo | T5f/T5fw |
|  | No. of  shisha **\|__\|__\|__\|__\|\|__\|__\|__\|__\|**  sessions | T5g/T5gw |
|  | Others **\|__\|__\|__\|__\| \|__\|__\|__\|__\|** | T5h/T5hw |
|  | Others  (please, specify) **\|_________________\|** | T5others |
|  | Don’t know 77 |  |
| Do you currently use any smokeless tobacco product such as betel quid with *zarda*, *zarda* only, *zarda* with *supari*, betel quid with *shada pata*, *paan-masala* with tobacco, or chew *shada pata, gul, khoinee*, *nossi*, or *gutka*?  *[Use showcard 15]* | Yes 1  No 2  *[If No, go to A5]* | T12 |
| Do you use any smokeless tobacco product such as betel quid with *zarda*, *zarda* only, *zarda* with *supari*, betel quid with *shada pata*, *paan-masala* with tobacco, or chew *shada pata, gul, khoinee*, *nossi*, or *gutka* daily? | Yes 1  No 2 | T13 |
| On average, how many times do you use following products in a day/week?  *[If less frequent than daily, record weekly]*  *[Record for each type, use showcards]* | Daily Weekly |  |
|  | Betel quid  with *zarda*, **\|__\|__\|__\|__\| \|__\|__\|__\|__\|**  *zarda* only, or  *zarda* with *supari* | T14a/T14aw |
|  | Betel quid  with **\|__\|__\|__\|__\| \|__\|__\|__\|__\|**  *shada pata* | T14b/T14bw |
|  | *Paan-masala* **\|__\|__\|__\|__\| \|__\|__\|__\|__\|**  with tobacco | T14c/T14cw |
|  | *Shada pata*  *chewed* **\|__\|__\|__\|__\| \|__\|__\|__\|__\|** | T14d/T14dw |
|  | *Gul* **\|__\|__\|__\|__\| \|__\|__\|__\|__\|** | T14e/T14ew |
|  | *Khoinee* **\|__\|__\|__\|__\| \|__\|__\|__\|__\|** | T14f/T14fw |
|  | *Nossi*  **\|__\|__\|__\|__\| \|__\|__\|__\|__\|** | T14g/T14gw |
|  | Others **\|__\|__\|__\|__\| \|__\|__\|__\|__\|** | T14h/T14hw |
|  | Others  (please, specify) **\|_________________\|** | T14others |

# Alcohol

| Have you consumed any alcohol (e.g., beer, wine, spirit, *tari*, *cholai*, ram, *bangla*, chobani, carew, vodka, jin, etc.) within the past 30 days?  *[Use showcard 17 and 19]* | Yes 1  No 2  *[If ‘No’, go to mvpa1]* | A5 |
| --- | --- | --- |
| During the past 30 days, on how many occasions did you have at least one standard alcohol drink?  *[Use showcard 18]* | **\|__\|__\|** Number of occasions  Don’t know 77  *[If ‘0’, go to mvpa1]* | A6 |
| During the past 30 days, when you drank alcohol, how many standard drinks on average did you have during one drinking occassion? | **\|__\|__\|** Number of drinks per occasion  Don’t know 77 | A7 |
| During each of the past 7 days, how many standard drinks did you have each day?  *[Use showcard 18]* | Monday **\|__\|__\|**  Don’t know 77 | A10a |
|  | Tuesday **\|__\|__\|**  Don’t know 77 | A10b |
|  | Wednesday **\|__\|__\|**  Don’t know 77 | A10c |
|  | Thursday **\|__\|__\|**  Don’t know 77 | A10d |
|  | Friday **\|__\|__\|**  Don’t know 77 | A10e |
|  | Saturday **\|__\|__\|**  Don’t know 77 | A10f |
|  | Sunday **\|__\|__\|**  Don’t know 77 | A10g |

# Physical activity

The following questions concern how physically active you are in your free time and during transport (including your commute to and from work/school/classes)

| *MVPA, closed-ended*  On a typical week, how much time do you spend in total on moderate and vigorous activities? These include activities that increase your heartbeat and make you breathe faster (e.g., brisk walking, cycling as a means of transport or as exercise, heavy gardening, running, or recreational sports).  *[Only include activities that lasted at least 10 minutes at a time]*  *[Showcard 20a]* | Less than ½ an hour  (less than 30 minutes) 1  ½ an hour – 1 ½ hours  (30-90 minutes) 2  1 ½ – 2 ½ hours  (90-150 minutes) 3  2 ½ – 5 hours  (150-300 minutes) 4  More than 5 hours  (More than 300 minutes) 5 | mvpa1 |
| --- | --- | --- |
| *MVPA, open-ended*  On a typical week, how much time do you spend in total on moderate and vigorous activities where your heartbeat increases and you breathe faster (e.g., brisk walking, cycling as a means of transport or as exercise, heavy gardening, running, or recreational sports)?  *[Only include activities that lasted at least 10 minutes at a time]*  *[Showcard 20a]* | \|__\|__\| Hours per week  \|__\|__\| Minutes per week | mvpa2 |
| *VPA, closed-ended*  How much of the time that you spend on physical activities on a typical week, which you indicated above, do you spend in total on vigorous physical activities? These include activities that get your heart racing, make you sweat and leave you so short of breath that speaking becomes difficult (e.g., swimming, running, cycling at high speeds, cardio training, weight lifting, or team sports such as football).  *[Only include activities that lasted at least 10 minutes at a time]*  *[Showcard 20b]* | Less than ½ an hour  (less than 30 minutes) 1  ½ an hour - 1 hour  (30-60 minutes) 2  1 - 1 ½ hours  (60-90 minutes) 3  1 ½ - 2 ½ hours  (90-150 minutes) 4  More than 2 ½ hours  (More than 150 minutes) 5 | vpa1 |
| *VPA, open-ended*  How much of the time that you spend on physical activities on a typical week, which you indicated above, do you spend in total on vigorous physical activities? These include activities that get your heart racing, make you sweat and leave you so short of breath that speaking becomes difficult (e.g., swimming, running, cycling at high speeds, cardio training, weight lifting, or team sports such as football).  *[Only include activities that lasted at least 10 minutes at a time]*  *[Showcard 20b]* | \|__\|__\| Hours per week  \|__\|__\| Minutes per week | vpa2 |

# History of illness and management

| **Question** | **Response** | **Code** |
| --- | --- | --- |
| Have you ever had your blood pressure measured by a physician or any other health worker? | Yes 1  No 2 | H1 |
| Have you ever been told by a physician or any other health worker that you have **hypertension or your blood pressure is raised**? | Yes 1  No 2  *[If ‘No’, go to* H4x_n*]* | H2a |
| When were you told that? | Date **\|__\|__\| \|__\|__\| \|__\|__\|__\|__\|**  day month year | H2a1 |
| Have you ever been told by a physician or any other health worker you had a **heart condition**? | Yes 1  No 2 | H4x_n |
| Have you ever been told by a physician or any other health worker that you had a **stroke**? | Yes 1  No 2 | H5x |
| Have you ever been told by a physician or any other health worker that you have **diabetes**? | Yes 1  No 2 | H7a |
| Have you ever been told by a physician or any other health worker that your **blood cholesterol level is raised**? | Yes 1  No 2 | H13a |
| Have you ever been told by a physician or any other health worker that you have **liver condition**? | Yes 1  No 2 | H2a5 |
| Have you ever been told by a physician or any other health worker that you have **thyroid condition**? | Yes 1  No 2 | H2a6 |
| Since the last survey (enrollment survey), are you taking any new drug/medication? | Yes 1  No 2  *[If ‘No’, go to H20]* | H1x |
| Are the medicines or prescriptions available? | Prescription only 1  Medicine only 2  Both prescription and medicine available 3 | H1x_n |
| Which new drugs/medications are you currently taking?  *[Record the name and dosage of each of the drugs]* |  | H2x |
| Drug 1 | Name 1 | H2xn1 |
|  | Dosage 1 | H2xd1 |
|  | Type of Medicine | H2xn_n1 |
| Drug 2 | Name 2 | H2xn2 |
|  | Dosage 2 | H2xd2 |
|  | Type of Medicine | H2xn_n2 |
| Drug 3 | Name 3 | H2xn3 |
|  | Dosage 3 | H2xd3 |
|  | Type of Medicine | H2xn_n3 |
| Drug 4 | Name 4 | H2xn4 |
|  | Dosage 4 | H2xd4 |
|  | Type of Medicine | H2xn_n4 |
| Drug 5 | Name 5 | H2xn5 |
|  | Dosage 5 | H2xd5 |
|  | Type of Medicine | H2xn_n5 |
| Picture of prescription  *[if prescription is available]* |  | Jpg_presc |

# Lifestyle advice

| During the past 12 months, have you visited a physician or any other health worker? | Yes 1  No 2  *[If ‘No’, go to M4a]* | H20 |
| --- | --- | --- |
| During any of your visits to a physician or any other health worker in the past 12 months, were you advised to do any of the following?  *[Record for each]* | | |
| Quit using tobacco or not to start | Yes 1  No 2 | H20a |
| Reduce salt in your diet | Yes 1  No 2 | H20b |
| Eat at least five servings of fruits and vegetables each day | Yes 1  No 2 | H20c |
| Reduce fatty food in your diet | Yes 1  No 2 | H20d |
| Start or do more physical activity | Yes 1  No 2 | H20e |
| Maintain a healthy body weight or lose weight | Yes 1  No 2 | H20f |
| Reduce sugary beverages in your diet | Yes 1  No 2 | H20g |

# Caffeine intake

| Have you consumed any of the following items today? | Yes 1  No 2  *[If ‘No’, go to M4a]* | Caf1 |
| --- | --- | --- |
| Coffee | Yes 1  No 2 | Caf1a |
| Measure the quantity in a standard cup  *[Record in quarter intervals (e.g., .25, .5, .75, 1, 1.25, 1.5, 1.75, 2, etc.) and round to the nearest]*  *[Don’t know 77]* |  |  |
| Tea/ Iced tea | Yes 1  No 2 | Caf1b |
| Measure the quantity in a standard cup  *[Record in quarter intervals (e.g., .25, .5, .75, 1, 1.25, 1.5, 1.75, 2, etc.) and round to the nearest]*  *[Don’t know 77]* |  |  |
| Chocolate  (e.g., hot chocolate, chocolate milk, chocolate bar, candy bar, mint, gum)  *[Don’t know 77]* | Yes 1  No 2 | Caf1c |
| Record the total caffeine quantity (in mg) mentioned on the labels |  |  |
| Soft drinks/ Energy drinks/ Soda  e.g., Coca-Cola, Dr. Pepper, Mountain Dew, Red Bull, or other local brands  *[Don’t know 77]* | Yes 1  No 2 | Caf1d |
| Record the intake in mililitres *(cross-check with the volumes of provided standard containers, e.g., 250 ml, 500 ml, etc.)* |  |  |
| Ice-cream  *[Except vanilla ice cream]*  *[Don’t know 77]* | Yes 1  No 2 | Caf1f |
| Record the total caffeine quantity (in mg) mentioned on the labels |  |  |
| Measure the quantity in a standard cup  *Record in quarter intervals (e.g., .25, .5, .75, 1, 1.25, 1.5, 1.75, 2, etc.) and round to the nearest* |  |  |
| When was the last time today have you taken those?  *[Record for each]* | Time **\|__\|__\| \|__\|__\|**  hour minute | Caf2 |

# Physical measurements: Blood pressure

| **Question** | **Response** | **Code** |
| --- | --- | --- |
| Device ID | **\|__\|__\|__\|** | M40 |
| Reading 1 | Systolic (mm Hg) **\|__\|__\|__\|** | M4a |
|  | Diastolic (mm Hg) **\|__\|__\|__\|** | M4b |
|  | Heart rate (beats/minute) **\|__\|__\|__\|** | M16a |
| Reading 2 | Systolic (mm Hg) \|__\|__\|__\| | M5a |
|  | Diastolic (mm Hg) \|__\|__\|__\| | M5b |
|  | Heart rate (beats/minute) **\|__\|__\|__\|** | M16b |
| Reading 3 | Systolic (mm Hg) **\|__\|__\|__\|** | M6a |
|  | Diastolic (mm Hg) **\|__\|__\|__\|** | M6b |
|  | Heart rate (beats/minute) **\|__\|__\|__\|** | M16c |

# Physical measurements: Anthropometric

| **Question** | **Response** | **Code** |
| --- | --- | --- |
| Device ID | **\|__\|__\|__\|** | Am0 |
| Reading 1 | Height \|__\|__\|__\| in cm | Am1 |
|  | Weight **\|__\|__\|__\|** in kg | Am2 |
| Reading 2 | Height \|__\|__\|__\| in cm | Am3 |
|  | Weight **\|__\|__\|__\|** in kg | Am4 |
| Reading 3 | Height \|__\|__\|__\| in cm | Am5 |
|  | Weight **\|__\|__\|__\|** in kg | Am6 |
