# Supplementary material for: Effectiveness of a community health worker-led low-sodium salt intervention to reduce blood pressure in rural Bangladesh: protocol for a cluster randomized controlled trial
Source: Trials. 2023 Jul 27;24:480. doi: 10.1186/s13063-023-07518-3 (PMC10375753; doi:10.1186/s13063-023-07518-3)
Supplement: Supplementary file 5 — Additional file 5: Supplementary material 5. Low-Sodium Salt Substitute Intervention: Consent Form. [file 13063_2023_7518_MOESM5_ESM.docx]

Low-Sodium Salt Substitute Intervention

# CONSENT FORM

## Purpose of the research

Greetings!

We are from the BRAC James P Grant School of Public Health (JPGSPH), BRAC University, and we are conducting research to assess the effectiveness of a low-sodium salt substitute intervention in reducing blood pressure among the people of this district with a view to preventing hypertension and its complications in this region. This programme will be implemented in Rampur Union of Parbatipur Sub-district (Dinajpur District, Rangpur Division) with the financial assistance of the University of Stanford (Stanford, California, US).

We believe that your participation in this study will help us effectively assess whether our intervention would contribute to reducing blood pressure as well as preventing hypertension and its complications. Furthermore, the findings of this study are expected to inform us whether we can scale up the intervention across the entire country or even beyond its border.

## Why invited to participate in the study?

You are being asked to participate in this study because you are a permanent resident of this area. Moreover, you are at least 18 years of age. We believe that you can help us in this research by providing valuable information.

## What is expected from you in this study?

If you agree to participate in this study, we shall take 30-50 minutes of your time for an interview. We would like to ask you some questions regarding your sociodemographic and economic information and information regarding your diet, overall health status, and healthcare seeking behaviour. Besides, we would like to collect your blood pressure measurements. This will help us conduct the study in the best possible manner to meet our research objective. Besides, your recommendations (should you have any) will broaden the horizon of our knowledge in terms of how we can better implement the programme in the future. Only the investigators of the study will have access to your information. However, you can be assured that you can withdraw yourself from the study anytime without any constrain from the researchers.

## Risk

There is little or no risk related to your participation in this study. The topics of the interview will include (as I have mentioned before): your sociodemographic and economic information, diet, overall health status, and healthcare seeking behaviour, and blood pressure measurements. However, you can skip any question if you find yourself uncomfortable in answering it.

## Benefits

Participation in this study may not benefit you directly. Having said that, the information that you will share will be very useful to the further improvement of health status of your area.

## Privacy, anonymity, and confidentiality

We would keep all the information confidential. None other than the investigators of this research will have access to it. All the information will be kept in a locked cabinet in our Dhaka office. We shall not record or use you name anywhere in the study; we shall use a number instead of your name to keep your information confidential.

## Future use of information

Anonymous or abstracted information and data may be shared with other researchers within and outside the country. However, this will not conflict with or violate the maintenance of privacy, anonymity and confidentiality of information identifying participants in any way.

## Right not to participate and withdraw

Your participation in the study is voluntary, and you have the sole authority to decide for or against your participation. You would also be able to withdraw from participation any time during the study, without showing any cause. Refusal to participate in the study or withdraw yourself from the study will not cause you any harm, and it will not cause any change in your regular healthcare seeking from different government and private facilities.

## Principle of compensation

As mentioned earlier, your participation in this study is completely voluntary and you will not get any payment for participating in this study.

## Answering your questions/ Contact persons

Prof Malabika Sarker, Professor and Associate Dean of the BRAC JPGSPH, BRAC University, is one of the Principal Investigators of the study. If you have any question about this study, you can communicate directly with her via telephone calls [INSERT TELEPHONE NUMBER]. Thank you very much for your cooperation.

## Participant

I certify that the aforementioned information was adequately explained to me and I understood the explanations.

________________________________________ _____________________________

Signature or left thumb impression of participant Date

_______________________________________ _____________________________

Signature of the PI or his/her representative Date

*[NOTE: In case of representative of the PI, s/he will put her/his full name and designation and then sign]*
