# Supplementary material for: Effectiveness of a community health worker-led low-sodium salt intervention to reduce blood pressure in rural Bangladesh: protocol for a cluster randomized controlled trial
Source: Trials. 2023 Jul 27;24:480. doi: 10.1186/s13063-023-07518-3 (PMC10375753; doi:10.1186/s13063-023-07518-3)
Supplement: Supplementary file 4 — Additional file 4. [file 13063_2023_7518_MOESM4_ESM.pdf]

Stanford | King Center on Global Development

February 5, 2021

Dear Professor Geldsetzer,

Thank you for your application to the King Center on Global Development's Junior Faculty Research Grant program for the project entitled 'Community health workers to achieve uptake of low-sodium salt: A proof-of-concept trial in rural Malaysia.' Our selection committee has concluded its review of proposals, applying a general filter for alignment with the general themes of global poverty and development, and using the following criteria to evaluate the proposals: (1) innovation, (2) academic rigor, and (3) whether the budget is commensurate with the value of the proposed research.

We are delighted to notify you that your application has been selected for an award of up to $55,000. The funds are intended to cover 25% of the postdoc costs as well as all equipment and consumables costs and most other costs. The King Center will also cover the 8% university infrastructure charge on this award. The funds will be transferred within one month after you notify us of the account PTA created within your department (or a department of your choice).

The conditions and deadline attached to this award are:

- The award shall be used for the purposes outlined in your proposal.
- **You are required to request permission prior to deviations from your original proposal or budget.**
- You will be required to provide a status report summarizing progress toward your research goals, as well as an accounting of funds spent, by **July 1, 2022**.

Please note:

- One no-cost extension for a duration of one year may be requested.
- The principal investigator is responsible for ensuring compliance with Stanford research policy, including human subjects protocols and special requirements for carrying out international work.
- Please refer to the Dean of Research website at http://doresearch.stanford.edu/ for all relevant policy and contact information.

Your award is intended to support new research on important questions related to global development and poverty. We look forward to engaging you either as an attendee or participant in King Center community-building activities in the coming year, including seminars, workshops, and conferences.

Please don't hesitate to contact me at jleino@stanford.edu regarding the award, or Administration and Financial Manager Irina Lechtchinskaia at irinal1@stanford.edu if you have any questions about the fund transfer process.

Congratulations and we look forward to hearing more about your research as it progresses.

Sincerely,

Jessica Leino, PhD
Executive Director and Research Scholar
Stanford King Center on Global Development

Please indicate your acceptance of this award and agreement to comply with human subjects and other Stanford University research-related policies by signing below and returning a signed copy to lgoddi@stanford.edu by February 12, 2021.

___________________________________	____________________________	_____________
Principal investigator's name (Printed)	*Signature*	*Date*
